# Supplementary figures and images for: Power and sample size for reversible linear mixed models with clustering and longitudinality: GLIMMPSE Version 3
Source: PLoS One. 2025 Sep 3;20(9):e0329712. doi: 10.1371/journal.pone.0329712 (PMC12407473; doi:10.1371/journal.pone.0329712)

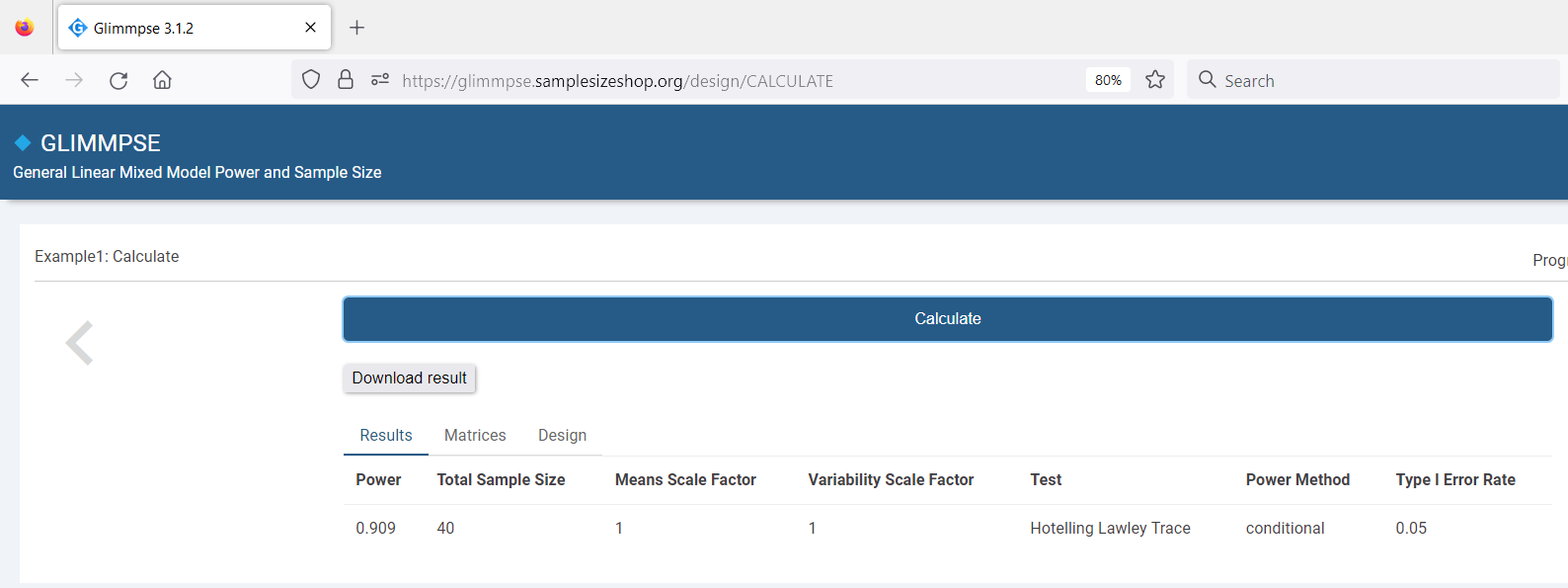

Supplement: S1 File — (ZIP) [file pone.0329712.s001.zip › SupplementaryMaterialA/Example1Calculate.png]

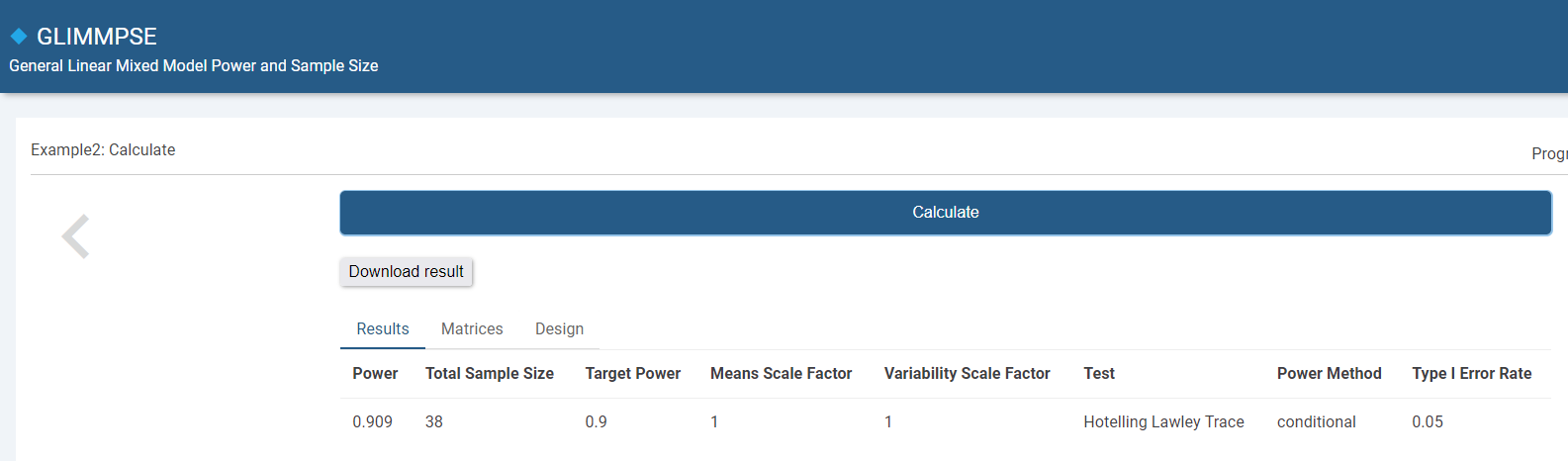

Supplement: S1 File — (ZIP) [file pone.0329712.s001.zip › SupplementaryMaterialA/Example2Calculate.png]

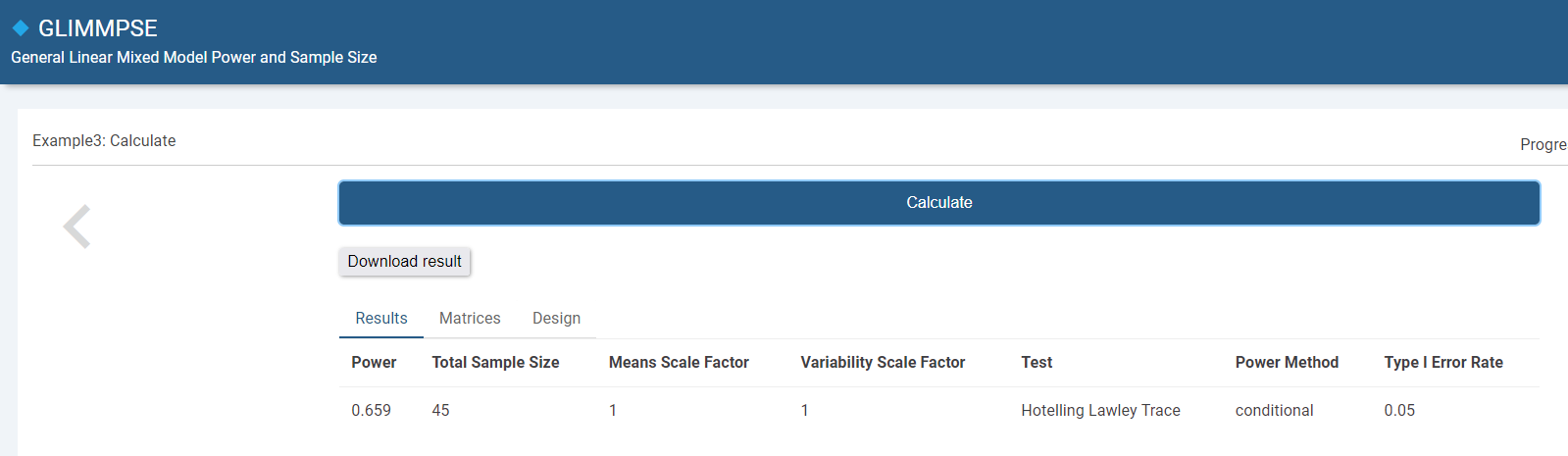

Supplement: S1 File — (ZIP) [file pone.0329712.s001.zip › SupplementaryMaterialA/Example3Calculate.png]

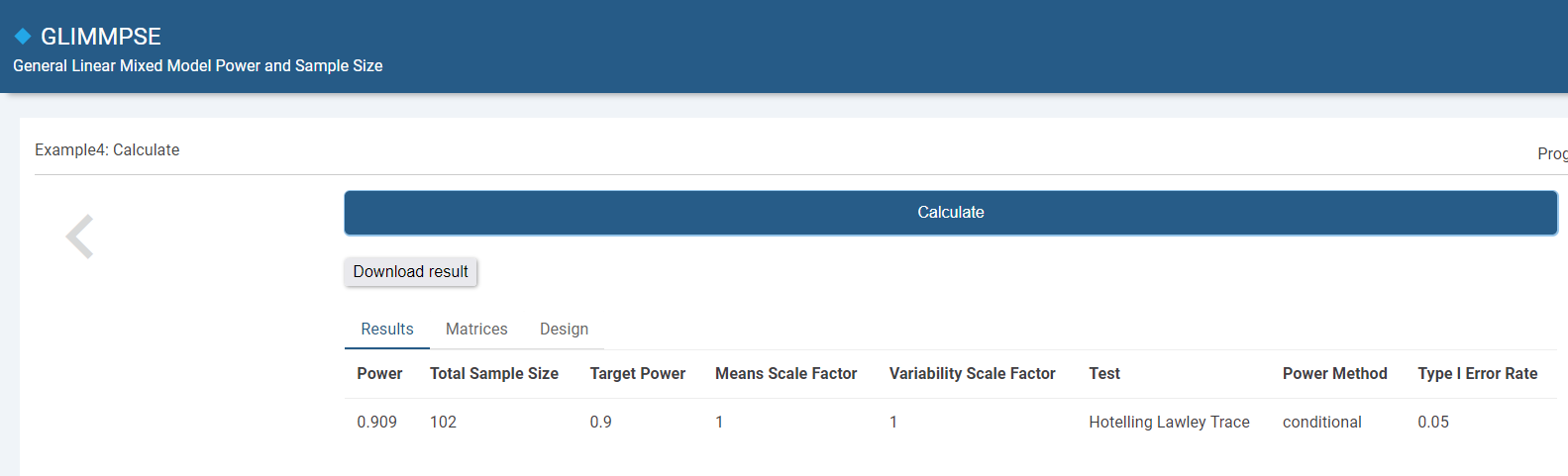

Supplement: S1 File — (ZIP) [file pone.0329712.s001.zip › SupplementaryMaterialA/Example4Calculate.png]

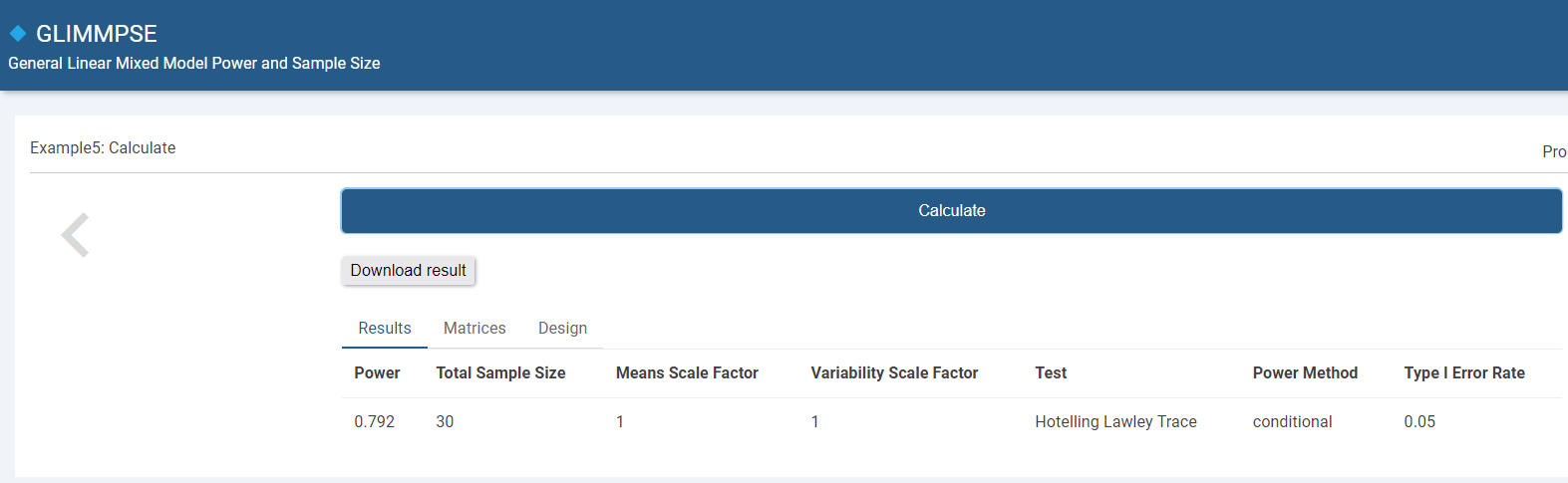

Supplement: S1 File — (ZIP) [file pone.0329712.s001.zip › SupplementaryMaterialA/Example5Calculate.png]
