# Supplementary material for: Power and sample size for reversible linear mixed models with clustering and longitudinality: GLIMMPSE Version 3
Source: PLoS One. 2025 Sep 3;20(9):e0329712. doi: 10.1371/journal.pone.0329712 (PMC12407473; doi:10.1371/journal.pone.0329712)
Supplement: S2 Text — (PDF) [file pone.0329712.s002.pdf]

Supplementary Material B: Search Terms and Grant List

1 Section Description

Table 1: Report Section Descriptions

Section	Description
Search Strategy	Boolean search terms
User Survey Set	Grant numbers from user surveys
Citers Set	Grant numbers extracted from citing publications
Excluded Grants	NCATS CTSI grants and non-NIH federal funders that were excluded

2 Search Strategy

The following exact Boolean search strategy was employed:

((”Funding” OR ”Supported” OR ”Funded”)
NEAR:100
(”National Institutes of Health” OR
”National Institute of Mental Health” OR ”NIMH” OR
”National Institute of Neurological Disorders and Stroke” OR ”NINDS” OR
”National Institute of Child Health and Human Development” OR ”NICHD” OR
”National Institute on Aging” OR
”National Institute on Alcohol Abuse and Alcoholism” OR ”NIAAA” OR
”National Institute on Drug Abuse” OR ”NIDA” OR
”National Cancer Institute” OR
”National Heart, Lung, and Blood Institute” OR ”NHLBI” OR
”National Institute of Diabetes and Digestive and Kidney Diseases” OR ”NIDDK” OR
”National Institute of Allergy and Infectious Diseases” OR ”NIAID” OR
”National Institute of General Medical Sciences” OR ”NIGMS” OR
”National Institute of Environmental Health Sciences” OR ”NIEHS” OR
”National Eye Institute” OR
”National Institute on Deafness and Other Communication Disorders” OR ”NIDCD” OR
”National Institute of Dental and Craniofacial Research” OR ”NIDCR” OR
”National Institute of Arthritis and Musculoskeletal and Skin Diseases” OR ”NIAMS” OR
”National Institute of Biomedical Imaging and Bioengineering” OR ”NIBIB” OR
”National Institute of Nursing Research” OR ”NINR” OR
”National Institute on Minority Health and Health Disparities” OR ”NIMHD” OR
”National Center for Complementary and Integrative Health” OR ”NCCIH” OR
”National Center for Advancing Translational Sciences” OR ”NCATS” OR
”Fogarty International Center” OR
”National Library of Medicine” OR
”Clinical Center” OR

"Center for Scientific Review" OR "CSR" OR
"Center for Information Technology" OR
"Department of Veterans Affairs" OR "Veterans Affairs" OR
"VA Medical Research" OR "VA Merit Review" OR "VA Career Development" OR
"VA Research & Development" OR
"Centers for Disease Control and Prevention" OR "CDC" OR
"Agency for Healthcare Research and Quality" OR "AHRQ" OR
"Food and Drug Administration" OR "FDA" OR
"Substance Abuse and Mental Health Services Administration" OR "SAMHSA" OR
"Health Resources and Services Administration" OR "HRSA" OR
"National Center of Neuromodulation for Rehabilitation" OR "NCNR" OR
"Department of Defense" OR "CDMRP" OR "Congressionally Directed Medical Research Programs" OR
"Department of Energy"))
AND
("Pilot Grant" OR "VA Quality" OR
"AR0" OR R00 OR R01 OR R03 OR R15 OR R21 OR R25 OR R33 OR R34 OR R37 OR
K01 OR K08 OR K23 OR K99 OR
U01 OR U18 OR U19 OR U24 OR U54 OR UL1 OR
P01 OR P30 OR
T32 OR F31 OR F32))
AND NOT
(("Post hoc" OR "post hoc" OR "post-hoc")
NEAR:100
("power" OR "sample size"))

3 Grant Numbers by Source

3.1 Set of Grants from User Surveys

Table 2: Grants Reported Through User Surveys (n=11)

**Grant Number**
F32DK127867
K01MH130752
R01DA054236
R01DK130929
R01HD107060-01A1
R01MH132071
R01MH137064
R21AI142483
R21MH131787
R43MH130293-01A1
UH3CA260607

3.2 Set of Grants Acknowledged by Citers

Table 3: Grants Acknowledged by GLIMMPSE Citers (n = 70)

**Grant Numbers**			
F31DA043303	K01AG050707	K01DA040043	K01DA04295
K01HD068170	K01HL143137	K12HD093427	K23048160
K23MH111977	K23MH119225	K24MH121571	KL2TR001426
L30MH111037	P01HL120840	P30AG019610	R01AA020695
R01AG041202	R01AG054077	R01AG057901	R01AG083925
R01DE025946	R01DK107761	R01DK108642	R01DK130900
R01GM121081	R01HD087672	R01HD093694	R01HL133293
R01HL137112	R01HL142808	R01HL146914	R01HL156993
R01HL157407	R01HL158686	R01MH097320	R01MH103241
R01MH108590	R01MH108657	R01MH112558	R01MH113857
R01MH114891	R01MH115715	R01MH116038	R01MH119225
R01NR014451	R03DC012135	R21AG052821	R21HD076092-01A1
R21MH100250	R21TR002402	R25GM111901	R25GM111901-04S1
R33MH100250	R34MH113613	R42AR074897	T32AG000213
T32AG020499	T32HD057850	T32HL134621	T32MH062994
TL1TR001431	TL1TR002368	TL1TR002375	U10NS086513
U54CA180908	UH2AG050312		

4 Excluded Grants

4.1 NCATS CTSI Grants

The following NCATS Clinical and Translational Science Institute grants were excluded:

- UL1TR000077
- UL1TR001425
- UL1TR002535

4.2 Non-NIH Federal Funders

The following non-NIH federal grants were excluded:

- 27-IR-0034
- QUE20–017
- AR070363
- BCS-2122112
- P2CHD086844
