# Supplementary material for: Power and sample size for reversible linear mixed models with clustering and longitudinality: GLIMMPSE Version 3
Source: PLoS One. 2025 Sep 3;20(9):e0329712. doi: 10.1371/journal.pone.0329712 (PMC12407473; doi:10.1371/journal.pone.0329712)
Supplement: S3 Text — (PDF) [file pone.0329712.s003.pdf]

Supplementary Material C: Software Citers

[1] Abdu, A. O. and Abebaw Mekonnen, B. (2021). Episodes of Undernutrition and its Predictors among Clients on Antiretroviral Treatment in Southwest Ethiopia: A Record Review. *HIV/AIDS (Auckland, N.Z.)*, 13:61–71.

[2] Abdulatif, M., Mukhtar, A., and Obayah, G. (2015). Pitfalls in reporting sample size calculation in randomized controlled trials published in leading anaesthesia journals: a systematic review. *British Journal of Anaesthesia*, 115(5):699–707. WOS:000363934700009.

[3] Achiron, A., Hecht, I., Juza, C., Barak, A., and Burgansky-Eliash, Z. (2018). The effect of sildenafil on retinal blood velocity in healthy subjects. *Eye and Vision*, 5:UNSP 30. WOS:000457417400001.

[4] Adams, T. G., Cisler, J. M., Kelmendi, B., George, J. R., Kichuk, S. A., Averill, C. L., Anticevic, A., Abdallah, C. G., and Pittenger, C. (2021). Transcranial direct current stimulation targeting the medial prefrontal cortex modulates functional connectivity and enhances safety learning in obsessive-compulsive disorder: Results from two pilot studies. *Depression and Anxiety*, n/a(n/a). _eprint: https://onlinelibrary.wiley.com/doi/pdf/10.1002/da.23212.

[5] Adey-Wakeling, Z., Jolliffe, L., O'Shannessy, E., Hunter, P., Morarty, J., Cameron, I. D., Liu, E., and Lannin, N. A. (2021). Activity, Participation, and Goal Awareness After Acquired Brain Injury: A Prospective Observational Study of Inpatient Rehabilitation. *Annals of Rehabilitation Medicine*, 45(6):413–421.

[6] Aguilar-García, J., Villafuerte-Fernandez, R., Ntezes-Hidalgo, P. I., Meade-Aguilar, J. A., Ramirez-GarciaLuna, J. L., and Martinez-Jimenez, M. A. (2021). Postoperative inguinal pain and disability after Lichtenstein versus ONSTEP hernia repair: analysis of responses to the inguinal pain questionnaire in Spanish. *Surgery Today*, 51(5):703–712.

[7] Alden, L. E., Buhr, K., Robichaud, M., Trew, J. L., and Plasencia, M. L. (2018). Treatment of social approach processes in adults with social anxiety disorder. *Journal of consulting and clinical psychology*, 86(6):505.

[8] Alhaddad, G., Danna, J., Younes-Harb, C., Velay, J.-L., and Longcamp, M. (2023). Effects of biscriptuality on graphomotor coordination dynamics. *Journal of Experimental Psychology: Human Perception and Performance*, 49(2):177–187. Place: Washington Publisher: American Psychological Association, American Psychological Association.

[9] Amiri, M., Rahmati, M., Hedayati, M., Nahidi, F., and Ramezani Tehrani, F. (2021). Effects of oral contraceptives on serum concentrations of adipokines and adiposity indices of women with polycystic ovary syndrome: a randomized controlled trial. *Journal of Endocrinological Investigation*, 44(3):567–580.

[10] Anderson, S. F. (2019). Best (but oft forgotten) practices: sample size planning for powerful studies. *American Journal of Clinical Nutrition*, 110(2):280–295. WOS:000478072300007.

[11] Aragão-Santos, J. C., Behm, D. G., de Moura, T. R., and Marzo Edir Da Silva-Grigoletto (2024). Dual-task training is as effective as functional training on the functional fitness of older women: a randomized clinical trial. *BMC Geriatrics*, 24:1–11. Place: London Publisher: BioMed Central.

[12] Ardizzi, M., Ferroni, F., Manini, A., Giudici, C., Maccaferri, E., Uccelli, S., and Umiltà, M. A. (2023). The influence of sensorimotor experience on beauty evaluation of preschool children. *Frontiers in Human Neuroscience*. Place: Lausanne Publisher: Frontiers Research Foundation.

[13] Armstrong, B., Trude, A. C. B., Johnson, C., Castelo, R. J., Zemanick, A., Haber-Sage, S., Arbaiza, R., and Black, M. M. (2019). CHAMP: A cluster randomized-control trial to prevent obesity in child care centers. *Contemporary Clinical Trials*, 86:105849.

[14] Armstrong, R. A. (2017). Recommendations for analysis of repeated-measures designs: testing and correcting for sphericity and use of manova and mixed model analysis. *Ophthalmic and Physiological Optics*, 37(5):585–593. WOS:000408313900005.

[15] Arrington, E. F., Alderson, R. M., Tarle, S. J., Roberts, D. K., Sarver, D. E., Sullivan, M. A., and Gette, J. A. (2022). Visuospatial working memory in attention-deficit/hyperactivity disorder: Characterizing path length and path crossings as mechanisms of impairment. *Neuropsychology*.

[16] Ashton-James, C. E., Doane, M., McNeilage, A. G., Gholamrezaei, A., Glare, P., and Finniss, D. (2024). Efficacy of an mHealth intervention to support pain self-management and improve analgesia in patients with rib fractures: protocol for a randomised controlled trial. *BMJ open*, 14(11):e086202.

[17] Athanasiou, L. V., Spanou, V. M., Katsogiannou, E. G., and Katsoulos, P. D. (2021). Hematological Features in Sheep with IgG and IgM Antibodies against Borrelia burgdorferi sensu lato. *Pathogens (Basel, Switzerland)*, 10(2).

[18] Attwood, S., Chesworth, S. J., and Parkin, B. L. (2020). Menu engineering to encourage sustainable food choices when dining out: An online trial of priced-based decoys. *Appetite*, 149:104601.

[19] Baba, Y., Ooyama, C., Tazawa, Y., and Kohzuki, M. (2021). Effects of Adachi Rehabilitation Programme on older adults under long-term care: A multi-centre controlled trial. *PLOS ONE*, 16(2):e0245646.

[20] Babik, I., Cunha, A. B., and Lobo, M. A. (2021). Assistive and Rehabilitative Effects of the Playskin LiftTM Exoskeletal Garment on Reaching and Object Exploration in Children With Arthrogryposis. *The American Journal of Occupational Therapy: Official Publication of the American Occupational Therapy Association*, 75(1):7501205110p1–7501205110p10.

[21] Babik, I., Cunha, A. B., Ross, S. M., Logan, S. W., Galloway, J. C., and Lobo, M. A. (2019). Means-end problem solving in infancy: Development, emergence of intentionality, and transfer of knowledge. *Developmental Psychobiology*, 61(2):191–202. WOS:000459130300003.

[22] Babik, I. and Lobo, M. A. (2023). Hand-Use Preferences for Reaching and Object Exploration in Children with Impaired Upper Extremity Functioning: The Role of Environmental Affordances. *Symmetry*, 15(12):2161. Place: Basel Publisher: MDPI AG.

[23] Backx, A. P. M., Spooren, A. I. F., Bongers-Janssen, H. M. H., and Bouwsema, H. (2018). Quality of life, burden and satisfaction with care in caregivers of patients with a spinal cord injury during and after rehabilitation. *Spinal Cord*, 56(9):890–899. WOS:000443983000008.

[24] Bagg, M. K., Lo, S., Cashin, A. G., Herbert, R. D., O'Connell, N. E., Lee, H., Hübscher, M., Wand, B. M., O'Hagan, E., Rizzo, R. R. N., Moseley, G. L., Stanton, T. R., Maher, C. G., Goodall, S., Saing, S., and McAuley, J. H. (2020). The RESOLVE Trial for people with chronic low back pain: statistical analysis plan. *Brazilian Journal of Physical Therapy*.

[25] Bagg, M. K., Wand, B. M., Cashin, A. G., Lee, H., Hübscher, M., Stanton, T. R., O'Connell, N. E., O'Hagan, E. T., Rizzo, R. R. N., Wewege, M. A., Rabey, M., Goodall, S., Saing, S., Lo, S. N., Luomajoki, H., Herbert, R. D., Maher, C. G., Moseley, G. L., and McAuley, J. H. (2022). Effect of Graded Sensorimotor Retraining on Pain Intensity in Patients With Chronic Low Back Pain: A Randomized Clinical Trial. *JAMA*, 328(5):430–439.

[26] Baghfalaki, T. (2019). Bayesian sample size determination for longitudinal studies with continuous response based on different scientific questions of interest. *Journal of Biopharmaceutical Statistics*, 29(2):244–270. WOS:000458877800002.

[27] Bais, B., Kamperman, A. M., Bijma, H. H., Hoogendijk, W. J., Souman, J. L., Knijff, E., and Lambregtse-van den Berg, M. P. (2020). Effects of bright light therapy for depression during pregnancy: a randomised, double-blind controlled trial. *BMJ open*, 10(10):e038030.

[28] Bais, B., Kamperman, A. M., van der Zwaag, M. D., Dieleman, G. C., Harmsen van der Vliet-Torij, H. W., Bijma, H. H., Lieverse, R., Hoogendijk, W. J. G., and Lambregtse-van den Berg, M. P. (2016). Bright light therapy in pregnant women with major depressive disorder: study protocol for a randomized, double-blind, controlled clinical trial. *BMC psychiatry*, 16(1):381.

[29] Bakker, M., Veldkamp, C. L. S., Olmo R van den Akker, Marcel A L M van Assen, Crompvoets, E., How Hwee Ong, and Wicherts, J. M. (2020). Recommendations in preregistrations and internal review board proposals promote formal power analyses but do not increase sample size. *PLOS ONE*, 15(7). Place: San Francisco Publisher: Public Library of Science.

[30] Baldi, D., Canullo, L., Pesce, P., Triestino, A., Gianfreda, F., De Angelis, N., Pistilli, V., Bagnasco, F., and Caponio, V. C. A. (2025). Case–Control Study with a 6-Month Follow-Up to Compare the Effect of Nano-Hydrophilic and Moderately Rough Implant Surfaces in Association with Transcrestal Sinus Lift. *Prosthesis*, 7(3):58. Number: 3 Publisher: Multidisciplinary Digital Publishing Institute.

[31] Bar-Oz, D., Hecht, I., Achiron, A., Midlij, M., Man, V., Bar Dayan, Y., and Burgansky-Eliash, Z. (2018). Glycemic control and quality of life following cataract surgery in patients

with type 2 diabetes and without maculopathy. *Current Eye Research*, 43(1):96–101. WOS:000427324100013.

[32] Baranger, D. A. A., Lindenmuth, M., Nance, M., Guyer, A. E., Keenan, K., Hipwell, A. E., Shaw, D. S., and Forbes, E. E. (2021). The longitudinal stability of fMRI activation during reward processing in adolescents and young adults. *NeuroImage*, 232:117872.

[33] Barreiro, A., Wolter-Warmerdam, K., Friedman, S., Hickey, F., Johnson, S., and Marmolejo, J. (2022). Child feeding practices in children with Down syndrome in relationship to ethnicity and BMI. *Appetite*, 170:105888.

[34] Bart, V. K. E., Sharavdorj, E., Boldbaatar, E., Bazarvaani, K., and Rieger, M. (2024). When time does not matter: cultures differ in their use of temporal cues to infer agency over action effects. *Psychological Research*, 88(3):815–825.

[35] Bates, A., Wells, M., Laven, R., Ferriman, L., Heiser, A., and Fitzpatrick, C. (2020). Effect of an injectable trace mineral supplement on the immune response of dairy calves. *Research in Veterinary Science*, 130:1–10.

[36] Bates, A., Wells, M., Laven, R. A., and Simpson, M. (2019). Reduction in morbidity and mortality of dairy calves from an injectable trace mineral supplement. *Veterinary Record*, 184(22). WOS:000471147200004.

[37] Baudelet, M., Duprez, F., Van den Steen, L., Nuyts, S., Nevens, D., Goeleven, A., Vandenbruaene, C., Massonet, H., Vergauwen, A., Bollen, H., Deschuymer, S., Wouters, K., Peeters, M., Van Laer, C., Mariën, S., Van den Brekel, M., van der Molen, L., Vauterin, T., van Dinther, J., Verstraete, H., Hutsebaut, I., Meersschout, S., Vanderveken, O., De Bodt, M., and Van Nuffelen, G. (2023). Increasing Adherence to Prophylactic Swallowing Exercises During Head and Neck Radiotherapy: The Multicenter, Randomized Controlled PRESTO-Trial. *Dysphagia*, 38(3):886–895.

[38] Becker, S., Gandhi, W., Chen, Y. J., and Schweinhardt, P. (2017). Subjective utility moderates bidirectional effects of conflicting motivations on pain perception. *Scientific Reports*, 7:7790. WOS:000407400500022.

[39] Bedwell, G. J., Louw, C., Parker, R., van den Broeke, E., Vlaeyen, J. W., Moseley, G. L., and Madden, V. J. (2022). The influence of a manipulation of threat on experimentally-induced secondary hyperalgesia. *PeerJ*, 10:e13512.

[40] Benier, K., Faulkner, N., Ladegaard, I., and Wickes, R. (2024). Reducing Islamophobia through Conversation: A Randomized Control Trial. *Social Psychology Quarterly*, 87(4):461–478. Publisher: SAGE Publications Inc.

[41] Bertozzi, F., Fischer, P. D., Aflatounian, F., Hutchison, K. A., Sforza, C., and Monfort, S. M. (2023). Influence of Fatigue on Cognitive-Motor Function During Unanticipated Landings. *The American Journal of Sports Medicine*, 51(10):2740–2747.

[42] Besser, L. M., Wiese, L., Cook, D. J., Holt, J., Magzamen, S., Minor, B., Mitsova, D., Park, J., Sablan, O., Tourelle, M., and Williams, C. (2025). Rural Roads to cognitive Resilience (RRR): A prospective cohort study protocol. *PLOS ONE*, 20(1):e0312660.

[43] Bey, K., Wolfsgruber, S., Karaca, I., Wagner, H., Lardenoije, R., Becker, J., Milz, E., Kornhuber, J., Peters, O., and Frölich, L. (2016). No association of the variant rs11887120 in DNMT3A with cognitive decline in individuals with mild cognitive impairment. *Epigenomics*, 8(5):593–598.

[44] Bijlstra, G., Holland, R. W., Dotsch, R., and Wigboldus, D. H. J. (2019). Stereotypes and prejudice affect the recognition of emotional body postures. *Emotion*, 19(2):189–199. Place: Washington Publisher: American Psychological Association, American Psychological Association.

[45] BinDhim, N. F., McGeechan, K., Alanazi, A. K. T., Alanazi, H. M. S., Alanazi, S. A. J., Al-Hadlaq, S. M., Aljadhey, H., Alhawassi, T. M., Alghamdi, N. A., Shaman, A. M., Alquwayzani, M. S., and Basyouni, M. H. (2018). Evaluating the pictorial warnings on tobacco products in Arabian Gulf countries against other international pictorial warnings. *Tobacco Control*, 27(3):261–265. WOS:000430496600013.

[46] Binyamin-Suissa, L., Hochman, S., and Henik, A. (2022). Asymmetric affective perspective taking effects toward valence influenced by personality perspective taken. *Psychonomic Bulletin & Review*.

[47] Blair, G., Cooper, J., Coppock, A., and Humphreys, M. (2019). Declaring and diagnosing research designs. *American Political Science Review*, 113(3):838–859.

[48] Bouzidi, Y. S. and Gendolla, G. H. E. (2023). Action-orientation shields against primed cognitive conflict effects on effort-related cardiac response. *Psychophysiology*, page e14407.

[49] Boyne, P., Billinger, S. A., Reisman, D. S., Awosika, O. O., Buckley, S., Burson, J., Carl, D., DeLange, M., Doren, S., Earnest, M., Gerson, M., Henry, M., Horning, A., Khoury, J. C., Kissela, B. M., Laughlin, A., McCartney, K., McQuaid, T., Miller, A., Moores, A., Palmer, J. A., Sucharew, H., Thompson, E. D., Wagner, E., Ward, J., Wasik, E. P., Whitaker, A. A., Wright, H., and Dunning, K. (2023). Optimal Intensity and Duration of Walking Rehabilitation in Patients With Chronic Stroke: A Randomized Clinical Trial. *JAMA neurology*, 80(4):342–351.

[50] Boyne, P., Meyrose, C., Westover, J., Whitesel, D., Hatter, K., Reisman, D. S., Carl, D., Khoury, J. C., Gerson, M., Kissela, B., and Dunning, K. (2020a). Effects of exercise intensity on acute circulating molecular responses poststroke. *Neurorehabilitation and Neural Repair*, page 1545968319899915. WOS:000509184100001.

[51] Boyne, P., Meyrose, C., Westover, J., Whitesel, D., Hatter, K., Reisman, D. S., Cunningham, D., Carl, D., Jansen, C., Khoury, J. C., Gerson, M., Kissela, B., and Dunning, K. (2019). Exercise intensity affects acute neurotrophic and neurophysiological responses poststroke. *Journal of Applied Physiology*, 126(2):431–443. WOS:000459820000018.

[52] Boyne, P., Scholl, V., Doren, S., Carl, D., Billinger, S. A., Reisman, D. S., Gerson, M., Kissela, B., Vannest, J., and Dunning, K. (2020b). Locomotor training intensity after stroke: Effects of interval type and mode. *Topics in Stroke Rehabilitation*, 27(7):483–493.

[53] Bromage, S., Tazhibayev, S., Zhou, X., Liu, C., Tserenkhuu, E., Dolmatova, O., Khishignemekh, M., Musurepova, L., Wusigale, Tsolmon, S., Tsendjav, E., Enkhmaa, D., Rai, R. K., Enkhbat, B., Menghe, B., and Ganmaa, D. (2025). Longitudinal analysis

of lifestyle risk factors, nutrition status and drivers of food choice among urban migrants in Ulaanbaatar, Mongolia, and Almaty, Kazakhstan: a formative study. *Public Health Nutrition*, 28(1):e33.

[54] Buscà, B., Aguilera-Castells, J., Arboix-Alió, J., Miró, A., Fort-Vanmeerhaeghe, A., Huertas, P., and Peña, J. (2022). Superimposed vibration on suspended push-ups. *PeerJ*, 10:e14435.

[55] Bustamante, L., Lieder, F., Musslick, S., Shenhav, A., and Cohen, J. (2021). Learning to Overexert Cognitive Control in a Stroop Task. *Cognitive, Affective & Behavioral Neuroscience.*

[56] Byrne, K. A., Roth, P. J., Merchant, K., Baginski, B., Robinson, K., Dumas, K., Collie, J., Ramsey, B., Cull, J., Cooper, L., Churitch, M., Rennert, L., Heo, M., and Jones, R. (2020). Inpatient link to peer recovery coaching: Results from a pilot randomized control trial. *Drug and Alcohol Dependence*, 215:108234. Place: Clare Publisher: Elsevier Ireland Ltd WOS:000573607200032.

[57] Cabral, D. A. R., Parma, J. O., Morris, D. L., and Miller, M. W. (2024). Conquering pressure! The effects of mild-anxiety training on motor performance under pressure during early motor learning. *Journal of Experimental Psychology: Human Perception and Performance*, 50(2):178–192. Place: Washington Publisher: American Psychological Association, American Psychological Association.

[58] Calbi, M., Langiulli, N., Ferroni, F., Montalti, M., Kolesnikov, A., Gallese, V., and Umiltà, M. A. (2021). The consequences of COVID-19 on social interactions: an online study on face covering. *Scientific Reports*, 11(1):2601.

[59] Calbi, M., Montalti, M., Pederzani, C., Arcuri, E., Umiltà, M. A., Gallese, V., and Mirabella, G. (2022). Emotional body postures affect inhibitory control only when task-relevant. *Frontiers in Psychology*, 13:1035328.

[60] Calvo-Malvar, M., Benítez-Estévez, A. J., Sánchez-Castro, J., Leis, R., and Gude, F. (2021). Effects of a Community-Based Behavioral Intervention with a Traditional Atlantic Diet on Cardiometabolic Risk Markers: A Cluster Randomized Controlled Trial ("The GALIAT Study"). *Nutrients*, 13(4):1211. Place: Basel Publisher: MDPI AG.

[61] Calvo-Malvar, M. D. M., Leis, R., Benítez-Estévez, A. J., Sánchez-Castro, J., and Gude, F. (2016). A randomised, family-focused dietary intervention to evaluate the Atlantic diet: the GALIAT study protocol. *BMC public health*, 16(1):820.

[62] Campbell, G., Mattick, R., Bruno, R., Larance, B., Nielsen, S., Cohen, M., Lintzeris, N., Shand, F., Hall, W. D., and Hoban, B. (2014). Cohort protocol paper: the pain and opioids in treatment (point) study. *BMC pharmacology and toxicology*, 15(1):17.

[63] Caruso, R., Nanni, M. G., Rodin, G., Hales, S., Malfitano, C., De Padova, S., Bertelli, T., Murri, M. B., Bovero, A., Miniotti, M., Leombruni, P., Zerbinati, L., Sabato, S., Grassi, L., and CALM-Italian Study (2020). Effectiveness of a brief manualized intervention, Managing Cancer and Living Meaningfully (CALM), adapted to the Italian cancer care setting: Study protocol for a single-blinded randomized controlled trial. *Contemporary Clinical Trials Communications*, 20:100661.

[64] Catháin, C. P. O. and Moran, K. A. (2025). Does accelerometer location influence recreational runners' response to an accelerometer-based biofeedback gait re-education system? *Journal of Sports Sciences*. Publisher: Routledge.

[65] Cavanna, D., Righetti, L., Elliott, C., and Suman, M. (2018). The scientific challenges in moving from targeted to non-targeted mass spectrometric methods for food fraud analysis: A proposed validation workflow to bring about a harmonized approach. *Trends in Food Science & Technology*, 80:223–241.

[66] Chacko, S. C., Quinzi, F., De Fano, A., Bianco, V., Mussini, E., Berchicci, M., Perri, R. L., and Di Russo, F. (2020). A single bout of vigorous-intensity aerobic exercise affects reactive, but not proactive cognitive brain functions. *International Journal of Psychophysiology: Official Journal of the International Organization of Psychophysiology*, 147:233–243.

[67] Chantziaras, I., Meyer, D., Vrielinck, L., Limbergen, T., Pineiro, C., Dewulf, J., Kyriazakis, I., and Maes, D. (2020). Environment-, health-, performance- and welfare-related parameters in pig barns with natural and mechanical ventilation. *Preventive veterinary medicine*, 183:105150.

[68] Chedid, C., Kokhreidze, E., Tukvadze, N., Banu, S., Uddin, M. K. M., Biswas, S., Russomando, G., Acosta, C. C. D., Arenas, R., Ranaivomanana, P. P., Razafimahatratra, C., Herindrainy, P., Rakotonirina, J., Raherinandrasana, A. H., Rakotosamimanana, N., Hamze, M., Ismail, M. B., Bayaa, R., Berland, J.-L., De Maio, F., Delogu, G., Endtz, H., Ader, F., Goletti, D., and Hoffmann, J. (2020). Relevance of QuantiFERON-TB Gold Plus and Heparin-Binding Hemagglutinin Interferon-γ Release Assays for Monitoring of Pulmonary Tuberculosis Clearance: A Multicentered Study. *Frontiers in Immunology*, 11:616450.

[69] Chen, J., Ke, Y., Ni, G., Liu, S., and Dong, M. (2024). Evidence for modulation of EEG microstates by mental workload levels and task types. *Human Brain Mapping*, 45(1). Place: San Antonio Publisher: John Wiley & Sons, Inc.

[70] Cheng, C., Tsai, J.-Y., Yang, Y. C. E., Esselman, R., Kalcic, M., Xu, X., and Mohai, P. (2017). Risk communication and climate justice planning: a case of michigan's huron river watershed. *Urban Planning*, 2(4):34–50. WOS:000419756200004.

[71] Chi, Y.-Y., Glueck, D. H., and Muller, K. E. (2018). Power and sample size for fixed-effects inference in reversible linear mixed models. *The American Statistician*, pages 1–10.

[72] Childs, D., Lee, N., Dewsnap, B., and Cadogan, J. (2019). A within-person theoretical perspective in sales research: outlining recommendations for adoption and consideration of boundary conditions. *Journal of Personal Selling & Sales Management*, 39:1–14.

[73] Christensen, R. A. G., Haykowsky, M. J., Nadler, M., Prado, C. M., Small, S. D., Rickard, J. N., Pituskin, E., Paterson, D. I., Mackey, J. R., Thompson, R. B., and Kirkham, A. A. (2023). Rationale and design of IMPACT-women: a randomised controlled trial of the effect of time-restricted eating, healthy eating and reduced sedentary behaviour on metabolic health during chemotherapy for early-stage breast cancer. *The British Journal of Nutrition*, 130(5):852–859.

[74] Chung, H.-F., Al Mamun, A., Huang, M.-C., Long, K. Z., Huang, Y.-F., Shin, S.-J., Hwang, S.-J., and Hsu, C.-C. (2017). Obesity, weight change, and chronic kidney disease in patients with type 2 diabetes mellitus: A longitudinal study in Taiwan. *Journal of Diabetes*, 9(11):983–993. WOS:000413303300003.

[75] Ciocca, L., Meneghello, R., Monaco, C., Savio, G., Scheda, L., Gatto, M. R., and Baldissara, P. (2018). In vitro assessment of the accuracy of digital impressions prepared using a single system for full-arch restorations on implants. *International journal of computer assisted radiology and surgery*, 13(7):1097–1108.

[76] Clarke, J. T. R., Coyle, D., Evans, G., Martin, J., and Winquist, E. (2014). Toward a functional definition of a "rare disease" for regulatory authorities and funding agencies. *Value in Health*, 17(8):757–761. WOS:000346918100001.

[77] Claudino, R. G. e., da Costa, I. F., Nascimento, I. N. d. A., and Torro, N. (2020). Use of sign language does not favor recognition of static and dynamic emotional faces in deaf people. *Psychology & Neuroscience*, 13(4):531–538. Place: US Publisher: Educational Publishing Foundation.

[78] Clayton, R. W., Thomas, C. H., Schaffer, B. S., Stratton, M., Garrison, E., and Mathews, L. G. (2017). Exercise and work-family conflict: a field experiment. *Journal of Managerial Psychology*, 32(3):225–238. WOS:000404815900002.

[79] Coderre, T. J. and Laferriere, A. (2020). The emergence of animal models of chronic pain and logistical and methodological issues concerning their use. *Journal of Neural Transmission*, 127(4):393–406. Place: Wien Publisher: Springer Wien WOS:000524953600002.

[80] Coffey, M. J., Nielsen, S., Wemheuer, B., Kaakoush, N. O., Garg, M., Needham, B., Pickford, R., Jaffe, A., Thomas, T., and Ooi, C. Y. (2019). Gut Microbiota in Children With Cystic Fibrosis: A Taxonomic and Functional Dysbiosis. *Scientific Reports*, 9(1):18593.

[81] Cole, J. H., Poudel, R. P. K., Tsagkrasoulis, D., Caan, M. W. A., Steves, C., Spector, T. D., and Montana, G. (2017). Predicting brain age with deep learning from raw imaging data results in a reliable and heritable biomarker. *Neuroimage*, 163:115–124. WOS:000418641800011.

[82] Colloca, L., Wang, Y., Martinez, P. E., Chang, Y.-P. C., Ryan, K. A., Hodgkinson, C., Goldman, D., and Dorsey, S. G. (2019). OPRM1 rs1799971, COMT rs4680, and FAAH rs324420 genes interact with placebo procedures to induce hypoalgesia. *Pain*, 160(8):1824–1834.

[83] Cometa, M. A., Lopez, B. M., Vasilopoulos, T., Destephens, A. J., Bigos, A., Lizdas, D. E., Gravenstein, N., and Lampotang, S. (2020). Does the Technique for Assessing Loss of Resistance Alter the Magnitude of Epidural Needle Tip Overshoot? *Simulation in Healthcare-Journal of the Society for Simulation in Healthcare*, 15(3):154–159. Place: Philadelphia Publisher: Lippincott Williams & Wilkins WOS:000549953800004.

[84] Cornelissen, K. K., Widdrington, H., McCarty, K., Pollet, T. V., Tovée, M. J., and Cornelissen, P. L. (2019). Are attitudinal and perceptual body image the same or different? Evidence from high-level adaptation. *Body Image*, 31:35–47.

[85] Cornelissen, P. L., Brokjøb, L. G., Gumančík, J., and Cornelissen, K. K. (2021). Women's self-estimates of body size are more accurate and precise when made with three-quarter view than front-view stimuli. *Body Image*, 38:171–180.

[86] Cornelissen, P. L., Cornelissen, K. K., Groves, V., McCarty, K., and Tovée, M. J. (2018). View-dependent accuracy in body mass judgements of female bodies. *Body image*, 24:116–123.

[87] Courcoulas, A. P., Stefater, M. A., Shirley, E., Gourash, W. F., and Stylopoulos, N. (2019). The feasibility of examining the effects of gastric bypass surgery on intestinal metabolism: prospective, longitudinal mechanistic clinical trial. *JMIR Research Protocols*, 8(1):e12459.

[88] Crameri, L., Hettiarachchi, I. T., and Hanoun, S. (2020). Effects of Dynamic Resilience on the Reactivity of Vagally Mediated Heart Rate Variability. *Frontiers in Psychology*, 11:579210.

[89] Crivelli, D., Crotti, D., Crottini, F., Peviani, V., Gandola, M., Bottini, G., and Salvato, G. (2023). Skin temperature changes in response to body ownership modulation vary according to the side of stimulation. *Physiology & Behavior*, 265:114142.

[90] Cunha, A. B., Babik, I., Ross, S. M., Logan, S. W., Galloway, J. C., Clary, E., and Lobo, M. A. (2018). Prematurity may negatively impact means-end problem solving across the first two years of life. *Research in Developmental Disabilities*, 81:24–36. WOS:000445443100003.

[91] Curtis, P. J., Berends, L., van der Velpen, V., Jennings, A., Haag, L., Chandra, P., Kay, C. D., Rimm, E. B., and Cassidy, A. (2022). Blueberry anthocyanin intake attenuates the postprandial cardiometabolic effect of an energy-dense food challenge: Results from a double blind, randomized controlled trial in metabolic syndrome participants. *Clinical Nutrition (Edinburgh, Scotland)*, 41(1):165–176.

[92] Curtis, P. J., van der Velpen, V., Berends, L., Jennings, A., Haag, L., Minihane, A.-M., Chandra, P., Kay, C. D., Rimm, E. B., and Cassidy, A. (2024). Chronic and postprandial effect of blueberries on cognitive function, alertness, and mood in participants with metabolic syndrome - results from a six-month, double-blind, randomized controlled trial. *The American Journal of Clinical Nutrition*, 119(3):658–668.

[93] Cysique, L. A., Jakabek, D., Bracken, S. G., Allen-Davidian, Y., Heng, B., Chow, S., Dehhaghi, M., Staats Pires, A., Darley, D. R., Byrne, A., Phetsouphanh, C., Kelleher, A., Dore, G. J., Matthews, G. V., Guillemin, G. J., and Brew, B. J. (2023). The kynurenine pathway relates to post-acute COVID-19 objective cognitive impairment and PASC. *Annals of Clinical and Translational Neurology.*

[94] Czakó, C., István, L., Benyó, F., Élő, A., Erdei, G., Horváth, H., Nagy, Z. Z., and Kovács, I. (2020). The impact of deterministic signal loss on oct angiography measurements. *Translational Vision Science & Technology*, 9(5):10–10. Publisher: The Association for Research in Vision and Ophthalmology.

[95] da Cunha Nascimento, D., Neto, I. V. d. S., Saraiva, B., Lima, A. d. S., Navalta, J. W., Pereira, G. B., Willardson, J. M., Rodrigues Beal, F. L., and Prestes, J. (2021).

Advancements and critical steps for statistical analyses in blood pressure response to resistance training in hypertensive older women: a methodological approach. *Blood Pressure Monitoring*, 26(2):135–145.

[96] da Silva, D. G., da Silva, R. F. B., Gantois, P., Nascimento, V. B., Nakamura, F. Y., and Fonseca, F. d. S. (2024). Accuracy and reliability of perception of bar velocity loss for autoregulation in resistance exercise. *International Journal of Sports Science & Coaching*, 19(4):1622–1631. Publisher: SAGE Publications.

[97] Daneva, Z., Ottolini, M., Chen, Y. L., Klimentova, E., Kuppusamy, M., Shah, S. A., Minshall, R. D., Seye, C. I., Laubach, V. E., Isakson, B. E., and Sonkusare, S. K. (2021). Endothelial pannexin 1-TRPV4 channel signaling lowers pulmonary arterial pressure in mice. *eLife*, 10:e67777.

[98] Davidson, L., Ellem, R., Keane, C., Chan, G., Broccatelli, C., Buckley, J., Walter, Z., Hallo, L., and Hides, L. (2022). A two-stage social network intervention for reducing alcohol and other drug use in residential colleges: Protocol for a feasibility trial. *Contemporary Clinical Trials*, 118:106779.

[99] Davis, C. G. and Goldfield, G. S. (2024). Limiting social media use decreases depression, anxiety, and fear of missing out in youth with emotional distress: A randomized controlled trial. *Psychology of popular media*. Publisher: Educational Publishing Foundation.

[100] de Andrade, D., Davidson, L., Robertson, C., Williams, P., Leung, J., Walter, Z., Allan, J., and Hides, L. (2024). Randomized effectiveness-implementation trial of dialectical behavior therapy interventions for young people with borderline personality disorder symptoms. *Journal of Clinical Psychology*, 80(10):2117–2133. _eprint: https://onlinelibrary.wiley.com/doi/pdf/10.1002/jclp.23725.

[101] de Castro Deus, M., Gadotti, A. C., Erika Sousa Dias, Júlia Bacarin Monte Alegre, Beatriz Akemi Kondo Van Spitzenbergen, Gabriela Bohnen Andrade, Sara Soares Tozoni, Stocco, R. B., Olandoski, M., Felipe Francisco Bondan Tuon, Pinho, R. A., de Noronha, L., Cristina Pellegrino Baena, and Moreno-Amaral, A. N. (2024). Prospective Variation of Cytokine Trends during COVID-19: A Progressive Approach from Disease Onset until Outcome. *International Journal of Molecular Sciences*, 25(19):10578. Place: Basel Publisher: MDPI AG.

[102] de Lima Alves da Silva, T. C., Hégila da Silva Dantas, Macedo, L. E., Talita Duarte Martins, Silva-Filho, E., Pegado, R., McLean, L., and Maria Thereza Albuquerque Barbosa Cabral Micussi (2024). Investigating the efficacy of transcranial direct current stimulation on chronic pain management in endometriosis patients: A randomized controlled trial protocol. *PLOS ONE*, 19(8). Place: San Francisco Publisher: Public Library of Science.

[103] de Oliveira, A. R., Reimer, A. E., Simandl, G. J., Nagrale, S. S., and Widge, A. S. (2021). Lost in translation: no effect of repeated optogenetic cortico-striatal stimulation on compulsivity in rats. *Translational Psychiatry*, 11(1):1–16. Number: 1 Publisher: Nature Publishing Group.

[104] Dean, C. J., Slizovskiy, I. B., Crone, K. K., Pfennig, A. X., Heins, B. J., Caixeta, L. S., and Noyes, N. R. (2020). Investigating the cow skin and teat canal microbiomes of

the bovine udder using different sampling and sequencing approaches. *Journal of Dairy Science*, 0(0). Publisher: Elsevier.

[105] Deere, R., Pallmann, P., Shepherd, V., Brookes-Howell, L., Carson-Stevens, A., Davies, F., Dunphy, E., Gupta, P., Hickson, M., Hill, V., Ingarfield, K., Ivins, N., Jones, F., Letchford, R., Lowe, R., Nash, S., Otter, P., Prout, H., Randell, E., Sewell, B., Smith, D., Trubey, R., Wainwright, T., Busse, M., and Button, K. (2024). MulTI-domain self-management in older People wiTh OstEoarthritis and multi-morbidities: protocol for the TIPTOE randomised controlled trial. *Trials*, 25(1):557.

[106] Deliali, K., Christofa, E., and Knodler, M. (2021). The role of protected intersections in improving bicycle safety and driver right-turning behavior. *Accident; Analysis and Prevention*, 159:106295.

[107] DeLozier, K. R., Gould, F. D. H., Ohlemacher, J., Thexton, A. J., and German, R. Z. (2018). Impact of recurrent laryngeal nerve lesion on oropharyngeal muscle activity and sensorimotor integration in an infant pig model. *Journal of Applied Physiology*, 125(1):159–166. WOS:000441191900016.

[108] Depp, C., Ehret, B., Villa, J., Perivoliotis, D., and Granholm, E. (2021). A Brief Mobile-Augmented Suicide Prevention Intervention for People With Psychotic Disorders in Transition From Acute to Ongoing Care: Protocol for a Pilot Trial. *JMIR research protocols*, 10(2):e14378.

[109] Derks, S., van Wijngaarden, S., Wouda, M., Schuengel, C., and Sterkenburg, P. S. (2019). Effectiveness of the serious game 'You & I' in changing mentalizing abilities of adults with mild to borderline intellectual disabilities: a parallel superiority randomized controlled trial. *Trials*, 20(1):500.

[110] Dewolf, A. H., Ivaniski-Mello, A., Peyré-Tartaruga, L. A., and Mesquita, R. M. (2024). Relation between soft tissue energy dissipation and leg stiffness in running at different step frequencies. *Royal Society Open Science*, 11(6):1–13. Place: London Publisher: The Royal Society Publishing.

[111] Dinler, C., Ulutas, B., Voyvoda, H., Ulutas, P. A., Ural, K., and Karagenc, T. (2017). Haptoglobin and serum amyloid-A concentrations and their relationship with oocyst count in neonatal lambs experimentally infected with Cryptosporidium parvum. *Veterinary Parasitology*, 247:49–56. WOS:000415774800009.

[112] Djordjevic, D., Cockalo, D., Bogetic, S., and Bakator, M. (2021). Predicting Entrepreneurial Intentions among the Youth in Serbia with a Classification Decision Tree Model with the QUEST Algorithm. *Mathematics*, 9(13):1487. Number: 13 Publisher: Multidisciplinary Digital Publishing Institute.

[113] Dobres, J., Wolfe, B., Chahine, N., and Reimer, B. (2018). The effects of visual crowding, text size, and positional uncertainty on text legibility at a glance. *Applied Ergonomics*, 70:240–246. WOS:000436224100028.

[114] Dolan, S. B., Wittenauer, R., Njoroge, A., Onyango, P., Owiso, G., Shearer, J. C., Lober, W. B., Liu, S., Puttkammer, N., and Rabinowitz, P. (2023). Time Utilization Among Immunization Clinics Using an Electronic Immunization Registry (Part 2): Time and Motion Study of Modified User Workflows. *JMIR formative research*, 7:e39777.

[115] Dos Santos Quaresma, M. V. L., Campos, R., Tavares-Silva, E., Marques, C. G., and Thomatieli-Santos, R. V. (2021). Effect of acute caffeine supplementation before intermittent high-intensity exercise on cytokine levels and psychobiological parameters: A randomized, cross-over, placebo-controlled trial. *Cytokine*, page 155583.

[116] Droitcourt, C., Barbarot, S., Maruani, A., Darrieux, L., Misery, L., Brenaut, E., Adamski, H., Chabbert, C., Vermersch, A., Weiborn, M., Seneschal, J., Taïeb, A., Plantin, P., Maillard, H., Phan, A., Skowron, F., Viguier, M., Staumont-Salle, D., Nosbaum, A., Soria, A., Barbaud, A., Oger, E., and Dupuy, A. (2019). A new phototherapy regimen during winter as an add-on therapy, coupled with oral vitamin D supplementation, for the long-term control of atopic dermatitis: study protocol for a multicentre, randomized, crossover, pragmatic trial – the PRADA trial. *Trials*, 20(1):184. Place: London Publisher: BioMed Central.

[117] Duarte, M., Pelorosso, F., Nicolosi, L. N., Salgado, M. V., Vetulli, H., Aquieri, A., Azzato, F., Castro, M., Coyle, J., Davolos, I., Criado, I. F., Gregori, R., Mastrodonato, P., Rubio, M. C., Sarquis, S., Wahlmann, F., and Rothlin, R. P. (2021). Telmisartan for treatment of Covid-19 patients: An open multicenter randomized clinical trial. *EClinicalMedicine*, 37:100962.

[118] Dunn, I., Power, E., Casey, L., and Wootton, B. (2025). Efficacy of videoconferencing-delivered cognitive behavioural therapy to reduce anxiety disorder severity in LGBTQ+ people: An exploratory trial protocol. *PLOS ONE*, 20.

[119] Dvorak, A. L. and Hernandez-Ruiz, E. (2019). Comparison of music stimuli to support mindfulness meditation. *Psychology of Music*, page UNSP 0305735619878497. WOS:000496378900001.

[120] Dziak, J. J., Dierker, L. C., and Abar, B. (2018). The interpretation of statistical power after the data have been gathered. *Current Psychology*.

[121] Eatherington, C. J., Mongillo, P., Lõoke, M., and Marinelli, L. (2021). Dogs fail to recognize a human pointing gesture in two-dimensional depictions of motion cues. *Behavioural Processes*, 189:104425.

[122] Eikelboom, W. S., Singleton, E., van den Berg, E., Coesmans, M., Mattace Raso, F., van Bruchem, R. L., Goudzwaard, J. A., de Jong, F. J., Koopmanschap, M., den Heijer, T., Driesen, J. J. M., Vroegindeweij, L. J. H. M., Thomeer, E. C., Hoogers, S. E., Dijkstra, A. A., Zuidema, S. U., Pijnenburg, Y. A. L., Scheltens, P., van Swieten, J. C., Ossenkoppele, R., and Papma, J. M. (2019). Early recognition and treatment of neuropsychiatric symptoms to improve quality of life in early Alzheimer's disease: protocol of the BEAT-IT study. *Alzheimer's Research & Therapy*, 11(1):48.

[123] Elhakeem, A., Hughes, R. A., Tilling, K., Cousminer, D. L., Jackowski, S. A., Cole, T. J., Kwong, A. S. F., Li, Z., Grant, S. F. A., Baxter-Jones, A. D. G., Zemel, B. S., and Lawlor, D. A. (2022). Using linear and natural cubic splines, SITAR, and latent trajectory models to characterise nonlinear longitudinal growth trajectories in cohort studies. *BMC Medical Research Methodology*, 22(1):68.

[124] Elheeny, A. A. H. and Abdelmotelb, M. A. (2022). Oral health–related quality of life (OHRQOL) of preschool children's anterior teeth restored with zirconia crowns versus

resin-bonded composite strip crowns: a 12-month prospective clinical trial. *Clinical Oral Investigations.*

[125] Erbes, V. and Spors, S. (2020). Localisation Properties of Wave Field Synthesis in a Listening Room. *IEEE-ACM Transactions on Audio Speech and Language Processing*, 28:1016–1024. Place: Piscataway Publisher: Ieee-Inst Electrical Electronics Engineers Inc WOS:000528052500001.

[126] Esfandiari, E., Kamyab, M., Yazdi, H. R., Sanjari, M. A., and Navvab Motlagh, F. (2019). The effect of a lateral wedge insole and a subtalar strap on gait parameters in knee osteoarthritis. *Medical Journal of the Islamic Republic of Iran*, 33:157.

[127] Evans, D., van Rensburg, C., Govathson, C., Ivanova, O., Rieß, F., Siroka, A., Sillah, A. K., Ntinginya, N. E., Jani, I., Sathar, F., Rosen, S., Sanne, I., Rachow, A., and Lönnroth, K. (2021). Adaptation of WHO's generic tuberculosis patient cost instrument for a longitudinal study in Africa. *Global Health Action*, 14(1):1865625.

[128] Ewen, V., Mushquash, A. R., Mushquash, C. J., Bailey, S. K., Haggarty, J. M., and Stones, M. J. (2018). Single-session therapy in outpatient mental health services: Examining the effect on mental health symptoms and functioning. *Social Work in Mental Health*, 16(5):573–589. Publisher: Routledge _eprint: https://doi.org/10.1080/15332985.2018.1456503.

[129] Eyowas, F. A., Schneider, M., Alemu, S., and Getahun, F. A. (2021). Multimorbidity of chronic non-communicable diseases: burden, care provision and outcomes over time among patients attending chronic outpatient medical care in Bahir Dar, Ethiopia-a mixed methods study protocol. *BMJ open*, 11(9):e051107.

[130] Eyowas, F. A., Schneider, M., Alemu, S., Pati, S., and Getahun, F. A. (2022). Magnitude, pattern and correlates of multimorbidity among patients attending chronic outpatient medical care in Bahir Dar, northwest Ethiopia: The application of latent class analysis model. *PLOS ONE*, 17(4). Place: San Francisco Publisher: Public Library of Science.

[131] Faisal, M., Rusetskaya, A., Väli, L., Taba, P., Minajeva, A., and Hickey, M. A. (2024). No Evidence of Sensory Neuropathy in a Traditional Mouse Model of Idiopathic Parkinson's Disease. *Cells*, 13(10):799. Place: Basel Publisher: MDPI AG.

[132] Fandiño-Losada, A. (2019). Clinical differences between children with asthma and rhinitis in rural and urban areas. *Colombia Medica (Cali, Colombia)*, 50(1):46–51.

[133] Faulkner, N., Wright, B., Lennox, A., Bismark, M., Boag, J., Boffa, S., Waxman, B., Watson-Kruse, J., Paine, G., and Bragge, P. (2020). Simulation-based training for increasing health service board members' effectiveness: a cluster randomised controlled trial. *BMJ Open*, 10(12). Place: London Publisher: BMJ Publishing Group LTD.

[134] Fiorillo, D., McLean, C., Pistorello, J., Hayes, S. C., and Follette, V. M. (2017). Evaluation of a web-based acceptance and commitment therapy program for women with trauma-related problems: A pilot study. *Journal of Contextual Behavioral Science*, 6(1):104–113. WOS:000404437400015.

[135] Fischer, P. D., Hutchison, K. A., Becker, J. N., and Monfort, S. M. (2021). Evaluating the Spectrum of Cognitive-Motor Relationships During Dual-Task Jump Landing. *Journal of Applied Biomechanics*, pages 1–8.

[136] Fornaro, S., Visalli, A., Viviani, G., Ambrosini, E., and Vallesi, A. (2024). Proactive control for conflict resolution is intact in subclinical obsessive-compulsive individuals. *Frontiers in Psychology*, 15:1490147.

[137] Fortin, A., Rabasa-Lhoret, R., Lemieux, S., Labonté, M.-E., and Gingras, V. (2018). Comparison of a Mediterranean to a low-fat diet intervention in adults with type 1 diabetes and metabolic syndrome: A 6–month randomized trial. *Nutrition, Metabolism and Cardiovascular Diseases*, 28(12):1275–1284.

[138] French, M. (2024). Effects of low intensity repetitive transcranial stimulation (rTMS) in humans and a review of glial responses to rTMS. Master's thesis, The University of Western Australia.

[139] Fuglestad, P. T., Rothman, A. J., and Linde, J. A. (2023). Applying Regulatory Focus Theory to Encourage Weight Loss in a Self-directed Intervention. *International Journal of Behavioral Medicine*.

[140] Fujiyama, H., Tan, J., Puri, R., and Hinder, M. R. (2022). Influence of tDCS over right inferior frontal gyrus and pre-supplementary motor area on perceptual decision-making and response inhibition: A healthy ageing perspective. *Neurobiology of Aging*, 109:11–21.

[141] Gaewkhiew, P., Sabbah, W., and Bernabé, E. (2020). Functional dentition and changes in dietary patterns among older adults in Thailand. *Public Health Nutrition*, pages 1–8. Publisher: Cambridge University Press.

[142] Gao, J. and Zhang, Y. (2024). Matching emotion regulation strategies with specific emotions in tourist experiences. *Journal of Hospitality and Tourism Insights*, ahead-of-print(ahead-of-print). Publisher: Emerald Publishing Limited.

[143] Garcia, A. F., Wilborn, K., and Mangold, D. L. (2017). The cortisol awakening response mediates the relationship between acculturative stress and self-reported health in Mexican Americans. *Annals of Behavioral Medicine*, 51(6):787–798.

[144] Gardier, M. and Geurten, M. (2025). Is Uncertainty in the Eyes or in Parents' Talk? Linking an Eye-Tracking Measure of Toddlers' Core Metacognition to Parental Metacognitive Talk. *Child Development*, n/a(n/a). _eprint: https://onlinelibrary.wiley.com/doi/pdf/10.1111/cdev.14237.

[145] Garrett-Ruffin, S., Hindash, A. C., Kaczkurkin, A. N., Mears, R. P., Morales, S., Paul, K., Pavlov, Y. G., and Keil, A. (2021). Open science in psychophysiology: An overview of challenges and emerging solutions. *International Journal of Psychophysiology: Official Journal of the International Organization of Psychophysiology*, 162:69–78.

[146] Gasparinho, C., Kanjungo, A., Zage, F., Clemente, I., Santos-Reis, A., Brito, M., Sousa-Figueiredo, J. C., Fortes, F., and Gonçalves, L. (2021). Impact of Annual Albendazole versus Four-Monthly Test-and-Treat Approach of Intestinal Parasites on Children Growth-A Longitudinal Four-Arm Randomized Parallel Trial during Two Years of a Community Follow-Up in Bengo, Angola. *Pathogens (Basel, Switzerland)*, 10(3).

[147] Geed, S., Lane, C. J., Nelsen, M. A., Wolf, S. L., Winstein, C. J., and Dromerick, A. W. (2021). Inaccurate Use of the Upper Extremity Fugl-Meyer Negatively Affects Upper Extremity Rehabilitation Trial Design: Findings From the ICARE Randomized Controlled Trial. *Archives of Physical Medicine and Rehabilitation*, 102(2):270–279.

[148] Geraets, C. N. W., van Beilen, M., Pot-Kolder, R., Counotte, J., van der Gaag, M., and Veling, W. (2018). Social environments and interpersonal distance regulation in psychosis: A virtual reality study. *Schizophrenia Research*, 192:96–101. WOS:000426344800015.

[149] Geraldi, M. V., Cazarin, C. B. B., Cristianini, M., Vasques, A. C. J., Geloneze, B., and Júnior, M. R. M. (2021). Jabuticaba juice improves postprandial glucagon-like peptide-1 and antioxidant status in healthy adults: a randomised crossover trial. *British Journal of Nutrition*, pages 1–10. Publisher: Cambridge University Press.

[150] Gerber, G. K. (2014). The dynamic microbiome. *FEBS Letters*, 588(22):4131–4139. WOS:000344076600005.

[151] Gholamrezaei, A., Diest, I. V., Aziz, Q., Vlaeyen, J. W. S., and Oudenhove, L. V. (2021). Controlled breathing and pain: Respiratory rate and inspiratory loading modulate cardiovascular autonomic responses, but not pain. *Psychophysiology*, n/a(n/a):e13895. _eprint: https://onlinelibrary.wiley.com/doi/pdf/10.1111/psyp.13895.

[152] Ghosh, A., Highton, D., Kolyva, C., Tachtsidis, I., Elwell, C. E., and Smith, M. (2017). Hyperoxia results in increased aerobic metabolism following acute brain injury. *Journal of Cerebral Blood Flow and Metabolism*, 37(8):2910–2920. WOS:000406514300021.

[153] Giger, J. T., Pope, N. D., Vogt, H. B., Gutierrez, C., Newland, L. A., Lemke, J., and Lawler, M. J. (2015). Remote patient monitoring acceptance trends among older adults residing in a frontier state. *Computers in Human Behavior*, 44:174–182. WOS:000348965000019.

[154] Gil-Gomez de Liano, B., Stablum, F., and Umilta, C. (2016). Can concurrent memory load reduce distraction? A replication study and beyond. *Journal of Experimental Psychology-General*, 145(1):E1–E12. WOS:000367448400001.

[155] Gillanders, D., Ferreira, N. B., Angioni, E., Carvalho, S. A., and Eugenicos, M. P. (2017). An implementation trial of ACT-based bibliotherapy for irritable bowel syndrome. *Journal of Contextual Behavioral Science*, 6(2):172–177. WOS:000404437600007.

[156] Goebel, J. R., Ferolito, M., and Gorman, N. (2019). Pain Screening in the Older Adult With Delirium. *Pain Management Nursing*.

[157] Goldstein, S. P., Evans, B. C., Flack, D., Juarascio, A., Manasse, S., Zhang, F., and Forman, E. M. (2017). Return of the jitai: applying a just-in-time adaptive intervention framework to the development of m-health solutions for addictive behaviors. *International Journal of Behavioral Medicine*, 24(5):673–682. WOS:000411642000005.

[158] González-Salvado, V., Abelairas-Gómez, C., Gude, F., Peña-Gil, C., Neiro-Rey, C., González-Juanatey, J. R., and Rodríguez-Núñez, A. (2019). Targeting relatives: Impact of a cardiac rehabilitation programme including basic life support training on their skills and attitudes. *European journal of preventive cardiology*, page 2047487319830190.

[159] Gribbin, M. J., Chi, Y.-Y., Stewart, P. W., and Muller, K. E. (2013). Confidence regions for repeated measures ANOVA power curves based on estimated covariance. *BMC Medical Research Methodology*, 13(1):57.

[160] Grøvle, L., Hasvik, E., Holst, R., and Haugen, A. J. (2022). NSAIDs in sciatica (NIS): study protocol for an investigator-initiated multicentre, randomized placebo-controlled trial of naproxen in patients with sciatica. *Trials*, 23(1):493.

[161] Guay, C. S., Kafashan, M., Huels, E. R., Jiang, Y., Beyoglu, B., Spencer, J. W., Geczi, K., Apakama, G., Ju, Y.-E. S., Wildes, T. S., Avidan, M. S., and Palanca, B. J. A. (2022). Postoperative Delirium Severity and Recovery Correlate With Electroencephalogram Spectral Features. *Anesthesia and Analgesia*.

[162] Guertler, D., Krause, K., Moehring, A., Bischof, G., Batra, A., Freyer-Adam, J., Ulbricht, S., Rumpf, H. J., Wurm, S., Cuijpers, P., Lucht, M., John, U., and Meyer, C. (2023). E-Health intervention for subthreshold depression: Reach and two-year effects of a randomized controlled trial. *Journal of Affective Disorders*, 339:33–42.

[163] Guo, J.-W., Sward, K., Beck, S., Wong, B., Staggers, N., and Frey, L. (2014). Using a content analysis to identify study eligibility criteria concepts in cancer nursing research. *CIN: Computers, Informatics, Nursing*, 32(7):333–342. WOS:000348186500005.

[164] Guo, Y., Logan, H. L., Glueck, D. H., and Muller, K. E. (2013). Selecting a sample size for studies with repeated measures. *BMC Medical Research Methodology*, 13:100. Place: London Publisher: BioMed Central.

[165] Guo, Y. and Pandis, N. (2015). Sample-size calculation for repeated-measures and longitudinal studies. *American Journal of Orthodontics and Dentofacial Orthopedics*, 147(1):146–149.

[166] Gutierrez, D., Dorais, S., and Niles, J. (2025). Spiritual competency training in mental health and multicultural orientation and contemplative awareness: An evaluation of two training approaches with psychotherapists. *Psychotherapy*. Place: US Publisher: Educational Publishing Foundation.

[167] Gutiérrez, N., Jiménez, J. E., and de León, S. C. (2021). Reading Curriculum-Based Measures for Universal Screening in Monolingual Spanish First Graders. *Early Education and Development*, 0(0):1–25. Publisher: Routledge _eprint: https://doi.org/10.1080/10409289.2021.1935537.

[168] Hajna, S., Ross, N. A., Joseph, L., Harper, S., and Dasgupta, K. (2016). Neighbourhood walkability and daily steps in adults with type 2 diabetes. *PLOS ONE*, 11(3):e0151544. WOS:000372582800073.

[169] Halverson, T. F., Meyer-Kalos, P. S., Perkins, D. O., Gaylord, S. A., Palsson, O. S., Nye, L., Algoe, S. B., Grewen, K., and Penn, D. L. (2021). Enhancing stress reactivity and wellbeing in early schizophrenia: A randomized controlled trial of Integrated Coping Awareness Therapy (I-CAT). *Schizophrenia Research*, 235:91–101.

[170] Hamid, Z., Basit, A., Pontis, S., Piras, F., Assogna, F., Bossù, P., Pontieri, F. E., Stefani, A., Spalletta, G., Franceschi, P., Reggiani, A., and Armirotti, A. (2019). Gender specific decrease of a set of circulating N-acylphosphatidyl ethanolamines (NAPEs) in the plasma of Parkinson's disease patients. *Metabolomics*, 15(5).

[171] Hamilton, L. and Allard, E. (2019). Words matter: age-related positivity in episodic memory for abstract but not concrete words. *Aging, Neuropsychology, and Cognition*, 27:1–22.

[172] Haponenko, H., Britt, N., Cochrane, B., and Sun, H.-J. (2024). Inhibition of return in a 3D scene depends on the direction of depth switch between cue and target. *Attention, Perception & Psychophysics*, 86(8):2624–2642.

[173] Hardcastle, C., Hausman, H. K., Kraft, J. N., Albizu, A., O'Shea, A., Boutzoukas, E. M., Evangelista, N. D., Langer, K., Van Etten, E. J., Bharadwaj, P. K., Song, H., Smith, S. G., Porges, E., DeKosky, S. T., Hishaw, G. A., Wu, S. S., Marsiske, M., Cohen, R., Alexander, G. E., and Woods, A. J. (2022). Proximal improvement and higher-order resting state network change after multidomain cognitive training intervention in healthy older adults. *GeroScience*.

[174] Harnas, S. J., Knoop, H., Bennebroek Evertsz, F., Booij, S. H., Dekker, J., van Laarhoven, H. W. M., van der Lee, M., Meijer, E., Sharpe, L., Sprangers, M. A. G., van Straten, A., Zweegman, S., and Braamse, A. M. J. (2021). Personalized versus standard cognitive behavioral therapy for fear of cancer recurrence, depressive symptoms or cancer-related fatigue in cancer survivors: study protocol of a randomized controlled trial (MATCH-study). *Trials*, 22(1):696.

[175] Harrall, K. K., Muller, K. E., Starling, A. P., Dabelea, D., Barton, K. E., Adgate, J. L., and Glueck, D. H. (2023). Power and sample size analysis for longitudinal mixed models of health in populations exposed to environmental contaminants: a tutorial. *BMC Medical Research Methodology*, 23(1):12.

[176] Harrall, K. K., Sauder, K. A., Glueck, D. H., Shenkman, E. A., and Muller, K. E. (2024). Using Power Analysis to Choose the Unit of Randomization, Outcome, and Approach for Subgroup Analysis for a Multilevel Randomized Controlled Clinical Trial to Reduce Disparities in Cardiovascular Health. *Prevention Science, suppl. 3*, 25:433–445. Place: New York Publisher: Springer Nature B.V.

[177] Harvey, A. G., Dong, L., Lee, J. Y., Gumport, N. B., Hollon, S. D., Rabe-Hesketh, S., Hein, K., Haman, K., McNamara, M. E., and Weaver, C. (2017). Can integrating the Memory Support Intervention into cognitive therapy improve depression outcome? Study protocol for a randomized controlled trial. *Trials*, 18(1):539.

[178] Haughey, J. P. and Fine, P. (2020). Effects of the lower jaw position on athletic performance of elite athletes. *BMJ Open Sport & Exercise Medicine*, 6(1):e000886. Publisher: BMJ Specialist Journals Section: Short report.

[179] He, C., Liu, R., Gao, Z., Zhao, X., Sims, C. A., and Nayga, R. M. (2021). Does local label bias consumer taste buds and preference? Evidence of a strawberry sensory experiment. *Agribusiness*, 37(3):550–568. _eprint: https://onlinelibrary.wiley.com/doi/pdf/10.1002/agr.21680.

[180] Healy, G. N., Goode, A. D., Abbott, A., Burzic, J., Clark, B. K., Dunstan, D. W., Eakin, E. G., Frith, M., Gilson, N. D., Gao, L., Gunning, L., Jetann, J., LaMontagne, A. D., Lawler, S. P., Moodie, M., Nguyen, P., Owen, N., Straker, L., Timmins, P., Ulyate, L., and Winkler, E. A. H. (2020). Supporting Workers to Sit Less and Move More Through

the Web-Based BeUpstanding Program: Protocol for a Single-Arm, Repeated Measures Implementation Study. *JMIR Research Protocols*, 9(5). Place: Toronto Publisher: JMIR Publications.

[181] Hecht, I., Achiron, A., Bartov, E., Maharshak, I., Mendel, L., Pe'er, L., Bar, A., and Burgansky-Eliash, Z. (2019). Effects of dietary and lifestyle recommendations on patients with glaucoma: A randomized controlled pilot trial. *European Journal of Integrative Medicine*, 25:60–66. WOS:000456724400010.

[182] Hecht, I., Yeshurun, I., Bartov, E., Bar, A., Burgansky-Eliash, Z., and Achiron, A. (2018). Retinal layers thickness changes following epiretinal membrane surgery. *Eye*, 32(3):555.

[183] Hedrick, T. L., Harrigan, A. M., Thiele, R. H., Friel, C. M., Kozower, B. D., and Stukenborg, G. J. (2017). A pilot study of patient-centered outcome assessment using PROMIS for patients undergoing colorectal surgery. *Supportive Care in Cancer*, 25(10):3103–3112. WOS:000408727600016.

[184] Heffernan, M., Andrews, G., Fiatarone Singh, M. A., Valenzuela, M., Anstey, K. J., Maeder, A., McNeil, J., Jorm, L., Lautenschlager, N., and Sachdev, P. (2019). Maintain your brain: protocol of a 3-year randomized controlled trial of a personalized multi-modal digital health intervention to prevent cognitive decline among community dwelling 55 to 77 year olds. *Journal of Alzheimer's Disease*, 70:1–17.

[185] Helminen, E. C. and Scheer, J. R. (2023). Cardiovascular, self-report, and behavioral stress reactivity to the group-based Trier Social Stress Test with pandemic-related protocol adaptations. *International Journal of Psychophysiology: Official Journal of the International Organization of Psychophysiology*, 188:17–23.

[186] Hengartner, M. P. and Yamanaka-Altenstein, M. (2017). Personality, psychopathology, and psychotherapy: a pre-specified analysis protocol for confirmatory research on personality-psychopathology associations in psychotherapy outpatients. *Frontiers in Psychiatry*, 8:9. WOS:000393071200001.

[187] Hernandez-Ruiz, E., Dvorak, A. L., and Weingarten, K. (2020). Music stimuli in mindfulness meditation: Comparison of musician and non-musician responses. *Psychology of Music*, page 0305735620901338. Publisher: SAGE Publications Ltd.

[188] Hessler, J. B., Fischer, A. M., and Jahn, T. (2016). Differential linguistic recall effects in the california verbal learning test in healthy aging and alzheimer's dementia: analysis of routine diagnostic data. *Archives of Clinical Neuropsychology*, 31(7):689–699. WOS:000387965300002.

[189] Hey, M., DiBiase, E., Roach, D., Carr, D., and Haynes, K. (2020). Interactions between artificial light at night, soil moisture, and plant density affect the growth of a perennial wildflower. *Oecologia*, 193.

[190] Hilchey, M. D., Pratt, J., and Lamy, D. (2019). Is attention really biased toward the last target location in visual search? The role of focal attention and stimulus-response translation rules. *Journal of Experimental Psychology: Human Perception and Performance*, 45(10):1415–1428. Place: Washington Publisher: American Psychological Association, American Psychological Association.

[191] Hilchey, M. D., Rajsic, J., and Pratt, J. (2020). When do response-related episodic retrieval effects co-occur with inhibition of return? *Attention, Perception, & Psychophysics.*

[192] Hildyard, J. C. W., Riddell, D. O., Harron, R. C. M., Rawson, F., Foster, E. M. A., Massey, C., Taylor-Brown, F., Wells, D. J., and Piercy, R. J. (2022). The skeletal muscle phenotype of the DE50-MD dog model of Duchenne muscular dystrophy. *Wellcome Open Research*, 7:238.

[193] Hoben, M., Norton, P. G., Ginsburg, L. R., Anderson, R. A., Cummings, G. G., Lanham, H. J., Squires, J. E., Taylor, D., Wagg, A. S., and Estabrooks, C. A. (2017). Improving nursing home care through feedback on performance data (inform): protocol for a cluster-randomized trial. *Trials*, 18:9. WOS:000391413100002.

[194] Huang, S.-L., Li, R.-H., Huang, F.-Y., and Tang, F.-C. (2015). The potential for mindfulness-based intervention in workplace mental health promotion: results of a randomized controlled trial. *PLOS ONE*, 10(9):e0138089. WOS:000361601100221.

[195] Huffman, G., Hilchey, M. D., Weidler, B. J., Mills, M., and Pratt, J. (2020). Does feature-based attention play a role in the episodic retrieval of event files? *Journal of Experimental Psychology-Human Perception and Performance*, 46(3):241–251. Place: Washington Publisher: Amer Psychological Assoc WOS:000516585000002.

[196] Hum, J., Rietveld, T., Wiedijka, P., and van Lieshout, P. (2017). A pilot study into a possible relationship between diet and stuttering. *Journal of Fluency Disorders*, 52:25–36. WOS:000404316800003.

[197] Hung, S., Serwa, K., Rosenthal, G., and Eng, J. (2025). Validity of heart rate measurements in wrist-based monitors across skin tones during exercise. *PLOS ONE*, 20.

[198] Hunt, B. W. and De Pascalis, L. (2024). In the eye and mind of the beholder: The effects of familiarisation on the perception of atypical infant facial configurations. *PLOS ONE*, 19(11). Place: San Francisco Publisher: Public Library of Science.

[199] Hunt, B. W., Rayson, H., Bannard, C., and De Pascalis, L. (2023). In the mind of the beholder: The effects of familiarisation on the perception of atypical infant facial configurations. *PLOS ONE*, 18(7). Place: San Francisco Publisher: Public Library of Science.

[200] Ibrahim, F. and Stribling, P. (2019). A 5ad dietary protocol for functional bowel disorders. *Nutrients*, 11(8).

[201] Ilic, N. V., Dubljanin-Raspopovic, E., Nedeljkovic, U., Tomanovic-Vujadinovic, S., Milanovic, S. D., Petronic-Markovic, I., and Ilic, T. V. (2016). Effects of anodal tDCS and occupational therapy on fine motor skill deficits in patients with chronic stroke. *Restorative Neurology and Neuroscience*, 34(6):935–945. WOS:000389243100006.

[202] Iram, N., Saeed, B. Z., and Parveen, S. (2022). Ambulatory Hysteroscopy in Abnormal Uterine Bleeding: A Tertiary Care Hospital Perspective. *Journal of Shalamar Medical & Dental College - JSHMDC*, 3(2):167–172. Number: 2.

[203] Ishikura, I. A., Lucena, L., Andersen, M. L., Tufik, S., and Hachul, H. (2023). The relation of insomnia and obstructive sleep apnea on sexual function and climacteric symptoms in postmenopausal women. *Sleep Epidemiology*, 3:100063.

[204] Ito, K. and Ong, C. W. (2023). Perception of emotional tears with body postures, visual scenes, and written scenarios. *Asian Journal of Social Psychology*, 26(1):52–68. _eprint: https://onlinelibrary.wiley.com/doi/pdf/10.1111/ajsp.12544.

[205] Iven, J., Biesiekierski, J. R., Zhao, D., Deloose, E., O'Daly, O. G., Depoortere, I., Tack, J., and Van Oudenhove, L. (2018). Intragastric quinine administration decreases hedonic eating in healthy women through peptide-mediated gut-brain signaling mechanisms. *Nutritional neuroscience*, pages 1–13.

[206] Jacobson, N., Lithgow, B., Jozani, M. J., and Moussavi, Z. (2022). The Effect of Transcranial Alternating Current Stimulation With Cognitive Training on Executive Brain Function in Individuals With Dementia: Protocol for a Crossover Randomized Controlled Trial. *JMIR Research Protocols*, 11(4):e37282. Company: JMIR Research Protocols Distributor: JMIR Research Protocols Institution: JMIR Research Protocols Label: JMIR Research Protocols Publisher: JMIR Publications Inc., Toronto, Canada.

[207] Janke, E. A., Fritz, M., Hopkins, C., Haltzman, B., Sautter, J. M., and Ramirez, M. L. (2014). A randomized clinical trial of an integrated behavioral self-management intervention Simultaneously Targeting Obesity and Pain: the STOP trial. *BMC public health*, 14(1):621.

[208] Jansson-Fröjmark, M. and Sunnhed, R. (2024). Smartphone application-delivered cognitive behavioural therapy for insomnia with telephone support for insomnia disorder compared to a waitlist control: a randomised clinical trial. *Journal of Sleep Research*, page e14363.

[209] Jibb, L. A., Nanos, S. M., Alexander, S., Malfitano, C., Rydall, A., Gupta, S., Schimmer, A. D., Zimmermann, C., Hales, S., Nissim, R., Marmar, C., Schultebraucks, K., Mah, K., and Rodin, G. (2022). Traumatic stress symptoms in family caregivers of patients with acute leukaemia: protocol for a multisite mixed methods, longitudinal, observational study. *BMJ Open*, 12(11):e065422. Publisher: British Medical Journal Publishing Group Section: Oncology.

[210] Joa, C. Y. and Magsamen-Conrad, K. (2021). Social influence and UTAUT in predicting digital immigrants' technology use. *Behaviour & Information Technology*, 0(0):1–19. Publisher: Taylor & Francis _eprint: https://doi.org/10.1080/0144929X.2021.1892192.

[211] Jones, J. S., Milton, F., Mostazir, M., and Adlam, A. R. (2019a). The academic outcomes of working memory and metacognitive strategy training in children: A double-blind randomized controlled trial. *Developmental Science*, n/a(n/a):e12870.

[212] Jones, T. C., Steel, B. C., and Scott-Smith, T. (2019b). Description benefits, production benefits, and context retrieval for recognition of unfamiliar faces. *American Journal of Psychology*, 132(4):397–419. WOS:000510024600001.

[213] Jung, W.-H., Takeuchi, R., Chun, C.-W., Lee, J.-S., Ha, J.-H., Kim, J.-H., and Jeong, J.-H. (2014). Efficacy of periarticular multimodal drug injection after medial opening-wedge high tibial osteotomy: a randomized, controlled study. *Arthroscopy-the Journal of Arthroscopic and Related Surgery*, 30(10):1261–1268. WOS:000343149500014.

[214] Jurkowski, S., Mundelsee, L., and Hänze, M. (2024). Strengthening collaborative learning in secondary school: Development and evaluation of a lesson-integrated training approach for transactive communication. *Learning and Instruction*, 92:101934.

[215] Kalweit, K. L., van Zyl, D. G., and Rheeder, P. (2018). Titrating insulin in patients with type 2 diabetes using a structured self-monitoring blood glucose regimen. *Samj South African Medical Journal*, 108(8):654–659. WOS:000440975600016.

[216] Kamdi, M. K. A., Shafei, M. N., Musa, K. I., Hanafi, M. H., and Suliman, M. A. (2023). Comparison of the Modified Barthel Index (MBI) Score Trends Among Workers With Stroke Receiving Robotic and Conventional Rehabilitation Therapy. *Cureus*, 15(1):e34207.

[217] Karp, N. A. and Fry, D. (2021). What is the optimum design for my animal experiment? *BMJ Open Science*, 5(1):e100126. Publisher: BMJ Specialist Journals Section: Review.

[218] Kasozi, N., Wilhelmi, B., and Kaiser, H. (2021). The effect of the addition of a probiotic mixture of two Bacillus species to a coupled aquaponics system on water quality, growth and digestive enzyme activity of Mozambique tilapia, Oreochromis mossambicus. *Journal of Applied Aquaculture*, 0(0):1–19. Publisher: Taylor & Francis _eprint: https://doi.org/10.1080/10454438.2021.1986192.

[219] Katsogiannou, E. G., Athanasiou, L. V., Katsoulos, P. D., Polizopoulou, Z. S., Tzivara, A., and Christodoulopoulos, G. (2020). Estimation of white blood cell and platelet counts in ovine blood smears, and a comparison with the ADVIA 120 hematology analyzer. *Veterinary Clinical Pathology*, 49(2):222–226. Place: Hoboken Publisher: Wiley WOS:000543115200005.

[220] Katsogiannou, E. G., Katsoulos, P. D., Ziogas, C., Naskou, M. C., Christodoulopoulos, G., Polizopoulou, Z. S., Tzivara, A., and Athanasiou, L. (2021). Blood cell count and morphology, and vitamin B12 concentration in pre- and post-weaned calves. *Veterinární Medicína*, 66(12):513–519. Place: Prague Publisher: Czech Academy of Agricultural Sciences (CAAS).

[221] Katsoulos, P. D., Athanasiou, L. V., Karatzia, M. A., Valasi, I., Boscos, C., and Karatzias, H. (2016). Comparison of a non-contact infrared thermometer with a rectal digital thermometer for use in ewes. *Small Ruminant Research*, 143:84–88.

[222] Katsoulos, P. D., Karatzia, M. A., Dovas, C. I., Filioussis, G., Papadopoulos, E., Kiossis, E., Arsenopoulos, K., Papadopoulos, T., Boscos, C., and Karatzias, H. (2017). Evaluation of the in-field efficacy of oregano essential oil administration on the control of neonatal diarrhea syndrome in calves. *Research in Veterinary Science*, 115:478–483.

[223] Katsoulos, P. D., MA Karatzia, Dedousi, A., Camo, D., and Boscos, C. (2020). Milk consumption monitoring as a farmer friendly indicator for advanced treatment in limited fed calves with neonatal diarrhoea syndrome. *Veterinární Medicína*, 65(3):104–110. Place: Prague Publisher: Czech Academy of Agricultural Sciences (CAAS).

[224] Katz, L., Tata, A., Woolman, M., and Zarrine-Afsar, A. (2021). Lipid Profiling in Cancer Diagnosis with Hand-Held Ambient Mass Spectrometry Probes: Addressing the

Late-Stage Performance Concerns. *Metabolites*, 11(10):660. Number: 10 Publisher: Multidisciplinary Digital Publishing Institute.

[225] Keating, C. T. and Cook, J. L. (2023). The inside out model of emotion recognition: how the shape of one's internal emotional landscape influences the recognition of others' emotions. *Scientific Reports (Nature Publisher Group)*, 13(1):21490. Place: London Publisher: Nature Publishing Group.

[226] Keating, C. T., Fraser, D. S., Sowden, S., and Cook, J. L. (2021). Differences Between Autistic and Non-Autistic Adults in the Recognition of Anger from Facial Motion Remain after Controlling for Alexithymia. *Journal of Autism and Developmental Disorders*.

[227] Keene, T., Pammer, K., Lord, B., and Shipp, C. (2022). Fluency and confidence predict paramedic diagnostic intuition: An experimental study of applied dual-process theory. *International Emergency Nursing*, 61:101126.

[228] Kerpan, S., Humbert, M. L., Rodgers, C. D., and Stoddart, A. L. (2019). Improving Kindergarten and Grade One Indigenous Students' On-Task Behavior With the Use of Movement Integration. *Journal of American Indian Education*, 58(1-2):84–107. Publisher: University of Minnesota Press.

[229] Kersbergen, I., Copeland, A., Pryce, R., Meier, P., and Field, M. (2024). The effect of proportional pricing on alcohol purchasing in two online experiments. *Addiction*, n/a(n/a). _eprint: https://onlinelibrary.wiley.com/doi/pdf/10.1111/add.16723.

[230] Keyser, H. H. D., Brinton, J. T., Bothwell, S., Camacho, M., Kempe, A., and Szefler, S. J. (2023). Encouraging adherence in adolescents with asthma using financial incentives: An RCT. *Pediatric Pulmonology*, n/a(n/a). _eprint: https://onlinelibrary.wiley.com/doi/pdf/10.1002/ppul.26594.

[231] Kim, A., Yang, E. J., Ji, M., Beom, J., and Yi, C. (2022). Distorted body schema after mastectomy with immediate breast reconstruction: a 4-month follow up study. *PeerJ*, 10:e14157.

[232] Kleif, J., Hauge, C. I., Vilandt, J., and Gögenur, I. (2018). Randomized clinical trial of preoperative high-dose methylprednisolone on postoperative pain at rest after laparoscopic appendectomy. *Anesthesia & Analgesia*, 126(5):1712–1720.

[233] Komro, K. A., Livingston, M. D., Kominsky, T. K., Livingston, B. J., Garrett, B. A., Molina, M. M., and Boyd, M. L. (2015). Fifteen-minute comprehensive alcohol risk survey: reliability and validity across american indian and white adolescents. *Journal of Studies on Alcohol and Drugs*, 76(1):133–142. WOS:000348625300014.

[234] Koumantakis, G. A., Lountzis, D., Papatsimpas, G., Kentritas, O., Katsiki, X., and Michaleas, P. (2019). Effects of a functional lower extremity fatigue protocol and a 5-minute recovery period on the performance of a single leg hop test for distance in healthy participants. *Journal of Sports Medicine and Physical Fitness*, 59(6):916–924. WOS:000471836200004.

[235] Krause-Parello, C. A., Friedmann, E., Blanchard, K., Payton, M., and Gee, N. R. (2020). Veterans and shelter dogs: examining the impact of a dog-walking intervention on physiological and post-traumatic stress symptoms. *Anthrozoos*, 33(2):225–241. Place: Abingdon Publisher: Routledge Journals, Taylor & Francis Ltd WOS:000519494500005.

[236] Kreidler, S. M., Ringham, B. M., Muller, K. E., and Glueck, D. H. (2018). Calculating power for the general linear multivariate model with one or more Gaussian covariates. *Communications in Statistics-Theory and Methods*, pages 1–14.

[237] Kreidler, S. M., Ringham, B. M., Muller, K. E., and Glueck, D. H. (2021). A power approximation for the Kenward and Roger Wald test in the linear mixed model. *PLOS ONE*, 16(7):e0254811. Publisher: Public Library of Science.

[238] Krishnan, S., Watkins, K. E., and Bishop, D. V. M. (2017). The effect of recall, reproduction, and restudy on word learning: a pre-registered study. *BMC psychology*, 5(1):28.

[239] Kujawska, A., Androsiuk, J., Perkowski, R., Kujawski, S., Simon, C. B., Bhatt, R. R., Jahanshad, N., Hapidou, E. G., Cai, Y., Hajec, W., Husejko, J., Zalewski, P., and Kędziora-Kornatowska, K. (2025). A network analysis of changing pain cooccurrence in older adults findings from the second wave of the COPERNICUS study. *Scientific Reports*, 15(1):12369. Publisher: Nature Publishing Group.

[240] Kujawska, A., Kujawski, S., Hajec, W., Skierkowska, N., Kwiatkowska, M., Husejko, J., Newton, J. L., Simoes, J. A., Zalewski, P., and Kędziora-Kornatowska, K. (2021). Coffee Consumption and Blood Pressure: Results of the Second Wave of the Cognition of Older People, Education, Recreational Activities, Nutrition, Comorbidities, and Functional Capacity Studies (COPERNICUS). *Nutrients*, 13(10):3372.

[241] Kujawski, S., Kujawska, A., Kozakiewicz, M., Jakovljevic, D. G., Stankiewicz, B., Newton, J. L., Kędziora-Kornatowska, K., and Zalewski, P. (2022a). Effects of Sitting Callisthenic Balance and Resistance Exercise Programs on Cognitive Function in Older Participants. *International Journal of Environmental Research and Public Health*, 19(22):14925. Number: 22 Publisher: Multidisciplinary Digital Publishing Institute.

[242] Kujawski, S., Kujawska, A., Perkowski, R., Androsiuk-Perkowska, J., Hajec, W., Kwiatkowska, M., Skierkowska, N., Husejko, J., Bieniek, D., Newton, J. L., Morten, K. J., Zalewski, P., and Kędziora-Kornatowska, K. (2021). Cognitive Function Changes in Older People. Results of Second Wave of Cognition of Older People, Education, Recreational Activities, NutritIon, Comorbidities, fUnctional Capacity Studies (COPERNICUS). *Frontiers in Aging Neuroscience*, 13. Publisher: Frontiers.

[243] Kujawski, S., Słomko, J., Godlewska, B. R., Cudnoch-Jędrzejewska, A., Murovska, M., Newton, J. L., Sokołowski, Ł., and Zalewski, P. (2022b). Combination of whole body cryotherapy with static stretching exercises reduces fatigue and improves functioning of the autonomic nervous system in Chronic Fatigue Syndrome. *Journal of Translational Medicine*, 20:1–15. Place: London Publisher: BioMed Central.

[244] Kuk, F., Slugocki, C., Davis-Ruperto, N., and Korhonen, P. (2022). Measuring the effect of adaptive directionality and split processing on noise acceptance at multiple input levels. *International Journal of Audiology*, 0(0):1–9. Publisher: Taylor & Francis _eprint: https://doi.org/10.1080/14992027.2021.2022789.

[245] Kuppusamy, M., Ta, H. Q., Davenport, H. N., Bazaz, A., Kulshrestha, A., Daneva, Z., Chen, Y.-L., Carrott, P. W., Laubach, V. E., and Sonkusare, S. K. (2023). Purinergic P2Y2 receptor-induced activation of endothelial TRPV4 channels mediates lung ischemia-reperfusion injury. *Science Signaling*, 16(808):eadg1553.

[246] Kurnat-Thoma, E. L., Roberts, M. M., and Corcoran, E. B. (2016). Perioperative heat loss prevention-a feasibility trial. *AORN journal*, 104(4):307–319.

[247] Kurz, A., Reichenberg, R. E., Elliott, S. N., and Yel, N. (2020). Opportunity-to-learn performance levels and student achievement gains for students with and without disabilities. *Teaching and Teacher Education*, 94:103092.

[248] Kwan, R. Y. C., Liu, J., Sin, O. S. K., Fong, K. N. K., Qin, J., Wong, J. C. Y., and Lai, C. (2024). Effects of Virtual Reality Motor-Cognitive Training for Older People With Cognitive Frailty: Multicentered Randomized Controlled Trial. *Journal of Medical Internet Research*, 26:e57809.

[249] Kwan, R. Y. C., Liu, J. Y. W., Lee, P. H., Sin, O. S. K., Wong, J. S. W., Fu, M. R., and Suen, L. K. P. (2023). The effects of an e-health brisk walking intervention in increasing moderate-to-vigorous physical activity in physically inactive older people with cognitive frailty: study protocol for a randomized controlled trial. *Trials*, 24(1):1–12. Number: 1 Publisher: BioMed Central.

[250] La Torre, D., Dalile, B., de Loor, H., Van Oudenhove, L., and Verbeke, K. (2021). Changes in kynurenine pathway metabolites after acute psychosocial stress in healthy males: a single-arm pilot study. *Stress (Amsterdam, Netherlands)*, 24(6):920–930.

[251] Laczó, M., Svacova, Z., Lerch, O., Martinkovic, L., Krejci, M., Nedelska, Z., Horakova, H., Matoska, V., Vyhnalek, M., Hort, J., Hornberger, M., and Laczó, J. (2025). Spatial navigation deficits in early Alzheimer's disease: the role of biomarkers and APOE genotype. *Journal of Neurology*, 272(6):438.

[252] Lahiri, A. and Chakraborty, A. (2019). Predictors of weight velocity in the first 6 months of life in a rural block of west bengal: a longitudinal study. *Indian Journal of Community Medicine: Official Publication of Indian Association of Preventive & Social Medicine*, 44(2):92–96.

[253] Lang, S., Gan, L. S., Alrazi, T., and Monchi, O. (2019). Theta band high definition transcranial alternating current stimulation, but not transcranial direct current stimulation, improves associative memory performance. *Scientific Reports*, 9(1):8562.

[254] Lara, K. H., Kramer, H. J., and Lagattuta, K. H. (2021). This is not what I expected: The impact of prior expectations on children's and adults' preferences and emotions. *Developmental Psychology*, 57(5):702–717.

[255] Larsen, D. B., Graven-Nielsen, T., and Boudreau, S. A. (2019). Pain-induced reduction in corticomotor excitability is counteracted by combined action-observation and motor imagery. *Journal of Pain*, 20(11):1307–1316. WOS:000497889200005.

[256] Larson, M. J. and Carbine, K. A. (2017). Sample size calculations in human electrophysiology (EEG and ERP) studies: A systematic review and recommendations for increased rigor. *International Journal of Psychophysiology*, 111:33–41. WOS:000392554600004.

[257] Lau, J., Peh, C. H., Ng, A., Koh, W.-L., Luo, N., and Tan, K.-K. (2025). Does adjuvant chemotherapy result in poorer health-related quality of life among colorectal cancer patients? A longitudinal multisite observational study in Singapore. *Health and Quality of Life Outcomes*, 23(1):1–12. Number: 1 Publisher: BioMed Central.

[258] Lazic, S. E. (2016). *Experimental design for laboratory biologists maximising information and improving reproducibility preface.* Cambridge Univ Press, Cambridge. WOS:000403686500001.

[259] Le Cong, D., Sato, D., Ikarashi, K., Ochi, G., Fujimoto, T., and Yamashiro, K. (2024). No effect of whole-hand water flow stimulation on skill acquisition and retention during sensorimotor adaptation. *Frontiers in Human Neuroscience.* Place: Lausanne Publisher: Frontiers Research Foundation.

[260] Lee, M.-S., Eum, K.-D., Fang, S. C., Rodrigues, E. G., Modest, G. A., and Christiani, D. C. (2014). Oxidative stress and systemic inflammation as modifiers of cardiac autonomic responses to particulate air pollution. *International Journal of Cardiology*, 176(1):166–170. WOS:000341040900035.

[261] Leontidis, G., Al-Diri, B., and Hunter, A. (2016). Summarising the retinal vascular calibres in healthy, diabetic and diabetic retinopathy eyes. *Computers in Biology and Medicine*, 72:65–74. WOS:000375812500007.

[262] Leshin, R. A., Lei, R. F., Byrne, M., and Rhodes, M. (2021). Who is a typical woman? Exploring variation in how race biases representations of gender across development. *Developmental Science*, page e13175.

[263] Leventhal, A. M., Goldenson, N. I., Cho, J., Kirkpatrick, M. G., McConnell, R. S., Stone, M. D., Pang, R. D., Audrain-McGovern, J., and Barrington-Trimis, J. L. (2019). Flavored E-cigarette Use and Progression of Vaping in Adolescents. *Pediatrics*, 144(5).

[264] Li, Y., Chien, W. T., and Bressington, D. (2020). Effects of a coping-oriented supportive programme for people with spinal cord injury during inpatient rehabilitation: a quasi-experimental study. *Spinal Cord*, 58(1):58–69.

[265] Licciardone, J., Brownell, E., Nwaichi, U., Patel, A., and Do, K. (2025). Longitudinal outcomes among patients with fibromyalgia, chronic widespread pain, or localized chronic low back pain. *Journal of osteopathic medicine.*

[266] Licciardone, J. C., Tran, Y., Ngo, K., Toledo, D., Peddireddy, N., and Aryal, S. (2024). Physician Empathy and Chronic Pain Outcomes. *JAMA network open*, 7(4):e246026.

[267] Liew, B. X. W., Morris, S., Keogh, J. W. L., Appleby, B., and Netto, K. (2016). Effects of two neuromuscular training programs on running biomechanics with load carriage: a study protocol for a randomised controlled trial. *Bmc Musculoskeletal Disorders*, 17:445. WOS:000395050700003.

[268] Lim, C. W., Diaconita, V., Liu, E., Ault, N., Lizotte, D., Nguyen, M., and Hutnik, C. M. L. (2019). Effect of 6-week washout period on intraocular pressure following chronic prostaglandin analogue treatment: a randomized controlled trial. *Canadian Journal of Ophthalmology. Journal Canadien D'ophtalmologie.*

[269] Lim, D. W., Phua, H. P., Ho, Y. M., Lee, L. T., and Chow, A. (2023). Effectiveness of twice daily oil-based moisturizer in preventing skin dryness among healthcare workers. *Contact Dermatitis*, 88(3):236–238. _eprint: https://onlinelibrary.wiley.com/doi/pdf/10.1111/cod.14259.

[270] Lim, D. W., Retrouvey, H., Kerrebijn, I., Butler, K., O'Neill, A. C., Cil, T. D., Zhong, T., Hofer, S. O. P., McCready, D. R., and Metcalfe, K. A. (2021). Longitudinal Study of Psychosocial Outcomes Following Surgery in Women with Unilateral Nonhereditary Breast Cancer. *Annals of Surgical Oncology*.

[271] Lin, Y.-C., Lee, Y.-C., Hsu, T.-Y., Liao, Y.-C., and Soong, B.-W. (2019). Comparable progression of spinocerebellar ataxias between Caucasians and Chinese. *Parkinsonism & Related Disorders*, 62:156–162. WOS:000476961700025.

[272] Ling, J., Poon, E. W. M., Yang, A., Yeung, T., Loo, K., Ozaki, R., Ma, R. C. W., Luk, A. O. Y., Kong, A. P. S., Chan, J. C. N., and Chow, E. (2021). Glycemic Variability and Time in Range During Self-titration of Once Daily Insulin Glargine 300 U/ml Versus Neutral Protamine Hagedorn Insulin in Insulin-naïve Chinese Type 2 Diabetes Patients. *Diabetes Therapy*, 12(5):1399–1413.

[273] Linke, J. O., Jones, E., Pagliaccio, D., Swetlitz, C., Lewis, K. M., Silverman, W. K., Bar-Haim, Y., Pine, D. S., and Brotman, M. A. (2019). Efficacy and mechanisms underlying a gamified attention bias modification training in anxious youth: protocol for a randomized controlled trial. *BMC psychiatry*, 19(1):246.

[274] Litt, J. S., Alaimo, K., Harrall, K. K., Hamman, R. F., Hébert, J. R., Hurley, T. G., Leiferman, J. A., Li, K., Villalobos, A., Coringrato, E., Courtney, J. B., Payton, M., and Glueck, D. H. (2023). Effects of a community gardening intervention on diet, physical activity, and anthropometry outcomes in the USA (CAPS): an observer-blind, randomised controlled trial. *The Lancet. Planetary Health*, 7(1):e23–e32.

[275] Liu, L., Kilduff, M., Lee, S., and Fisher, C. (2025). Buffered by reflected glory? The effects of star connections on career outcomes. *Journal of Applied Psychology*. Publisher: American Psychological Association.

[276] Loffing, F., Soelter, F., Hagemann, N., and Strauss, B. (2016). On-court position and handedness in visual anticipation of stroke direction in tennis. *Psychology of Sport and Exercise*, 27:195–204. WOS:000385472800022.

[277] Lohse, K. R., Miller, M. W., Daou, M., Valerius, W., and Jones, M. (2020). Dissociating the contributions of reward-prediction errors to trial-level adaptation and long-term learning. *Biological Psychology*, 149:107775. WOS:000504649500006.

[278] Lopez-Montoyo, A., Quero, S., Montero-Marin, J., Barcelo-Soler, A., Beltran, M., Campos, D., and Garcia-Campayo, J. (2019). Effectiveness of a brief psychological mindfulness-based intervention for the treatment of depression in primary care: study protocol for a randomized controlled clinical trial. *BMC psychiatry*, 19(1):301.

[279] Lossada-Soto, E., Pazik, M., Horodyski, M. B., Vasilopoulos, T., de Faria, L. B., Mathews, C., and Hagen, J. (2022). Can fluoxetine mitigate mental health decline in musculoskeletal trauma patients: a pilot single-center randomized clinical trial. *Pilot and Feasibility Studies*, 8(1):184.

[280] Luciano, F., Ruggiero, L., and Pavei, G. (2021). Sample size estimation in locomotion kinematics and electromyography for statistical parametric mapping. *Journal of Biomechanics*, 122:110481.

[281] Lukic, N., Saxer, T., Hou, M.-Y., Zumbrunn Wojczyńska, A., Gallo, L. M., and Colombo, V. (2021). Short-term effects of NTI-tss and Michigan splint on nocturnal jaw muscle activity: A pilot study. *Clinical and Experimental Dental Research*, 7(3):323–330.

[282] Lunkenheimer, E., Busuito, A., Brown, K. M., Panlilio, C., and Skowron, E. A. (2019). The interpersonal neurobiology of child maltreatment: parasympathetic substrates of interactive repair in maltreating and nonmaltreating mother-child dyads. *Child Maltreatment*, 24(4):353–363. WOS:000492405900003.

[283] Lunkenheimer, E., Busuito, A., Brown, K. M., and Skowron, E. A. (2018). Mother-child coregulation of parasympathetic processes differs by child maltreatment severity and subtype. *Child Maltreatment*, 23(3):211–220. WOS:000438060100001.

[284] López-Larrosa, S., Sánchez-Souto, V., Ha, A. P., and Cummings, E. M. (2019). Emotional security and interparental conflict: responses of adolescents from different living arrangements. *Journal of Child and Family Studies*, pages 1–13.

[285] Madill, C., Chacon, A., Kirby, E., Novakovic, D., and Nguyen, D. D. (2021). Active Ingredients of Voice Therapy for Muscle Tension Voice Disorders: A Retrospective Data Audit. *Journal of Clinical Medicine*, 10(18):4135.

[286] Mak, Y.-W., Leung, D. Y. P., Zhang, X., Chung, J. O. K., Chow, P. S. Y., Ruan, J., and Yeung, J. W. F. (2024). Effects of a flexibly delivered group-based acceptance and commitment therapy on reducing stress and enhancing psychological wellbeing in parents of school-age children during the COVID-19 pandemic: a quasi-experimental study. *Frontiers in Public Health*, 12. Publisher: Frontiers.

[287] Malisoux, L., Gette, P., Chambon, N., Urhausen, A., and Theisen, D. (2017). Adaptation of running pattern to the drop of standard cushioned shoes: A randomised controlled trial with a 6-month follow-up. *Journal of Science and Medicine in Sport*, 20(8):734–739.

[288] Mallikarjun, A., Shroads, E., and Newman, R. S. (2024). Perception of vocoded speech in domestic dogs. *Animal Cognition*, 27(1):34.

[289] Marchini, A., Pedroso, W., and Neto, O. P. (2019). Mixed Modal Training to Help Older Adults Maintain Postural Balance. *Journal of Chiropractic Medicine*, 18(3):198–204.

[290] Marcozzi, S., Bigossi, G., Giuliani, M. E., Giacconi, R., Cardelli, M., Piacenza, F., Orlando, F., Segala, A., Valerio, A., Nisoli, E., Brunetti, D., Puca, A., Boschi, F., Gaetano, C., Mongelli, A., Lattanzio, F., Provinciali, M., and Malavolta, M. (2023). Comprehensive longitudinal non-invasive quantification of healthspan and frailty in a large cohort (n = 546) of geriatric C57BL/6 J mice. *GeroScience*.

[291] Margato, L. R., de Souza Lino, A. D., de Sousa, W. G., Pelet, D. C. S., de Oliveira, E. P., Orsatti, F. L., and Souza, M. V. C. (2022). Pro-inflammatory diet index is negatively associated with physical performance in postmenopausal women: a cross-sectional study. *Nutrire*, 47(2):27. Place: San Paulo Publisher: Springer Nature B.V.

[292] Martínez-Jiménez, M. A., Kolosovas-Machuca, S., Alcocer, F., Ortiz-Dosal, L. C., González-Grijalva, M. C., Delsol-Revuelta, G. A., Gaitan-Gaona, F. I., Valadez-Castillo, F. J., and Ramirez-GarciaLuna, J. L. (2022). A Randomized Controlled Trial on the

Effect of Local Insulin Glargine on Venous Ulcer Healing. *Journal of Surgical Research*, 279:657–665.

[293] Mastrogiovanni, C., Rosenbaum, S., Delbaere, K., Tiedemann, A., Teasdale, S., McGavin, A., Briggs, N., and McKeon, G. (2022). A mental health-informed, online health promotion programme targeting physical activity and healthy eating for adults aged 60+ years: study protocol for the MovingTogether randomised controlled trial. *Trials*, 23(1):1052.

[294] Matthews, M., Webb, T. L., and Sheppes, G. (2021). Do people choose the same strategies to regulate other people's emotions as they choose to regulate their own? *Emotion (Washington, D.C.)*.

[295] Mbelele, P. M., Mpolya, E. A., Sauli, E., Mtafya, B., Ntinginya, N. E., Addo, K. K., Kreppel, K., Mfinanga, S., Phillips, P. P. J., Gillespie, S. H., Heysell, S. K., Sabiiti, W., and Mpagama, S. G. (2021). Mycobactericidal Effects of Different Regimens Measured by Molecular Bacterial Load Assay among People Treated for Multidrug-Resistant Tuberculosis in Tanzania. *Journal of Clinical Microbiology*, 59(4):e02927–20.

[296] McClure, L. A., Lee, N. L., Sand, K., Vivanti, G., Fein, D., Stahmer, A., and Robins, D. L. (2021). Connecting the Dots: a cluster-randomized clinical trial integrating standardized autism spectrum disorders screening, high-quality treatment, and long-term outcomes. *Trials*, 22(1):319.

[297] McGarr, G. W., Li-Maloney, C., King, K. E., Janetos, K.-M. T., Fujii, N., Amano, T., and Kenny, G. P. (2024). Modulation of cutaneous vasodilation by reactive oxygen species during local and whole body heating in young and older adults. *American Journal of Physiology-Regulatory, Integrative and Comparative Physiology*, 327(6):R543–R552. Publisher: American Physiological Society.

[298] McNamara, M. E., Zisser, M., Beevers, C. G., and Shumake, J. (2022). Not just "big" data: Importance of sample size, measurement error, and uninformative predictors for developing prognostic models for digital interventions. *Behaviour Research and Therapy*, 153:104086.

[299] McNeer, R. R., Bennett, C. L., and Dudaryk, R. (2016). Intraoperative noise increases perceived task load and fatigue in anesthesiology residents: a simulation-based study. *Anesthesia & Analgesia*, 122(2):512–525.

[300] Mesquita, R. M., Morin, J.-B., and Dewolf, A. H. (2024a). The Effect of Step Frequency and Running Speed on the Coordination of the Pelvis and Thigh Segments During Running. *Journal of Applied Biomechanics*, pages 1–10.

[301] Mesquita, R. M., Willems, P. A., Dewolf, A. H., and Catavitello, G. (2024b). Kinetics and mechanical work done to move the body centre of mass along a curve. *PLOS ONE*, 19(2):e0298790.

[302] Mheissen, S., Seehra, J., Khan, H., and Pandis, N. (2022). Do sample size calculations in longitudinal orthodontic trials use the advantages of this study design?: A meta-epidemiological study. *The Angle Orthodontist*.

[303] Miko, S. C., Simon, J. E., Monfort, S. M., Yom, J. P., Ulloa, S., and Grooms, D. R. (2021). Postural stability during visual-based cognitive and motor dual-tasks after ACLR. *Journal of Science and Medicine in Sport*, 24(2):146–151.

[304] Miller, A., Reisman, D. S., Billinger, S. A., Dunning, K., Doren, S., Ward, J., Wright, H., Wagner, E., Carl, D., Gerson, M., Awosika, O., Khoury, J., Kissela, B., and Boyne, P. (2021). Moderate-intensity exercise versus high-intensity interval training to recover walking post-stroke: protocol for a randomized controlled trial. *Trials*, 22(1):457.

[305] Miller, J., MacDermid, J. C., Walton, D. M., and Richardson, J. (2015). Chronic pain self-management support with pain science education and exercise (COMMENCE): study protocol for a randomized controlled trial. *Trials*, 16:462.

[306] Mills, A. C., Badour, C. L., Korte, K. J., Killeen, T. K., Henschel, A. V., and Back, S. E. (2017). Integrated treatment of PTSD and substance use disorders: Examination of imaginal exposure length. *Journal of traumatic stress*, 30(2):166–172.

[307] Minelli, A., Barlati, S., Vitali, E., Bignotti, S., Dattilo, V., Tura, G. B., Maffioletti, E., Giacopuzzi, E., Santoro, V., Perusi, G., Cobelli, C., Magri, C., Bonizzato, S., Bocchio-Chiavetto, L., Spina, E., Vita, A., and Gennarelli, M. (2021). Clinical validation of a combinatorial PharmAcogeNomic approach in major Depressive disorder: an Observational prospective RAndomized, participant and rater-blinded, controlled trial (PANDORA trial). *Trials*, 22(1):896. Place: London Publisher: BioMed Central.

[308] Mirabella, G., Mancini, C., Pacifici, S., Guerrini, D., and Terrinoni, A. (2023). Enhanced reactive inhibition in adolescents with non-suicidal self-injury disorder. *Developmental Medicine & Child Neurology*, n/a(n/a). _eprint: https://onlinelibrary.wiley.com/doi/pdf/10.1111/dmcn.15794.

[309] Mirabella, G., Pilotto, A., Rizzardi, A., Montalti, M., Olivola, E., Zatti, C., Di Caprio, V., Ferrari, E., Modugno, N., and Padovani, A. (2024). Effects of dopaminergic treatment on inhibitory control differ across Hoehn and Yahr stages of Parkinson's disease. *Brain Communications*, 6(1):fcad350.

[310] Mitchell, E. J., Thomson, D. M., Openshaw, R. L., Bristow, G. C., Dawson, N., Pratt, J. A., and Morris, B. J. (2020). Drug-responsive autism phenotypes in the 16p11.2 deletion mouse model: a central role for gene-environment interactions. *Scientific Reports*, 10(1):12303.

[311] Mobley, M., Yang, Y., Yanai, R., Nelson, K., Bacon, A., Heine, P., and Richter, D. (2019). How to Estimate Statistically Detectable Trends in a Time Series: A Study of Soil Carbon and Nutrient Concentrations at the Calhoun LTSE. *Soil Science Society of America Journal*, 83.

[312] Monfort, S. M., Simon, J. E., Miko, S. C., and Grooms, D. R. (2022). Effects of cognitive- and motor-dual tasks on postural control regularity following anterior cruciate ligament reconstruction. *Gait & Posture*, 97:109–114.

[313] Moran, J. M., Aliaga, I., and Pedrera-Zamorano, J. D. (2017). The importance of accurate sample size calculations in clinical trials. *Archives of Osteoporosis*, 12(1):10.

[314] Moret-Tatay, C., García-Ramos, D., Sáiz-Mauleón, B., Gamermann, D., Bertheaux, C., and Borg, C. (2021). Word and Face Recognition Processing Based on Response Times and Ex-Gaussian Components. *Entropy (Basel, Switzerland)*, 23(5):580.

[315] Morris, J. F., Murphy, J., Fagerli, K., Schneeberger, C., Jaron, P., Moke, F., Juma, J., Ochieng, J. B., Omore, R., Roellig, D., Xiao, L., Priest, J. W., Narayanan, J., Montgomery, J. M., Hill, V., Mintz, E., Ayers, T. L., and O'Reilly, C. E. (2018). A Randomized Controlled Trial to Assess the Impact of Ceramic Water Filters on Prevention of Diarrhea and Cryptosporidiosis in Infants and Young Children-Western Kenya, 2013. *The American Journal of Tropical Medicine and Hygiene*, 98(5):1260–1268.

[316] Mouilso, E. R., Tuerk, P. W., Schnurr, P. P., and Rauch, S. A. M. (2016). Addressing the gender gap: Prolonged exposure for PTSD in veterans. *Psychological Services*, 13(3):308–316. Publisher: Educational Publishing Foundation, Educational Publishing Foundation.

[317] Moussavi, Z., Rutherford, G., Lithgow, B., Millikin, C., Modirrousta, M., Mansouri, B., Wang, X., Omelan, C., Fellows, L., Fitzgerald, P., and Koski, L. (2021). Repeated Transcranial Magnetic Stimulation for Improving Cognition in Patients With Alzheimer Disease: Protocol for a Randomized, Double-Blind, Placebo-Controlled Trial. *JMIR research protocols*, 10(1):e25144.

[318] Mukololo, D. L., Sijumbila, D. G., Muyangwa-Semenova, D. M., Mukabila, M. M., and Munthali, D. J. (2024). Bone-Alkaline Phosphatase is a Potential Biomarker for Monitoring Femur Fracture Healing. *Medical Journal of Zambia*, 51(3):200–211. Number: 3.

[319] Mumu, S. J., Fahey, P. P., Ali, L., A K M Fazlur Rahman, and Merom, D. (2019). Seasonal Variations in Physical Activity Domains among Rural and Urban Bangladeshis Using a Culturally Relevant Past Year Physical Activity Questionnaire (PYPAQ). *Journal of Environmental and Public Health*, 2019:9. Place: New York Publisher: Hindawi Limited.

[320] Munjal, A., Sakhadeo, U. R., Muller, K. E., Glueck, D. H., and Kreidler, S. M. (2014). GLIMMPSE Lite: Calculating Power and Sample Size on Smartphone Devices. *PLOS ONE*, 9(12):e102082. Publisher: Public Library of Science.

[321] Murgatroyd, D. F., Harris, I. A., Tran, Y., and Cameron, I. D. (2016). The association between seeking financial compensation and injury recovery following motor vehicle related orthopaedic trauma. *BMC musculoskeletal disorders*, 17:282.

[322] Murray, D. M., Taljaard, M., Turner, E. L., and George, S. M. (2020). Essential Ingredients and Innovations in the Design and Analysis of Group-Randomized Trials. *Annual Review of Public Health, Vol 41*, 41:1–19. WOS:000524457700002.

[323] Myers, V. R., McKillop, A. L., Fraser, S. J., Abel, J. M., and Wells, G. D. (2017). Physiological and psychological adaptations during taper in competitive swimmers. *International Journal of Sports Science & Coaching*, 12(4):481–494. Publisher: SAGE Publications.

[324] Nash, L., Cheung, V. C. K., Gupta, A., Cheung, R. T. H., He, B., Liston, M., and Thomson, D. (2024). The effects of age and physical activity status on muscle synergies when walking down slopes. *European Journal of Applied Physiology.*

[325] Newell, S. M., Logan, H. L., Guo, Y., Marks, J. G., and Shepperd, J. A. (2015). Evaluating tablet computers as a survey tool in rural communities. *The Journal of Rural Health: Official Journal of the American Rural Health Association and the National Rural Health Care Association*, 31(1):108–117.

[326] Nip, I. S. B. and Garellek, M. (2021). Voice Quality of Children With Cerebral Palsy. *Journal of Speech, Language, and Hearing Research*, 64(8):3051–3059. Publisher: American Speech-Language-Hearing Association.

[327] Nix, C. A., Dozier, M. E., Porter, B., and Ayers, C. R. (2024). Clinician Sentiments Related to Implementation of Evidence-Based Treatment for Hoarding in Older Adults. *Journal of Psychopathology and Behavioral Assessment*, 46(3):683–694.

[328] Nobel, E., Hoekstra, P. J., Agnes Brunnekreef, J., Messink-De Vries, D. E. H., Fischer, B., Emmelkamp, P. M. G., and Van Den Hoofdakker, B. J. (2019). Home-based parent training for school-aged children with attention-deficit/hyperactivity disorder and behavior problems with remaining impairing disruptive behaviors after routine treatment: a randomized controlled trial. *European child & adolescent psychiatry*, pages 1–14.

[329] Nordgren, R. (2019). Calculating a sample size for a study with repeated measures. *Journal of molecular and cellular cardiology.*

[330] Odland, H. H., Holm, T., Gammelsrud, L. O., Cornelussen, R., and Kongsgaard, E. (2021a). Determinants of LV dP/dtmax and QRS duration with different fusion strategies in cardiac resynchronisation therapy. *Open Heart*, 8(1). Place: London Publisher: BMJ Publishing Group LTD.

[331] Odland, H. H., Manuel Villegas-Martinez, Ross, S., Holm, T., Cornelussen, R., Remme, E. W., and Kongsgard, E. (2021b). Shortening of time-to-peak left ventricular pressure rise (Td) in cardiac resynchronization therapy. *ESC Heart Failure*, 8(6):5222–5236. Place: Oxford Publisher: John Wiley & Sons, Inc.

[332] Oexle, N., Lühr, M., Valacchi, D., and Rüsch, N. (2025). A web-based pilot randomized controlled trial to test the efficacy of education and contact-based interventions in reducing public suicide stigma. *BMC Psychiatry*, 25.

[333] Ojala, J., Vanhanen, J., Harno, H., Lioumis, P., Vaalto, S., Kaunisto, M. A., Putaala, J., Kangasniemi, M., Kirveskari, E., Mäkelä, J. P., and Kalso, E. (2021). A Randomized, Sham-Controlled Trial of Repetitive Transcranial Magnetic Stimulation Targeting M1 and S2 in Central Poststroke Pain: A Pilot Trial. *Neuromodulation: Journal of the International Neuromodulation Society.*

[334] Olsen, T., Stolt, E., Øvrebø, B., Elshorbagy, A., Tore, E. C., Lee-Ødegård, S., Hannibal Troensegaard, Johannessen, H., Doeland, B., Vo, A. A. D., Dahl, A. F., Svendsen, K., Thoresen, M., Refsum, H., Rising, R., and Barvíková, K. (2024). Dietary sulfur amino acid restriction in humans with overweight and obesity: a translational randomized controlled trial. *Journal of Translational Medicine*, 22:1–17. Place: London Publisher: BioMed Central.

[335] Ong, C. W. and Ito, K. (2021). Can't fight seeing sadness in tears: Measuring the implicit association between tears and sadness. *The British Journal of Social Psychology.*

[336] Oravec, D., Zaman, R., Rao, S., Chang, V., Divine, G., and Yeni, Y. N. (2025). Facet joint distance measurement using digital tomosynthesis while standing. *Journal of Biomechanics*, 183:112596.

[337] Ortell, K. K., Switonski, P. M., and Delaney, J. R. (2019). FairSubset: A tool to choose representative subsets of data for use with replicates or groups of different sample sizes. *Journal of Biological Methods*, 6(3):e118.

[338] Ozaki, A., Shishido, S., Nakamura, K., Harada, A., Katsuda, Y., Kanno, T., and Egusa, H. (2021). Impact of adhesive primer and light-curing on polymerization kinetics of self-adhesive resin cement in association with free radical reaction. *European Journal of Oral Sciences*, 129(6):e12828. _eprint: https://onlinelibrary.wiley.com/doi/pdf/10.1111/eos.12828.

[339] Paiva, C. E., Barroso, E. M., Carneseca, E. C., Souza, C. d. P., dos Santos, F. T., Mendoza Lopez, R. V., and Ribeiro Paiva, B. S. (2014). A critical analysis of test-retest reliability in instrument validation studies of cancer patients under palliative care: a systematic review. *BMC Medical Research Methodology*, 14:8. WOS:000331831000001.

[340] Pallotta, N., Vincoli, G., Pezzotti, P., Giovannone, M., Gigliozzi, A., Badiali, D., Vernia, P., and Corazziari, E. S. (2018). A risk score system to timely manage treatment in Crohn's disease: a cohort study. *Bmc Gastroenterology*, 18:164. WOS:000449343600002.

[341] Papamitsiou, Z. and Economides, A. (2020). The impact of on-demand metacognitive help on effortful behaviour: A longitudinal study using task-related visual analytics. *Journal of Computer Assisted Learning.*

[342] Parnes, S. C., Amritha Mallikarjun, Ramos, M. T., Stone, T. A., and Otto, C. M. (2023). A Randomized Cross-Over Study Comparing Cooling Methods for Exercise-Induced Hyperthermia in Working Dogs in Training. *Animals*, 13(23):3673. Place: Basel Publisher: MDPI AG.

[343] Parnes, S. C., Mallikarjun, A., Ramos, M. T., Capparell, T. R., and Otto, C. M. (2024). Voluntary head dunking after exercise-induced hyperthermia rapidly reduces core body temperature in dogs. *Journal of the American Veterinary Medical Association.* Section: Journal of the American Veterinary Medical Association.

[344] Parsarad, M., Ehtiati, S., Olazadeh, K., Dehghan, S. F., Ghorbani, M., Azimian, A., and Vaziri, M. H. (2025). Hematological biochemical and liver function changes associated with BTEX exposure in a six year retrospective cohort study. *Scientific Reports*, 15(1):5134. Publisher: Nature Publishing Group.

[345] Peng, R. H.-T., He, D., James, S. A., Williamson, J. N., Skadden, C., Jain, S., Hassaneen, W., Miranpuri, A., Kaur, A., Sarol, J. N., and Yang, Y. (2024). Determining the effects of targeted high-definition transcranial direct current stimulation on reducing post-stroke upper limb motor impairments-a randomized cross-over study. *Trials*, 25(1):34.

[346] Petrou, P. (2016). Long-term effect of tendering on prices of branded pharmaceutical products. *Health Policy and Technology*, 5(1):40–46. WOS:000373864000006.

[347] Phillips, H. N. and Heins, B. J. (2021). Evaluation of an herbal therapy to alleviate acute pain and stress of disbudded dairy calves under organic management. *Translational Animal Science*, 5(2).

[348] Pitkaaho, T., Partanen, P., Miettinen, M. H., and Vehvilainen-Julkunen, K. (2016). The relationship between nurse staffing and length of stay in acute-care: a one-year time-series data. *Journal of Nursing Management*, 24(5):571–579. WOS:000387213900002.

[349] Prochazka, J., Parilakova, K., Rudolf, P., Bruk, V., Jungwirthova, R., Fejtova, S., Masaryk, R., and Vaculik, M. (2022). Pain as Social Glue: A Preregistered Direct Replication of Experiment 2 of Bastian et al. (2014). *Psychological Science*, page 9567976211040745.

[350] Prologo, J. D., Gilliland, C. A., Miller, M., Harkey, P., Knight, J., Kies, D., Hawkins, C. M., Corn, D., Monson, D. K., Edalat, F., Dariushnia, S., and Brewster, L. (2017). Percutaneous Image-Guided Cryoablation for the Treatment of Phantom Limb Pain in Amputees: A Pilot Study. *Journal of Vascular and Interventional Radiology*, 28(1):24–34. WOS:000392465200004.

[351] Pugliese, J. M., Coyle, P. C., Knox, P. J., Sions, J. M., Patterson, C. G., Pohlig, R. T., Simon, C. B., Weiner, D. K., George, S. Z., Piva, S., and Hicks, G. E. (2022). The Manual Therapy and Strengthening for the Hip (MASH) Trial: Protocol for a Multisite Randomized Trial of a Subgroup of Older Adults With Chronic Back and Hip Pain. *Physical Therapy*, 102(1):pzab255.

[352] Quagebeur, R., Dalile, B., Raes, J., Lukas Van Oudenhove, Verbeke, K., and Vrieze, E. (2023). The role of short-chain fatty acids (SCFAs) in regulating stress responses, eating behavior, and nutritional state in anorexia nervosa: protocol for a randomized controlled trial. *Journal of Eating Disorders*, 11:1–12. Place: London Publisher: BioMed Central.

[353] Quinn, C. A., Walter, Z. C., de Andrade, D., Dingle, G., Haslam, C., and Hides, L. (2022). Controlled Trial Examining the Strength-Based Grit Wellbeing and Self-Regulation Program for Young People in Residential Settings for Substance Use. *International Journal of Environmental Research and Public Health*, 19(21):13835. Place: Basel Publisher: MDPI AG.

[354] Quint, W. H., Tadema, K. C. D., Crins, J. H. C., Kokke, N. C. C. J., Meester-Smoor, M. A., Willemsen, R., Klaver, C. C. W., and Iglesias, A. I. (2022). Zebrafish: An In Vivo Screening Model to Study Ocular Phenotypes. *Translational Vision Science & Technology*, 11(3):17.

[355] Quint, W. H., Tadema, K. C. D., Kokke, N. C. C. J., Meester-Smoor, M. A., Miller, A. C., Willemsen, R., Klaver, C. C. W., and Iglesias, A. I. (2023). Post-GWAS screening of candidate genes for refractive error in mutant zebrafish models. *Scientific Reports*, 13(1):2017.

[356] Qureshi, F., Kubzansky, L. D., Chen, Y., Soo, J., Kim, E. S., Lloyd-Jones, D., and Boehm, J. K. (2024). Associations between positive affect and physical activity from young

adulthood to midlife: A 25-year prospective study. *Health Psychology*, 43(10):730–738. Place: Washington Publisher: American Psychological Association, American Psychological Association Lawrence Erlbaum Associates.

[357] Rabau, S., Van Rompaey, V., and Van de Heyning, P. (2015). The effect of Transcranial Direct Current Stimulation in addition to Tinnitus Retraining Therapy for treatment of chronic tinnitus patients: a study protocol for a double-blind controlled randomised trial. *Trials*, 16(1):514. Place: London Publisher: BioMed Central.

[358] Radstaak, M., Huning, L., and Bohlmeijer, E. T. (2020). Well-being therapy as rehabilitation therapy for posttraumatic stress disorder symptoms: a randomized controlled trial. *Journal of Traumatic Stress*. Place: Hoboken Publisher: Wiley WOS:000525875900001.

[359] Randall, L. V., Green, M. J., and Huxley, J. N. (2018). Use of statistical modelling to investigate the pathogenesis of claw horn disruption lesions in dairy cattle. *Veterinary Journal*, 238:41–48. WOS:000444359700006.

[360] Ray, D. C., Burgin, E., Gutierrez, D., Ceballos, P., and Lindo, N. (2021). Child-centered play therapy and adverse childhood experiences: A randomized controlled trial. *Journal of Counseling & Development*, n/a(n/a). _eprint: https://onlinelibrary.wiley.com/doi/pdf/10.1002/jcad.12412.

[361] Raza, M. U. and Sivarao, D. V. (2021). Test-retest reliability of tone- and 40 Hz train-evoked gamma oscillations in female rats and their sensitivity to low-dose NMDA channel blockade. *Psychopharmacology*.

[362] Read, G. L. (2020). Processing Ambiguous Social Identity: Disclosure of Identity and Phenotypic Prototypicality Affect Processing and Evaluation of Persuasive Messages. *Journal of Communication*, 70(5):693–720. Publisher: Oxford Academic.

[363] Reed, C. A., DuBois, C. K., Hutchison, K. A., Huppert, T. J., and Monfort, S. M. (2022). Influence of serial subtraction tasks on transient characteristics of postural control. *Human Movement Science*, 83:102950.

[364] Reiter, A. J., Martin, J. A., Knurr, K. A., Adamczyk, P. G., and Thelen, D. G. (2024). Achilles Tendon Loading during Running Estimated Via Shear Wave Tensiometry: A Step Toward Wearable Kinetic Analysis. *Medicine and Science in Sports and Exercise*, 56(6):1077–1084.

[365] Rennels, J. L. and Verba, S. A. (2019). Gender typicality of faces affects children's categorization and judgments of women more than of men. *Sex Roles*, 81(5-6):355–369. WOS:000479251700007.

[366] Rhee, H., Love, T., Wicks, M. N., Tumiel-Berhalter, L., Sloand, E., Harrington, D., and Walters, L. (2021). Long-term Effectiveness of a Peer-Led Asthma Self-management Program on Asthma Outcomes in Adolescents Living in Urban Areas: A Randomized Clinical Trial. *JAMA network open*, 4(12):e2137492.

[367] Ribeiro Cunha, A. C., Silva, J. C., Caroline Pereira Garcês, Tássia Magnabosco Sisconeto, João Luiz Rezende Nascimento, Amaral, A. L., Thulio Marquez Cunha, Igor Moraes Mariano, and Guilherme Morais Puga (2024). Online and Face-to-Face Mat Pilates

Training for Long COVID-19 Patients: A Randomized Controlled Trial on Health Outcomes. *International Journal of Environmental Research and Public Health*, 21(10):1385. Place: Basel Publisher: MDPI AG.

[368] Richardson, L. M., Whitfield-Cargile, C. M., Cohen, N. D., Chamoun-Emanuelli, A. M., and Dockery, H. J. (2018). Effect of selective versus nonselective cyclooxygenase inhibitors on gastric ulceration scores and intestinal inflammation in horses. *Veterinary surgery*, 47(6):784–791.

[369] Riddell, D., Harron, R., Hildyard, J., Wells, D., and Piercy, R. (2024). Evaluation of a six-minute walk test in the DE50-MD canine model of Duchenne muscular dystrophy and its effect on blood-borne biomarkers. *Wellcome Open Research*, 9:681.

[370] Riddell, D. O., Hildyard, J. C. W., Harron, R. C. M., Hornby, N. L., Wells, D. J., and Piercy, R. J. (2022a). Serum inflammatory cytokines as disease biomarkers in the DE50-MD dog model of Duchenne muscular dystrophy. *Disease Models & Mechanisms*, 15(12):dmm049394.

[371] Riddell, D. O., Hildyard, J. C. W., Harron, R. C. M., Wells, D. J., and Piercy, R. J. (2022b). Longitudinal assessment of blood-borne musculoskeletal disease biomarkers in the DE50-MD dog model of Duchenne muscular dystrophy. *Wellcome Open Research*, 6:354. Type: article.

[372] Ringham, B. M., Kreidler, S. M., Muller, K. E., and Glueck, D. H. (2016). Multivariate test power approximations for balanced linear mixed models in studies with missing data. *Statistics in medicine*, 35(17):2921–2937.

[373] Rizvi, A., Wasfi, R., Enns, A., and Kristjansson, E. (2021). The impact of novel and traditional food bank approaches on food insecurity: a longitudinal study in Ottawa, Canada. *BMC Public Health*, 21:1–16. Place: London Publisher: BioMed Central.

[374] Roch, M., Morin, M., and Gaudreault, N. (2022). Immediate Effect of Dry Needling on the Viscoelastic Properties of a Trigger Point on the Infraspinatus Muscle Measured with MyotonPRO. *Physiotherapy Canada*, 74(3):232–239. Publisher: University of Toronto Press.

[375] Rodriguez, K. M., Krishnan, C., and Palmieri-Smith, R. M. (2024a). Number of conditioning trials, but not stimulus intensity, influences operant conditioning of brain responses after total knee arthroplasty. *Knee Surgery, Sports Traumatology, Arthroscopy*, n/a(n/a). _eprint: https://onlinelibrary.wiley.com/doi/pdf/10.1002/ksa.12480.

[376] Rodriguez, K. M., Moon, J., Krishnan, C., and Palmieri-Smith, R. M. (2024b). Conditioning of Motor Evoked Responses After Anterior Cruciate Ligament Reconstruction: Effects of Stimulus Intensity. *Sports Health*, page 19417381241257258.

[377] Rogers, M., Coates, A., Huggins, C. E., Dorrian, J., Clark, A. B., Davis, C., Leung, G. K., Davis, R., Phoi, Y. Y., Kellow, N. J., Iacovou, M., Yates, C. L., Banks, S., Sletten, T. L., and Bonham, M. P. (2022). Study protocol for the Shifting Weight using Intermittent Fasting in night shift workers (SWIFt) study: a three-arm randomised controlled trial comparing three weight loss strategies in night shift workers with obesity. *BMJ Open*, 12(4):e060520. Publisher: British Medical Journal Publishing Group Section: Nutrition and metabolism.

[378] Roh, Y. S. and Kim, S. S. (2015). Integrating problem-based learning and simulation effects on student motivation and life skills. *CIN: Computers, Informatics, Nursing*, 33(7):278–284. WOS:000369628500002.

[379] Romano, M., Hudd, T., Huppert, J. D., Reimer, S. G., and Moscovitch, D. A. (2021). Imagery Rescripting of Painful Memories in Social Anxiety Disorder: A Qualitative Analysis of Needs Fulfillment and Memory Updating. *Cognitive Therapy and Research*, 45(5):902–917.

[380] Romano, M., Moscovitch, D. A., Huppert, J. D., Reimer, S. G., and Moscovitch, M. (2020). The effects of imagery rescripting on memory outcomes in social anxiety disorder. *Journal of Anxiety Disorders*, 69:102169. WOS:000509005800006.

[381] Ross, W. and Arfini, S. (2024). Impasse-Driven problem solving: The multidimensional nature of feeling stuck. *Cognition*, 246:105746.

[382] Rusz, D., Erik Bijleveld, and Kompier, M. A. J. (2018). Reward-associated distractors can harm cognitive performance. *PLOS ONE*, 13(10). Place: San Francisco Publisher: Public Library of Science.

[383] Ryan, A., Casola, E., Fitzpatrick, C., and Knodler, M. (2019). Flashing yellow arrows for right turn applications: A driving simulator study and static evaluation analysis. *Transportation Research Part F: Traffic Psychology and Behaviour*, 66:324–338.

[384] Ryan, A., Fitzpatrick, C., Christofa, E., and Knodler, M. (2020). Driver performance due to small unmanned aerial system applications in the vicinity of roadways. *Transportation Research Part F-Traffic Psychology and Behaviour*, 68:118–131. Place: Oxford Publisher: Elsevier Sci Ltd WOS:000514015900010.

[385] Salamanca-Sanabria, A., Richards, D., Timulak, L., Castro-Camacho, L., Mojica-Perilla, M., and Parra-Villa, Y. (2018). Assessing the efficacy of a culturally adapted cognitive behavioural internet-delivered treatment for depression: protocol for a randomised controlled trial. *Bmc Psychiatry*, 18:53. WOS:000426372400002.

[386] Salihu, D., Wong, E. M. L., and Kwan, R. Y. C. (2021). Effects of an African Circle Dance Programme on Internally Displaced Persons with Depressive Symptoms: A Quasi-Experimental Study. *International Journal of Environmental Research and Public Health*, 18(2).

[387] Salzano, S., Zappullo, I., Senese, V. P., Conson, M., Citro, A., Cecere, R., Mikulincer, M., and Shaver, P. R. (2024). A 'Problematic' Pattern of Power-system Functioning and its Association with psychopathological Symptoms. *Psychological Reports*, page 332941241284063.

[388] Samad, M. D., Diawara, N., Bobzien, J. L., Taylor, C. M., Harrington, J. W., and Iftekharuddin, K. M. (2019). A pilot study to identify autism related traits in spontaneous facial actions using computer vision. *Research in Autism Spectrum Disorders*, 65:14–24. WOS:000474504900002.

[389] Sanders, N., Choo, S., and Nam, C. S. (2020). The eeg cookbook: a practical guide to neuroergonomics research. In Nam, C. S., editor, *Neuroergonomics: Principles and Practice*, Cognitive Science and Technology, pages 33–51. Springer International Publishing, Cham.

[390] Saquib, N., Al Sarraj, A. N., Oubaied, B. T. Z., Rajab, A. M., Agha, B. N., Hossain, J., Almazrou, A., and Saquib, J. (2021). Gain in growth after surgical repair of congenital heart disease among children with Down syndrome. *American Journal of Medical Genetics. Part A.*

[391] Sauder, K. A., Gamalski, K., DeRoeck, J., Vasquez, F. P., Dabelea, D., Glueck, D. H., Catenacci, V. A., Fabbri, S., and Ritchie, N. D. (2023). A pre-conception clinical trial to reduce intergenerational obesity and diabetes risks: The NDPP-NextGen trial protocol. *Contemporary Clinical Trials*, 133:107305.

[392] Savino, M., Guida, C. C., Nardella, M., Murgo, E., Augello, B., Merla, G., De Cosmo, S., Savino, A. F., Tarquini, R., Cei, F., Aucella, F., and Mazzoccoli, G. (2022). Circadian Genes Expression Patterns in Disorders Due to Enzyme Deficiencies in the Heme Biosynthetic Pathway. *Biomedicines*, 10(12):3198.

[393] Schiller, J. (2017). Effects of acupuncture on quality of life and pain in patients with osteoporosis-a pilot randomized controlled trial: reply to comments by Moran et al. *Archives of Osteoporosis*, 12(1):9. WOS:000394177000009.

[394] Schober, P. and Vetter, T. R. (2018). Repeated measures designs and analysis of longitudinal data: If at first you do not succeed-try, try again. *Anesthesia and Analgesia*, 127(2):569–575. WOS:000439308000056.

[395] Schuetz, I., Baltaretu, B. R., and Fiehler, K. (2024). Where was this thing again? Evaluating methods to indicate remembered object positions in virtual reality. *Journal of Vision*, 24(7):10.

[396] Schulz, P., Alexandrovsky, D., Putze, F., Malaka, R., and Schoening, J. (2019). The role of physical props in VR climbing environments. In *CHI '19: Proceedings of the 2019 CHI Conference on Human Factors in Computing Systems*, volume Paper 183, pages 1–13, New York. Assoc Computing Machinery. WOS:000474467902034.

[397] Scott, I. C., Rijsdijk, F., Walker, J., Quist, J., Spain, S. L., Tan, R., Steer, S., Okada, Y., Raychaudhuri, S., Cope, A. P., and Lewis, C. M. (2015). Do genetic susceptibility variants associate with disease severity in early active rheumatoid arthritis? *Journal of Rheumatology*, 42(7):1131–1140. WOS:000357280700013.

[398] Scoubeau, C., Klass, M., Celie, B., Godefroid, C., Cnop, M., and Faoro, V. (2024). Health-related fitness benefits following concurrent high-intensity interval training and resistance training in patients with type-1 diabetes or type-2 diabetes. *Frontiers in Physiology*, 15:1466148.

[399] Sermeus, L. A., Schepens, T., Hans, G. H., Morrison, S. G., Wouters, K., Breebaart, M. B., Smitz, C. J., and Vercauteren, M. P. (2019). A low dose of three local anesthetic solutions for interscalene blockade tested by thermal quantitative sensory testing: a randomized controlled trial. *Journal of clinical monitoring and computing*, 33(2):307–316.

[400] Setti, A., Braga, D., Guilherme, P., Iaconelli, A., and Borges, E. (2022). High oocyte immaturity rates affect embryo morphokinetics: lessons of time-lapse imaging system. *Reproductive BioMedicine Online*, 45(4):652–660.

[401] Setti, A. S., Braga, D. P. d. A. F., Guilherme, P., Provenza, R., Iaconelli, A., and Borges, E. (2021). Morphokinetic parameter comparison between embryos from couples with high or low sperm DNA fragmentation index. *F&S Science*, 2(4):345–354.

[402] Shaham, G. and Aviezer, H. (2022). Automatic facial reactions to emotional body expressions are not driven by emotional experience. *Emotion*, 22(4):641–652. Place: Washington Publisher: American Psychological Association, American Psychological Association.

[403] Shaham, G., Mortillaro, M., and Aviezer, H. (2020). Automatic facial reactions to facial, body, and vocal expressions: A stimulus-response compatibility study. *Psychophysiology*, page e13684. Place: Hoboken Publisher: Wiley WOS:000573614900001.

[404] Shenkman, E., Muller, K., Vogel, B., Nixon, S. J., Wagenaar, A. C., Case, K., Guo, Y., Wegman, M., Aric, J., and Stoner, D. (2015). The wellness incentives and navigation project: design and methods. *Bmc Health Services Research*, 15:579. WOS:000367222500002.

[405] Simonelli, N., Bolgeo, T., Iovino, P., Di Matteo, R., Maconi, A., and Vellone, E. (2023). Self-care in coronary heart disease patient and caregiver dyads (HEARTS-IN-DYADS)—Protocol of a multicenter longitudinal study. *Research in Nursing & Health*, 46(1):37–47. _eprint: https://onlinelibrary.wiley.com/doi/pdf/10.1002/nur.22286.

[406] Slade, K., Beat, A., Taylor, J., Plack, C. J., and Nuttall, H. E. (2023). The effect of motor resource suppression on speech perception in noise in younger and older listeners: An online study. *Psychonomic Bulletin & Review*.

[407] Smelson, D. A., Yakovchenko, V., Bruzios, K. E., Byrne, T., Mccullough, M., Shaffer, P. M., and Elwy, A. R. (2025). Testing implementation support strategies to facilitate an evidence-based substance use and mental health care intervention in veterans treatment courts: A hybrid type III trial protocol. *The European Journal of Psychiatry*, 39(1):100282.

[408] Smith, D., Fairweather-Schmidt, A. K., Pols, R., Harvey, P., and Battersby, M. (2018). Exploring patterns of change processes over distinct in-treatment phases of cognitive and exposure therapies for electronic gaming machine problem gamblers. *Behaviour Change*, 35(4):228–243. WOS:000451513500004.

[409] Smith, K. B., Zdaniuk, B., Ramachandran, S. O., and Brotto, L. A. (2022). A longitudinal case-control analysis of pain symptoms, fear of childbirth, and psychological well-being during pregnancy and postpartum among individuals with vulvodynia. *Midwifery*, 114:103467.

[410] Soldevila-Domenech, N., Boronat, A., Mateus, J., Diaz-Pellicer, P., Matilla, I., Perez-Otero, M., Aldea-Perona, A., and de la Torre, R. (2019). Generation of the antioxidant hydroxytyrosol from tyrosol present in beer and red wine in a randomized clinical trial. *Nutrients*, 11(9):2241. WOS:000487964600178.

[411] Sommer, M., Ritzhaupt, A. D., Muller, K. E., and Glueck, D. H. (2019). Transformation of a face-to-face workshop into a Massive Open Online Course (MOOC): A design and development case. *Journal of Formative Design in Learning*, 3(2):97–110.

[412] Sorrento, G., Archambault, P., and Fung, J. (2018). Adaptation and post-Adaptation effects of haptic forces on locomotion in healthy young adults. *Journal of NeuroEngineering and Rehabilitation*, 15.

[413] Sowden, S., Schuster, B. A., Keating, C. T., Fraser, D. S., and Cook, J. L. (2021). The role of movement kinematics in facial emotion expression production and recognition. *Emotion (Washington, D.C.)*.

[414] Spampatti, T., Hahnel, U. J. J., Trutnevyte, E., and Brosch, T. (2022). Short and long-term dominance of negative information in shaping public energy perceptions: The case of shallow geothermal systems. *Energy Policy*, 167:113070.

[415] St. Germain, L., Williams, A., Balbaa, N., Poskus, A., Leshchyshen, O., Lohse, K. R., and Carter, M. J. (2022). Increased perceptions of autonomy through choice fail to enhance motor skill retention. *Journal of Experimental Psychology: Human Perception and Performance*, 48(4):370–379. Place: Washington Publisher: American Psychological Association, American Psychological Association.

[416] Standen, E., Rothman, A., and Mann, T. (2025). Weight loss advice from a healthcare provider is motivating, but it is also stigmatizing: an experimental, scenario-based approach. *Annals of Behavioral Medicine*, 59.

[417] Stark, M., Tietz, R., Gattinger, H., Hantikainen, V., and Ott, S. (2017). Effects of a mobility monitoring system on the cost of care in relation to reimbursement at Swiss nursing homes: learnings from a randomized controlled trial. *Health economics review*, 7(1):43.

[418] Starrett, M. J., Huffman, D. J., and Ekstrom, A. D. (2022). Combining egoformative and alloformative cues in a novel tabletop navigation task. *Psychological Research*.

[419] Stecher, C., Sullivan, M., and Huberty, J. (2021). Using Personalized Anchors to Establish Routine Meditation Practice With a Mobile App: Randomized Controlled Trial. *JMIR mHealth and uHealth*, 9(12). Place: Toronto Publisher: JMIR Publications.

[420] Steiner, H. E., Patterson, H. K., Giles, J. B., and Karnes, J. H. (2022). Bringing pharmacomicrobiomics to the clinic through well-designed studies. *Clinical and Translational Science*, 15(10):2303–2315.

[421] Stenling, A., Quensell, J., Kaur, N., and Machado, L. (2024). Stair Climbing Improves Cognitive Switching Performance and Mood in Healthy Young Adults: A Randomized Controlled Crossover Trial. *Journal of Cognitive Enhancement*, 8(3):191–205.

[422] Stolt, E., Olsen, T., Elshorbagy, A., Kožich, V., van Greevenbroek, M., Øvrebø, B., Thoresen, M., Refsum, H., Retterstøl, K., and Vinknes, K. J. (2021). Sulfur amino acid restriction, energy metabolism and obesity: a study protocol of an 8-week randomized controlled dietary intervention with whole foods and amino acid supplements. *Journal of Translational Medicine*, 19(1):153.

[423] Stote, K., Corkum, A., Sweeney, M., Shakerley, N., Kean, T., and Gottschall-Pass, K. (2019). Postprandial effects of blueberry (Vaccinium angustifolium) consumption on glucose metabolism, gastrointestinal hormone response, and perceived appetite in healthy adults: A randomized, placebo-controlled crossover trial. *Nutrients*, 11(1):202.

[424] Stratakis, N., Gielen, M., Margetaki, K., Godschalk, R. W., van der Wurff, I., Rouschop, S., Ibrahim, A., Antoniou, E., Chatzi, L., de Groot, R. H. M., and Zeegers, M. P. (2017). Polyunsaturated fatty acid levels at birth and child-to-adult growth: Results from the MEFAB cohort. *Prostaglandins Leukotrienes and Essential Fatty Acids*, 126:72–78. WOS:000414107600010.

[425] Strohacker, K., Galárraga, O., Emerson, J., Fricchione, S. R., Lohse, M., and Williams, D. M. (2015). Impact of small monetary incentives on exercise in university students. *American journal of health behavior*, 39(6):779–786.

[426] Stuppy-Sullivan, A. M., Buckholtz, J. W., and Baskin-Sommers, A. (2020). Aberrant cost-benefit integration during effort-based decision making relates to severity of substance use disorders. *Clinical Psychological Science*, 8(1):155–168. WOS:000496386200001.

[427] Sturm, V. E., Datta, S., Roy, A. R. K., Sible, I. J., Kosik, E. L., Veziris, C. R., Chow, T. E., Morris, N. A., Neuhaus, J., Kramer, J. H., Miller, B. L., Holley, S. R., and Keltner, D. (2020a). Big smile, small self: Awe walks promote prosocial positive emotions in older adults. *Emotion (Washington, D.C.)*.

[428] Sturm, V. E., Roy, A. R. K., Datta, S., Wang, C., Sible, I. J., Holley, S. R., Watson, C., Palser, E. R., Morris, N. A., Battistella, G., Rah, E., Meyer, M., Pakvasa, M., Mandelli, M. L., Deleon, J., Hoeft, F., Caverzasi, E., Miller, Z. A., Shapiro, K. A., Hendren, R., Miller, B. L., and Gorno-Tempini, M. L. (2020b). Enhanced visceromotor emotional reactivity in dyslexia and its relation to salience network connectivity. *Cortex*.

[429] Suessenbach, P., Rees, J., and Gollwitzer, M. (2019). When the going gets tough, individualizers get going: On the relationship between moral foundations and prosociality. *Personality and Individual Differences*, 136:122–131. WOS:000447816600013.

[430] Summers, S. J., Chipchase, L. S., Hirata, R., Graven-Nielsen, T., Cavaleri, R., and Schabrun, S. M. (2019). Motor adaptation varies between individuals in the transition to sustained pain. *Pain*, 160(9):2115–2125.

[431] Sysko, R., Michaelides, A., Costello, K., Herron, D. M., and Hildebrandt, T. (2022). An Initial Test of the Efficacy of a Digital Health Intervention for Bariatric Surgery Candidates. *Obesity Surgery*.

[432] Szynkiewicz, S. H., Nobriga, C., O'Donoghue, C. R., Becerra, B. J., and LaForge, G. (2019). Motor imagery practice and increased tongue strength: A case series feasibility report. *Journal of Speech Language and Hearing Research*, 62(6):1676–1684. WOS:000472020700005.

[433] Tarabbia, F., Bertozzi, F., Allevi, F., Giovanni Dell'Aversana Orabona, Cupello, S., Dolci, C., Zago, M., Sforza, C., and Biglioli, F. (2022). Smile Reanimation with Masseteric-to-Facial Nerve Transfer plus Cross-Face Nerve Grafting in Patients with Segmental Midface Paresis: 3D Retrospective Quantitative Evaluation. *Symmetry*, 14(12):2570. Place: Basel Publisher: MDPI AG.

[434] Tavares, V. D. d. O., Schuch, F. B., de Sousa, G. M., Hallgren, M., Oliveira Neto, L., Cabral, D. A. R., Nóbrega de Almeida, R., Barbosa, D. C., de Almeida, V. R. N., Tinoco, H., Lira, R. A., Hallak, J. E., Arcoverde, E., Cuthbert, C., Patten, S., and Galvão-Coelho, N. L. (2025). Effectiveness of an affect-adjusted, supervised, multimodal, online

and home-based exercise group protocol for major depression: A randomized controlled trial. *Psychology of Sport and Exercise*, 76:102729.

[435] Teixeira-Machado, L., Arida, R. M., Ziebold, C., Barboza, A. B., Ribeiro, L., Teles, M. C., Rodrigues da Cunha Azevedo, G., Silvestre de Paula, C., Lowenthal, R., and Mari de Jesus, J. (2022). A pilot randomized controlled clinical trial of dance practice for functionality in autistic children and adolescent with all levels of need support. *Complementary Therapies in Clinical Practice*, 49:101650.

[436] Telepak, L. C. (2015). *Sleep disturbance as a predictor for psychosocial symptoms following surgery for gynecologic cancer: A focus on intraindividual variability.* Ph.D., University of Florida, United States – Florida. ISBN: 9781369420913.

[437] Thai, H., Davis, C., Mahboob, W., Perry, S., Adams, A., and Goldfield, G. (2023). Reducing Social Media Use Improves Appearance and Weight Esteem in Youth With Emotional Distress. *Psychology of Popular Media*.

[438] Thai, H., Davis, C., Stewart, N., Gunnell, K., and Goldfield, G. (2021). The Effects of Reducing Social Media Use on Body Esteem Among Transitional-Aged Youth. *Journal of Social and Clinical Psychology*, 40:481–507.

[439] Thakurdesai, P., Deshpande, P., Desai, N., Mathad, P., Rani, S., and Raje, D. (2024). A Double-blind, Randomized Controlled Study of Triterpenoids based Standardized Gotu Kola Leaves Extract in the Patients with Tension Type Headache. *Pharmacognosy Journal*, 16(6):1238–1251.

[440] Theeuwes, J., van Doorn, J., and van Moorselaar, D. (2025). Suppression of fear-conditioned stimuli. *Emotion (Washington, D.C.)*.

[441] Thoefner, M. S., Skovgaard, L. T., McEvoy, F. J., Berendt, M., and Bjerrum, O. J. (2020). Pregabalin alleviates clinical signs of syringomyelia-related central neuropathic pain in Cavalier King Charles Spaniel dogs: a randomized controlled trial. *Veterinary Anaesthesia and Analgesia*, 47(2):238–248. Place: Amsterdam Publisher: Elsevier WOS:000516848100013.

[442] Tiong, M. K., Cai, M. M. X., Toussaint, N. D., Tan, S.-J., Pasch, A., and Smith, E. R. (2022). Effect of nutritional calcium and phosphate loading on calciprotein particle kinetics in adults with normal and impaired kidney function. *Scientific Reports*, 12(1):7358. Number: 1 Publisher: Nature Publishing Group.

[443] Tiong, M. K., Smith, E. R., Toussaint, N. D., Al-Khayyat, H. F., and Holt, S. G. (2020). Reduction Of Calciprotein Particles In Adults Receiving Infliximab For Chronic Inflammatory Disease. *JBMR Plus*, n/a(n/a):e10497. _eprint: https://asbmr.onlinelibrary.wiley.com/doi/pdf/10.1002/jbm4.10497.

[444] Tkacheva, L., Flaksman, M., Nasledov, A., Sedelkina, Y., and Lavitskaya, Y. (2021). Iconicity and Second Language Visual Perception: A Psycholinguistic Study of English Imitative Words at Different De-Iconization Stages. *Mathematics*, 9:1331.

[445] Tost, M., González-Rodríguez, A., Aguayo, R., Álvarez, A., Montalvo, I., Barbero, J. D., Gabernet, R., Izquierdo, E., Merodio, I., Monreal, J. A., Palao, D., and Labad, J. (2023). Switching from risperidone to paliperidone palmitate in schizophrenia: Changes

in social functioning and cognitive performance. *Progress in Neuro-Psychopharmacology & Biological Psychiatry*, 120:110619.

[446] Toy, S., McKay, R., Eilert, R., and Sandall, J. (2019). Effective and Feasible Simulation-Based Procedural Training for Medical Students: Instructional Video-Guided Deliberate Practice Versus Training with Expert Feedback. *Medical Science Educator*, 29(1):35–39.

[447] Trindade, I. A., Soares, A., Skvarc, D., Carreiras, D., Pereira, J., Lourenço, Ó., Sampaio, F., de Sousa, B., Martins, T. C., Boaventura, P., Marta-Simões, J., Moreira, H., and Mind Project Team (2025). Efficacy and cost-effectiveness of an ACT and compassion-based intervention for women with breast cancer: study protocol of two randomised controlled trials {1}. *Trials*, 26(1):5.

[448] Tueller, S., Ramirez, D., Cance, J. D., Ye, A., Wheeler, A. C., Fan, Z., Hornik, C., and Ridenour, T. A. (2022). Power analysis for idiographic (within-subject) clinical trials: Implications for treatments of rare conditions and precision medicine. *Behavior Research Methods*, pages 1–25.

[449] Tulva, K., Pirajev, A., Zeb, A., Aksoy, A. E., Bello, A., Lee, B., Guðjónsson, B. F., Helgadottir, S. B., Jagomäe, T., García-Llorca, A., Eysteinsson, T., Jürgenson, M., Plaas, M., Vasar, E., Kaasik, A., and Hickey, M. A. (2025). Early trigeminal and sensory impairment and lysosomal dysfunction in accurate models of Wolfram syndrome. *Experimental Neurology*, 385:115099.

[450] Turkakin, E., Akbiyik, S., Akyol, B., Gurdere, C., Cakmak, Y. O., and Balci, F. (2018). Differential bilateral primary motor cortex tdcs fails to modulate choice bias and readiness in perceptual decision making. *Frontiers in Neuroscience*, 12:410. WOS:000435499200001.

[451] Türkoğlu, Ş. A., Bolac, E. S., Yildiz, S., Kalaycioglu, O., and Yildiz, N. (2021). Presynaptic inhibition in restless legs syndrome. *The International Journal of Neuroscience*, 131(3):213–219.

[452] Uribe, F. A. R., Favacho, M. F. M., Moura, P. M. N., Patiño, D. M. C., and da Silva Pedroso, J. (2023). Effectiveness of an app-based intervention to improve well-being through cultivating positive thinking and positive emotions in an adult sample: study protocol for a randomized controlled trial. *Frontiers in Psychology*, 14.

[453] Vagenas, D. and Totsika, V. (2018). Modelling correlated data: Multilevel models and generalized estimating equations and their use with data from research in developmental disabilities. *Research in Developmental Disabilities*, 81:1–11. WOS:000445443100001.

[454] Valderrama-Rios, M. C., Sánchez, R., and Sanabria, M. (2024). Psychometric properties of the Kidney Disease Quality of Life short form 36 (KDQOL-36) scale for the assessment of quality of life in Colombian patients with chronic kidney disease on dialysis. *International Urology and Nephrology*, 56(7):2337–2350.

[455] Van Asten, F., Van Middendorp, H., Verkerk, S., Breukink, M. B., Lomme, R. M. L. M., Hoyng, C. B., Evers, A. W., and Klevering, B. J. (2015). Are intravitreal injections with ultrathin 33-G needles Less painful than the commonly used 30-G needles? *Retina-the Journal of Retinal and Vitreous Diseases*, 35(9):1778–1785. WOS:000361196600010.

[456] Van den Steen, L., Baudelet, M., Tomassen, P., Bonte, K., De Bodt, M., and Van Nuffelen, G. (2020). The effect of tongue-strengthening exercises on tongue strength and swallowing-related parameters in chronic radiation-associated dysphagia. *Head and Neck-Journal for the Sciences and Specialties of the Head and Neck*, 42(9):2298–2307. Place: Hoboken Publisher: Wiley WOS:000529630300001.

[457] van der Linden, N., Klinkenberg, L. J. J., Leenders, M., Tieland, M., Verdijk, L. B., Niens, M., van Suijlen, J. D. E., de Groot, L. C. P. G. M., Bekers, O., van Loon, L. J. C., van Dieijen-Visser, M. P., and Meex, S. J. R. (2015). The effect of exercise training on the course of cardiac troponin T and I levels: three independent training studies. *Scientific Reports*, 5:18320. WOS:000366482400001.

[458] van der Linden, N., Tieland, M., Klinkenberg, L. J. J., Verdijk, L. B., de Groot, L. C. P. G. M., van Loon, L. J. C., van Dieijen-Visser, M. P., and Meex, S. J. R. (2014). The effect of a six-month resistance-type exercise training program on the course of high sensitive cardiac troponin T levels in (pre)frail elderly. *International Journal of Cardiology*, 175(2):374–375. WOS:000340249400034.

[459] van der Rhee, M., Oosterman, J. E., Wopereis, S., Gijsbertus T. J. van der Horst, Chaves, I., Dollé, M. E. T., Burdorf, A., Linda W. M. van Kerkhof, and Heidi M. Lammers-Van der Holst (2024). Personalized sleep and nutritional strategies to combat adverse effects of night shift work: a controlled intervention protocol. *BMC Public Health*, 24:1–10. Place: London Publisher: BioMed Central.

[460] Van Nuffelen, G., Van den Steen, L., Vanderveken, O., Specenier, P., Van Laer, C., Van Rompaey, D., Guns, C., Mariën, S., Peeters, M., Van de Heyning, P., Vanderwegen, J., and De Bodt, M. (2015). Study protocol for a randomized controlled trial: tongue strengthening exercises in head and neck cancer patients, does exercise load matter? *Trials*, 16:395.

[461] Veerman, L. K. M., Willemen, A. M., Derks, S. D. M., Brouwer-van Dijken, A. A. J., and Sterkenburg, P. S. (2023). The effectiveness of the serious game "Broodles" for siblings of children with intellectual disabilities and/or visual impairment: study protocol for a randomized controlled trial. *Trials*, 24(1):1–18. Number: 1 Publisher: BioMed Central.

[462] Veerman, L. K. M., Willemen, A. M., Derks, S. D. M., Brouwer-van Dijken, A. A. J., and Sterkenburg, P. S. (2025). Supporting young siblings of children with intellectual disabilities and/or visual impairments with the serious game 'Broodles': A mixed methods randomized controlled trial. *Research in Developmental Disabilities*, 161:104996.

[463] Vilar, J. M., Manera, M. E., Santana, A., Spinella, G., Rodriguez, O., Rubio, M., Carrillo, J. M., Sopena, J., and Batista, M. (2018). Effect of leukocyte-reduced platelet-rich plasma on osteoarthritis caused by cranial cruciate ligament rupture: A canine gait analysis model. *PLOS ONE*, 13(3). Place: San Francisco Publisher: Public Library of Science.

[464] Viniol, C., Galetke, W., Woehrle, H., Nilius, G., Schöbel, C., Randerath, W., Leiter, J., Canisius, S., and Schneider, H. (2025). Clinical validation of a wireless patch-based polysomnography system. *Journal of Clinical Sleep Medicine*, 0(0):jcsm.11524. Publisher: American Academy of Sleep Medicine.

[465] Viruega, H., Gaillard, I., Briatte, L., and Gaviria, M. (2020). Inter-Day Reliability and Changes of Surface Electromyography on Two Postural Muscles Throughout 12 Weeks of Hippotherapy on Patients with Cerebral Palsy: A Pilot Study. *Brain Sciences*, 10(5):281. Place: Basel Publisher: MDPI AG.

[466] Viruega, H., Gaillard, I., Carr, J., Greenwood, B., and Gaviria, M. (2019). Short- and Mid-Term Improvement of Postural Balance after a Neurorehabilitation Program via Hippotherapy in Patients with Sensorimotor Impairment after Cerebral Palsy: A Preliminary Kinetic Approach. *Brain Sciences*, 9(10):261. Place: Basel Publisher: MDPI AG.

[467] Vispoel, W. P., Morris, C. A., and Clough, S. J. (2018). Interchangeability of results from computerized and traditional administration of the BIDR: Convenience can match reality. *Journal of personality assessment*, pages 1–16.

[468] Wagstaff, C. R. D. (2014). Emotion regulation and sport performance. *Journal of Sport & Exercise Psychology*, 36(4):401–412. WOS:000342476300008.

[469] Wang, G., Wang, W., Wang, Z., Huang, S., Liu, Y., and Ming, D. (2025). The sixth finger illusion induced by palm outside stroking shows stable ownership and independence. *Scientific Reports*, 15(1):11447. Publisher: Nature Publishing Group.

[470] Wang, P., Yang, L., Liu, C., Wei, X., Yang, X., Zhou, Y., Jiang, H., Lei, Z., Reinhardt, J. D., and He, C. (2016). Effects of whole body vibration exercise associated with quadriceps resistance exercise on functioning and quality of life in patients with knee osteoarthritis: a randomized controlled trial. *Clinical Rehabilitation*, 30(11):1074–1087. WOS:000386689200005.

[471] Wang, P.-W., Huang-Chi, L., Chia-Nan Yen, Yi-Chun Yeh, Chih-Yao Hsu, Kuan-Sheng, C., Hsun-Cheng Chang, Hung-Chi, W., and Cheng-Fang, Y. (2015). Comparison of outcomes after 3-month methadone maintenance treatment between heroin users with and without HIV infection: a 3-month follow-up study. *Harm Reduction Journal*, 12:1–7. Place: London Publisher: BioMed Central.

[472] Wang, Y.-L., Wu, W.-X., Yang, C.-C., Huang, S.-M., Chang, C.-C., Li, C.-R., Chiang, S.-L., and Chen, Y.-J. (2024). Heart rate variability biofeedback enhances cognitive, motor, psychological, and autonomic functions in post-stroke rehabilitation. *International Journal of Psychophysiology*, 203:112411.

[473] Watson, K., Hatcher, D., and Good, A. (2019). A randomised controlled trial of Lavender (Lavandula Angustifolia) and Lemon Balm (Melissa Officinalis) essential oils for the treatment of agitated behaviour in older people with and without dementia. *Complementary Therapies in Medicine*, 42:366–373.

[474] Weber, M., Giacomin, J., Malizia, A., Skrypchuk, L., Gkatzidou, V., and Mouzakitis, A. (2019). Investigation of the dependency of the drivers' emotional experience on different road types and driving conditions. *Transportation Research Part F-Traffic Psychology and Behaviour*, 65:107–120. WOS:000491217600010.

[475] Wertheim, B., Aarts, E. E., de Roos, C., and van Rood, Y. R. (2023). The effect of eye movement desensitization and reprocessing (EMDR) on abdominal pain in patients

with irritable bowel syndrome (IBS): a study protocol for a randomized controlled trial (EMDR4IBS). *Trials*, 24(1):785. Place: London Publisher: BioMed Central.

[476] Wiebe, M., Granata, N., and Lane, J. D. (2022). Children's attributions of knowledge and trustworthiness to persons with disabilities. *Cognitive Development*, 61:101143.

[477] Wiercioch-Kuzianik, K. and Babel, P. (2019). Color hurts. The effect of color on pain perception. *Pain Medicine*, 20(10):1955–1962. WOS:000498052000011.

[478] Wittekind, C. E., Bierbrodt, J., Luedecke, D., Feist, A., Hand, I., and Moritz, S. (2019). Cognitive bias modification in problem and pathological gambling using a web-based approach-avoidance task: A pilot trial. *Psychiatry Research*, 272:171–181. WOS:000460994400026.

[479] Wolf, T. G., Ernst-Jürgen Otterbach, Zeyer, O., Ralf Friedrich Wagner, Crnić, T., Ilhan, D., and Campus, G. (2021). Influence of Oral Health Care Systems on Future Career Environment of Dental Students in Europe. *International Journal of Environmental Research and Public Health*, 18(16):8292. Place: Basel Publisher: MDPI AG.

[480] Wood, J., Quinn-Nilas, C., Milhausen, R., Desmarais, S., Muise, A., and Sakaluk, J. (2021). A dyadic examination of self-determined sexual motives, need fulfillment, and relational outcomes among consensually non-monogamous partners. *PLOS ONE*, 16(2):e0247001.

[481] Woody, M., Price, R., Amole, M., Hutchinson, E., Allen, K., and Silk, J. (2020). Using Mobile Eye Tracking Technology to Examine Adolescent Daughters' Attention to Maternal Affect during a Conflict Discussion. *Developmental Psychobiology*.

[482] Woolman, M., Katz, L., Tata, A., Basu, S. S., and Zarrine-Afsar, A. (2021). Breaking Through the Barrier. *Clinics in Laboratory Medicine*, 41(2):221–246.

[483] Wray, T. B., Chan, P. A., Klausner, J. D., Mena, L. A., Brock, J. B., Simpanen, E. M., Ward, L. M., and Chrysovalantis, S. (2020). eTest: a limited-interaction, longitudinal randomized controlled trial of a mobile health platform that enables real-time phone counseling after HIV self-testing among high-risk men who have sex with men. *Trials*, 21(1):654.

[484] Wu, J., Masuy, I., Biesiekierski, J. R., Fitzke, H. E., Parikh, C., Schofield, L., Shaikh, H., Bhagwanani, A., Aziz, Q., Taylor, S. A., Tack, J., and Van Oudenhove, L. (2022). Gut-brain axis dysfunction underlies FODMAP-induced symptom generation in irritable bowel syndrome. *Alimentary Pharmacology & Therapeutics*.

[485] Wych, J., Grayling, M. J., and Mander, A. P. (2019). Sample size re-estimation in crossover trials: application to the AIM HY-INFORM study. *Trials*, 20(1):665.

[486] Xiang, A., Xu, M., Liang, Y., Wei, J., and Liu, S. (2017). Immediate relief of herniated lumbar disc-related sciatica by ankle acupuncture: A study protocol for a randomized controlled clinical trial. *Medicine*, 96(51):e9191. WOS:000422992300048.

[487] Yang, T. and Bi, C. (2024). Examining the trajectory of meaning violation and its bidirectional relationship with perceived posttraumatic growth. *Stress and Health*, 40(4):e3394. _eprint: https://onlinelibrary.wiley.com/doi/pdf/10.1002/smi.3394.

[488] Yu, Q., Gratzke, C., Wang, Y., Herlemann, A., Sterr, C. M., Rutz, B., Ciotkowska, A., Wang, X., Strittmatter, F., and Stief, C. G. (2018). Inhibition of human prostate smooth muscle contraction by the LIM kinase inhibitors, SR7826 and LIMKi3. *British journal of pharmacology*, 175(11):2077–2096.

[489] Yuan, R.-Z., Li, K.-P., Wei, X.-L., Zheng, W., Ye, Y., Wang, M.-Y., Jiang, J.-T., and Wu, C.-Q. (2021). Effects of free range-of-motion upper limb exercise based on mirror therapy on shoulder function in patients after breast cancer surgery: study protocol for a randomized controlled trial. *Trials*, 22(1):815. Place: London Publisher: BioMed Central.

[490] Zahedi, A., Łuczak, A., and Sommer, W. (2020a). Modification of food preferences by posthypnotic suggestions: An event-related brain potential study. *Appetite*, 151:104713.

[491] Zahedi, A., Stürmer, B., and Sommer, W. (2020b). Can posthypnotic suggestions boost updating in working memory? Behavioral and ERP evidence. *Neuropsychologia*, 148:107632.

[492] Zanzer, Y. C., Plaza, M., Dougkas, A., Turner, C., Björck, I., and Östman, E. (2017). Polyphenol-rich spice-based beverages modulated postprandial early glycaemia, appetite and PYY after breakfast challenge in healthy subjects: A randomized, single blind, crossover study. *Journal of Functional Foods*, 35:574–583.

[493] Zanzer, Y. C., Plaza, M., Dougkas, A., Turner, C., and Östman, E. (2018). Black pepper-based beverage induced appetite-suppressing effects without altering postprandial glycaemia, gut and thyroid hormones or gastrointestinal well-being: a randomized crossover study in healthy subjects. *Food & Function*, 9(5):2774–2786.

[494] Zeng, P.-Y. and Yeh, S.-L. (2025). Exploring semantic expression disparities in intragenerational and intergenerational communication: A novel perspective on socioemotional selectivity theory. *Psychology and Aging*, pages No Pagination Specified–No Pagination Specified. Place: US Publisher: American Psychological Association.

[495] Zetterqvist, V., Grudin, R., Rickardsson, J., Wicksell, R. K., and Holmstrom, L. (2018). Acceptance-based behavioural treatment for insomnia in chronic pain: A clinical pilot study. *Journal of Contextual Behavioral Science*, 9:72–79. WOS:000441973400009.

[496] Zillioux, J. M., Corbett, S. T., Hunter, E., and Kern, N. G. (2018). Bladder scan accuracy in pediatric patients: Does patient position matter? *Journal of pediatric urology*, 14(5):438–444.

[497] Zsok, F., Fleischman, D. S., Borg, C., and Morrison, E. (2017). Disgust Trumps Lust: Women's Disgust and Attraction Towards Men Is Unaffected by Sexual Arousal. *Evolutionary Psychological Science*, 3(4):353–363.
