# Supplementary material for: Power and sample size for reversible linear mixed models with clustering and longitudinality: GLIMMPSE Version 3
Source: PLoS One. 2025 Sep 3;20(9):e0329712. doi: 10.1371/journal.pone.0329712 (PMC12407473; doi:10.1371/journal.pone.0329712)
Supplement: Attachment — Submitted filename: GLIMMPSE_PLOS_ONEResponseToReview.pdf [file pone.0329712.s004.pdf]

Response to review
PONE-D-24-57373
Power and Sample Size for Balanced Linear Mixed Models with Clustering and Longitudinality: GLIMMPSE 3

Dear Professor Ghosh,

Many thanks for managing the review and selecting the reviewers. We very much appreciate the suggestions from the reviewers, which we think have strongly improved the manuscript. Insertions appear marked in red in the manuscript. Deletions, which were minor, were not tracked. Below, reviewer comments appear numbered and in italics, with our responses in plain text. We excerpted and indented the revised text that was added to the manuscript here in the response to review, for the convenience of the reviewers.

Acknowledgments

We added the following statement to the acknowledgments.

> The funders had no role in the study design, data collection and analysis, decision to publish, or preparation of the manuscript.

Reviewer 1

1. *This is an important methodological contribution, with robust theoretical grounding and practical tools to help researchers design studies requiring linear mixed-model analyses.*

   Many thanks for your kind words.

2. <u>*Clarification of Reversibility Conditions*</u> *The manuscript emphasizes the importance of "reversibility" in mixed models but could benefit from more explicit statements or examples of when these conditions are (and are not) met in practice. For instance, while the conditions are described mathematically, it would help readers to see a short paragraph explaining typical pitfalls—e.g., when cluster sizes differ or data are missing at some time points (which breaks the assumption of complete data). Add subsection in Section 2 ("The Reversible General Linear Mixed Model") to give at least one real-world scenario in which reversibility fails and how that would impact power analysis.*

   This is a good idea. We added the following text to the manuscript.

   > Ringham *et al.* [28] gives examples of reversible linear mixed models. They describe a longitudinal study, a group-randomized trial, a three-level group-randomized trial, and a study with multivariate outcomes. In the longitudinal study, investigators plan to study the shape of the weight gain curve of women during pregnancy. Each woman is measured nine times on the first day of each month of gestation. If an intercept is used as the predictor, the coefficients of the model represent the average weight at each time point measured. In the group-randomized trial, investigators plan to randomize entire workplaces to one of two alcohol reduction programs. Each workplace has the same number of workers. The outcome is the self-reported amount of alcohol ingested by each worker in each workplace

in the week following the intervention. When indicator variables for the intervention group are used as predictors, the coefficients for the model represent the average intake across workplaces and workers for each intervention. In the study with multiple outcomes, three oral cancer biomarkers are measured for each participant. Some of the participants have diagnosed cancer of the oral cavity or pharynx (cases), and some have no diagnoses of oral cancer (controls). Using indicator variables for cases or controls yields model coefficients which are the average values of each biomarker for the cases and the controls.

In practice, in each of the designs described, missing data could occur. In the longitudinal study, women might not come in each month for measures, or might not receive one of the nine measurements on the day that they come in. In the group randomized trial, the clusters might be of different sizes. In the oral cancer study, some participants might have only one or two biomarkers measured, instead of all three. With missing data, or unequal cluster sizes, the models are no longer reversible. In each of these cases, power computed for a reversible model assuming no missing data will be higher than the true power.

GLIMMPSE provides power calculations for reversible mixed models with no missing data. Some rule of thumb approximations for missing data appear in the Discussion. For users who wish to use an analytic approach account for missing data, Kreidler *et al.* [17] reviews the literature for power and sample size calculations for designs with missing data, different cluster sizes, or both. In addition, Kreidler *et al.* [17] derives a non-central F approximation for the Wald test with Kenward and Roger [13] degrees of freedom, and provides an open-source package which allows power calculations.

3. <u>*Broadening Example Designs*</u> *The examples provided are highly relevant (and cover cluster-randomized, longitudinal, and multi-level designs). However, the text might explicitly mention other design types (e.g., crossover designs, stepped-wedge designs, or observational studies with repeated measures) and whether GLIMMPSE 3 can or cannot handle them. Suggested Revision: In Section 4 ("Examples"), add brief bullet points stating how the software might be used or not used for other popular designs (e.g., stepped-wedge trials).*

Thanks. In the Discussion, we added two sentences clarifying the scope of the software. The revised sentences follow, in their context in paragraphs.

> GLIMMPSE provides power and sample size calculations for a wide range of designs. While only randomized controlled trials are given in the examples, the package can also be used to compute power and sample size for observational studies, including those with longitudinal repeated measures, clustering, or both.
>
> GLIMMPSE has four current gaps in capability, which we plan to target in the future. GLIMMPSE does not provide calculations for randomized complete block designs, nor any design with randomization within the cluster. GLIMMPSE does not provide calculations which allow users to compare the effects on power and sample size of choosing multivariate rather than composite outcomes. GLIMMPSE does not provide capability to support power calculations for high dimensional data, including microbiome, epigenetic, genetic, and metabolomic data. Finally, GLIMMPSE does not allow accounting for unequal cluster sizes. We hope to add these capabilities in the future.

4. <u>*Covariance Structures and Alternatives*</u> *The authors introduce a Longford-based variance components approach. While this is well justified, readers may wonder how robust the method is under certain misspecifications (e.g., if the true covariance is a partially autoregressive correlation of order 1, AR(1), or if only partial nesting is present).Suggested Revision: Add a short statement in Section 3.1 or Section 3.2, clarifying how sensitive the method is to mild covariance misspecification. Cite any existing references (e.g., Pinheiro & Bates, 2000; Laird & Ware, 1982; or relevant chapters from Linear Model Theory by Muller & Stewart, 2006).*

   This is a very interesting question. Neither we, nor the references you suggested here have examined the effect on power calculations of misspecifying the population error variance in power calculations. Muller *et al.* [20] looked at the effect in data, rather than power analysis, of using both a different contrast, test, and variance.

   We considered adding this as a limitation in the discussion, but demurred. We think that this interesting question is outside the scope of a manuscript focused on describing power and sample size software. We hope to study this important question in future, more mathematically driven manuscripts.

5. <u>*Handling Missing Data*</u> *Although the authors note that the software handles designs under the assumption of complete data, many real-world studies encounter missingness. The authors mention that advanced methods exist (e.g., references to Josey et al., 2023), but the practical approach within GLIMMPSE 3 could be expanded. Suggested Revision: In the "Future Directions" or "Discussion" section, briefly note the limitations for missing data. Mention if future releases might incorporate approximations for missing data or reference existing guidance on incorporating dropout in sample size planning (e.g., by inflating sample size or adjusting design effect).*

   We have added a paragraph to the discussion section about our future plans. The revised paragraph follows.

   > GLIMMPSE has some limitations. First, the use of the reversible model means that all outcomes, predictors and covariates are assumed to have complete data. In practice, missing data occurs routinely. In the future, the hope is to incorporate into the software several power and sample size methods that can accommodate missing data, including those of Ringham *et al.* 2016 and Kreidler *et al.* 2021. Ringham *et al.* 2016 provided power calculations accounting for missing data for a modified test statistic due to Catellier and Muller 2000. Kreidler *et al.* 2021 gave power approximations for the Kenward and Roger 2009 test for the mixed model, which can also allow for missing data.

6. <u>*Software Usability and Maintenance*</u> *The authors highlight improvements to the interface and code refactoring in Python. A potential concern is ensuring long-term maintainability and stability (e.g., version control, bug tracking, frequency of releases). Suggested Revision: Add a brief paragraph in Section 3.2 clarifying the planned schedule for future updates or a statement about user/community involvement in the GitHub repository (e.g., encouraging pull requests).*

   We share your concern for long-term maintainability and stability. We do not currently have a schedule for future updates, because of uncertainty about our funding. Instead, to clarify our future plans to support the software, we have added a paragraph to the discussion. The revised paragraph appears here.

   > GLIMMPSE has been supported by multiple grants from the United States National Institutes of Health (NIH), as described in the Acknowledgments. We hope

> that such funding will continue. However, in the current funding environment, depending only on the NIH may not promote sufficient stability of the software. We have two plans for sustaining the software. First, we hope to leverage external funding to produce command-line callable, end-to-end Python libraries. We will encourage users to access the libraries at our GitHub repository. Second, we hope to explore opportunities to use a business approach to support software development. Setting up a certified B corporation, a societally-focused for-profit corporation, would permit paid sample size consulting to support the free software, under a "freemium model." We plan to survey GLIMMPSE users to examine the market size for such consulting. A stable funding source would permit frequent releases and updates, and standardized bug tracking.

7. *Language & Grammar Section 1.3 ("Literature Review"): Consider rephrasing "Here, we update the literature review..." to "Here, we update our previous review of relevant software..." for clarity.*

   Thanks. Done. Great suggestion.

8. *Language & Grammar Section 4.1 ("Study Vignette"): The text "It is expected that the results for the different workplaces are independent" could be simplified to "We assume the different workplaces are independent."*

   Thanks. Done. We agree that this simplifies the text.

9. *Typographical Corrections Check "CRT Power has the greatest flexibility..."—spacing or punctuation is missing after "Power."*

   Thanks for the catch. I corrected it.

10. *In the references list, confirm consistent formatting of DOIs and parentheses.*

    Thanks for the suggestion. We went through and edited all citations.

11. *Additional Implementation Details In Section 3.2 ("Software Updates"), it might be helpful to briefly mention the average runtime or computational footprint for large cluster sizes (e.g., thousands of clusters). The manuscript states that GLIMMPSE 3 has no practical upper limit up to* $10,000$ *elements. However, a short sentence regarding typical run times or memory usage would reassure readers that such calculations are feasible in practice.*

    An excellent point. We added the following sentence to the last paragraph of Section 3.1.

    > The current version sets a limit of $10,000$ elements per level of clustering. As a test, a run of the example in subsection 4.5 with $10,000$ schools per neighborhood ran just as fast as with 3 schools per neighborhood, in less than 3 seconds clock time.

12. *Conclusion This strong, timely paper describes significant enhancements to a valuable power and sample size calculation tool in linear mixed-model scenarios. The manuscript is well-grounded in appropriate statistical theory, and the examples are engaging and informative. Incorporating the suggested revisions would further strengthen clarity—particularly regarding real-world constraints (e.g., missing data, unequal cluster sizes, or different covariance structures)—and help prospective users better understand the assumptions and usability of GLIMMPSE 3.*

We very much appreciate your comments. We have added detail, as suggested, about missing data, unequal cluster sizes, and different covariance structures. We hope that prospective users will now better understand the assumptions and usability of GLIMMPSE 3.

13. Recommendation *After addressing the points above, I recommend that this manuscript be accepted for publication in PLoS One. The paper makes a significant methodological contribution, and I anticipate its continued use across diverse fields requiring sound power and sample size planning for complex, clustered, and longitudinal data designs.*

    We could not be more grateful for your close reading and excellent suggestions. Many thanks!

Reviewer #2

1. *Add a concise, plain-language summary at the manuscript's start to outline GLIMMPSE 3's purpose, key features, and benefits for non-statisticians, such as clinicians designing clinical trials.*

   Thanks. We have added the following paragraph as Subsection 1.1, Non-technical Overview.

   > GLIMMPSE is an open source, point-and-click, power and sample size software program. It provides power and sample size calculations for designs with continuous outcomes and normally distributed errors. This includes many commonly analyzed outcomes such as cholesterol, blood pressure, or weight. It permits calculation of power and sample size for studies with repeated measures, clustering, or both. It will be useful to scientists conducting observational studies, as well as randomized clinical trials and lab experiments.

2. *Simplify complex sections (e.g., Section 2) with intuitive explanations or visual aids, such as diagrams illustrating the reversible mixed model transformation. Consider moving intricate equations to an appendix to enhance readability for a general scientific audience.*

   We hoped that this paper would be cited by both statisticians and non-statisticians. We thus included multiple equations in Section 2. We thought carefully about your suggestions to move the equations to the appendix, and to provide intuitive explanations or visual aids. Instead, we added a note to Section 1.5, "Organization of the Manuscript" to guide readers. The revised paragraph is as follows, with red indicating changed text.

   > The remainder of the manuscript is organized as follows. Section 2 gives mathematical detail useful for statisticians, and demonstrates the applicability of the software to reversible general linear mixed models. Section 3 describes the updates in the new version, including mathematical details about covariance structures, and algorithmic approaches. Both Sections 2 and 3 may be skipped by readers not interested in technical, mathematical details, without interrupting the narrative flow. Section 4 has five example power or sample size analyses, of increasing complexity. Section 5 compares the accuracy of the results to a Monte Carlo simulation. Section 6 provides details on the number of citers of the software, overall, and per year, and the total funding of United States National Institutes of Health grants which have used the software. Finally, Section 7 summarizes the findings.

3. *Expand Table 1 to include additional power calculation software (e.g., PASS, G*Power, or simulation-based tools like ClusterPower) and incorporate more features, such as open-source status, computational approach and or handling of missing data.*

Thank you. We have extended Table 1 to include both more features and more packages. We also added inclusion and exclusion criteria for the table, and a narrative review of packages not reviewed in the table. The revised paragraphs and table follow.

> Table 1 includes packages that provide power and sample size for designs with clustering, repeated measures, or both clustering and repeated measures. We excluded packages from the table which provide power for single designs and single hypotheses, and packages which are no longer maintained.
>
> Based on these inclusion and exclusion criteria, several packages were excluded from Table 1. We briefly describe each excluded package. Basagaña and Spiegelman [1, 2] describe free software (see `http://www.hsph.harvard.edu/donna-spiegelman/software/optitxs/`) for a range of epidemiological studies. Zhang and Zhang [32] list several R power and sample size modules for designing clinical and cluster randomized trials. Web-based power tools developed by Lenth [18] and Schoenfeld [30] provide power calculations for specific linear models such as t tests, ANOVA, and cross-over studies. Specific examples of packages limited to two or fewer levels of nesting included the Research Methods Resources of the National Institutes of Health (accessed 2025). The Mplus package [21] allows specifying a data analysis and conducting a simulation for a specific design in order to assess power. Finch and Bolin [9] described how to allow up to three more levels of nesting and up to two levels of clustering in Mplus. Iddi and Donohue [11] reviewed power and sample size modules for longitudinal models in R, and described the features of their module LongPower. LongPower allows various complications that can occur in clinical trials. However, the range of designs is limited, and the statistical methods are all large sample approximations, which are of less accuracy with designs with few clusters, which are very common in many fields. The package clusterPower [14, 15] was removed from the CRAN repository, and archived 2023-10-10. The rationale for the removal was that check problems were not corrected on time. This removal is documented at `https://cran.r-project.org/web/packages/clusterPower/index.html`, accessed 05/28/2025. A GitRepo still exists at `https://github.com/Kenkleinman/clusterPower`, but shows a last update at 01/28/2021. For calculations in G*Power [8, 7], a user must specify four of five inputs (the significance level, the "effect size," the number of groups, power, and the number of observations) in order to compute the fifth. The software allows a range of T, F, and χ^2 tests. "Repeated measures" are treated as a 1 or 2 way design, assuming compound symmetry, and using the uncorrected univariate approach to repeated measures. Compound symmetry is a covariance structure with equal correlation and equal variances, based on the assumption of exchangeability within a cluster. Multivariate settings are not allowed.
>
> None of the packages listed in Table 1 have all the functionality of GLIMMPSE 3. CRT Power has the greatest flexibility, but cannot handle repeated measures of the outcome variables, and uses approximate power calculations [3]. PowerUp only allows two treatments [6]. Optimal Design assumes compound symmetric or independent covariance models, even for longitudinal repeated measures [27]. Several packages only allow three levels of nesting. Examples include the National Institutes of Health Research Resource Methods [22] sample size calculators for parallel group-randomized trials, individually randomized trials, and stepped-

	**Packages**											
**Features**	CRT-Power [3]	SPSS [10]	NQuery[25]	PASS [24]	SAS [29]	Stata [31]	Power & Precision [26]	Powerlib [12]	Powerup [6]	SPA-ML [19]	OD [27]	GLIMMPSE [16]
Free								✓	✓	✓	✓	✓
Open Source								✓	✓			✓
Small N Accuracy		✓		✓	✓			✓				✓
Graphical Interface	✓		✓	✓			✓		✓	✓	✓	✓
Multiple Outcomes		✓		✓	✓			✓				✓
Repeated Measures		✓	✓	✓	✓			✓			✓	✓
Multilevel & Repeated								✓				✓
Covariates	✓								✓			✓
Unequal Clusters	✓			✓			✓		✓	✓		
Subgroup Factor								✓				✓
Composite Outcome								✓				✓
Clustering Levels	3	1	2	3	1	1	1	>3	3	3	3	10

Table 1: Review of software for fairly general designs with clustering, repeated measures, or both. Review of software for fairly general designs with clustering, repeated measures, or both. A checkmark indicates that a feature is available. A blank space indicates that the feature is not available.

wedge group-randomized trials. SPA-ML has a limited number of hypotheses, repeated measures, cluster sizes, and number and form of predictors, and uses z-approximations for power [19].

4. *Clearly highlight GLIMMPSE 3's unique strengths for specific study designs, such as longitudinal studies with clustering, to differentiate it from competing tools.*

   A good suggestion. We have added the following text to the literature section, to differentiate the abilities of GLIMMPSE from competing tools.

   > None of the packages listed in Table 1 have all the functionality of GLIMMPSE 3. CRT Power has the greatest flexibility, but cannot handle repeated measures of the outcome variables, and uses approximate power calculations [3]. PowerUp only allows two treatments [6]. Optimal Design assumes compound symmetric or independent covariance models, even for longitudinal repeated measures [27]. Several packages only allow three levels of nesting. Examples include the National Institutes of Health Research Resource Methods [22] sample size calculators for parallel group-randomized trials, individually randomized trials, and stepped-wedge group-randomized trials. SPA-ML has a limited number of hypotheses, repeated measures, cluster sizes, and number and form of predictors, and uses z-approximations for power [19].
   >
   > GLIMMPSE 3 has unique capabilities which no other software package has, including the following:
   >
   > - GLIMMPSE allows for complex covariance matrices, including unstructured covariances for longitudinal repeated measures;
   > - GLIMMPSE permits calculations for up to 10 levels of clustering at each level;
   > - GLIMMPSE is extremely accurate even in small samples;
   > - GLIMMPSE allows computing power and sample size for almost any hypothesis and model in reversible mixed models. This capability permits scientists to compute power and sample size for designs with multiple levels of clustering, repeated measures, or both;
   > - GLIMMPSE allows saving and re-uploading designs. The resulting files can be published for reproducibility; and
   > - GLIMMPSE is fast, taking seconds for each calculation.

5. *Expand Section 7 to thoroughly address the implications of key limitations, such as the inability to manage unequal cluster sizes or missing data, and suggest practical workarounds for researchers.*

   Thanks. We have added a clarifying paragraph. The paragraph follows.

   > GLIMMPSE has some limitations. The use of the reversible model means that all outcomes, predictors and covariates are assumed to have complete data, and that all clusters are the same size. In practice, missing data occurs routinely. In the future, the hope is to incorporate into the software several power and sample size methods that can accommodate missing data, including those of Ringham *et al.* [28] and Kreidler *et al.* [17]. Ringham *et al.* [28] provided power calculations accounting for missing data for a modified test statistic for the multivariate model due to Catellier and Muller [5]. Kreidler *et al.* [17] gave power approximations

for the Kenward and Roger [13] test for the mixed model, which can also allow for missing data. If the data can be assumed to be missing completely at random, scientists can account for missing data using a simple strategy. If the loss-to-followup rate is known to be $x\%$, an adjusted sample size may be calculated by using the full data sample size, divided by the quantity $(1 - x)$. To examine the effect of unequal cluster sizes, scientists can compute bounds on power or sample size. If both the minimum and maximum expected cluster sizes are known, one can compute power and sample size under the assumptions of the minimum and maximum cluster sizes. The true power and sample size will be between the power and sample size estimates for the minimum and maximum cluster sizes.

6. *Include details on computational performance, such as processing times for large clusters or numerical stability for high-dimensional matrices, to inform users about practical usability.*

   We addressed this comment in two ways.

   First, in Section 3, we provide a discussion of the updates to the algorithm. The approach essentially makes the computations invariant to cluster size. The computations use the minimal dimension sufficient statistics.

   Second, in Section 5 we have added additional data about timing. The revised paragraphs and table follow.

   Total CPU time was assessed for GLIMMPSE calculations and Monte Carlo simulations using timing in the Python code. The timing values reflect calculations and simulations performed on a 2017 MacBook Pro, with 3.1 GHz Quad-Core Intel Core i7, and 16GB RAM, under the operating system MacOS Catalina Version 10.15.7. Timing was assessed on power calculations which were computed using the Python libraries, without the web interface. The timing values thus do not include time for HTTP processing by the web interface.

   Accuracy and timing results for the five examples appear in Table 2.

Example #	Calculation CPU Time (seconds $\times 10^{-4}$)	Total Simulation CPU Time (seconds)	GLIMMPSE 3 Backend Calculation	Mean of Simulation Results	Lower Bound of 95% CI	Upper Bound of 95% CI
1	9.0	0.37	0.909	0.910	0.905	0.916
2	7.8	0.50	0.906	0.902	0.896	0.908
3	<1	0.67	0.659	0.657	0.648	0.666
4	<1	0.67	0.909	0.905	0.899	0.911
5	5.8	0.60	0.792	0.796	0.788	0.804

Table 2: Accuracy and Timing, Compared to Simulation.

   The maximum absolute deviation from the Monte Carlo simulation was no more than 0.01. Timing results for calculations appear in units of seconds $\times 10^{-4}$. Timing results for simulations appear in seconds, and are thus all about $10,000$ times slower.

7. *Provide detailed descriptions of figures (e.g., Figures 2–6) in the text, clarifying their content and relevance. For instance, explain how Figure 2's interface simplifies specifying cluster structures.*

Thanks for the suggestion. We added descriptions for each of the figures. The revised language for each figure follows.

Revised Language for Figure 2

User test feedback on the user interface and user experience (UI/UX) showed that users were confused by how to enter levels of clustering. In response, we added the graphical feedback shown in Figure 2. The figure shows an example design with children nested within classrooms, classrooms nested within grades, and grades nested within school. The element on the left shows the highest level of clustering. For this example, it is school. Moving to the right in the schematic shows lower levels of clustering. The schematic is telegraphic. It leaves out some of the classrooms within schools, and some of the children within classrooms. By omitting some information, the schematic shows the clustering clearly, but still fits on the screen. The schematic design was developed and finalized after multiple iterations of exchange between the software designers and the users.

Revised Language for Figure 3

A similar user-focused design approach was used to generate the screen allowing the user to select between hypotheses. The interface permits choosing within-independent-sampling-unit hypotheses, between independent-sampling-unit hypotheses, or interaction hypotheses, as shown in Figure 3.

Most of the structure of the design is specified before the hypothesis screen appears. In order, GLIMMPSE prompts users to specify the following features of the study design.

- A study title.
- A choice between power and sample size.
- The test statistic.
- The Type I error rate.
- The study outcome or outcomes.
- The presence and nature of repeated measures of the outcome or outcomes.
- The presence and nature of clustering.
- The presence and nature of fixed predictor or predictors.
- Whether there is a Gaussian covariate.

These features of study design permit the software to identify what kinds of hypotheses are possible. All possible hypotheses are listed on the screen. Radio boxes permit the users to choose the hypothesis for which they are computing power or sample size. The utility and clarity of this screen were enhanced by repeated interactions between the software team and participants in the user studies.

Revised Language for Figure 4

A flowchart for the study is shown in Figure 4. Figure 4 shows a simplified schematic of the study, using standardized flow chart symbols. After study start, workplaces are randomized to workplace training, or no training, and then drinking is evaluated.

Revised Language for Figure 5

A flow chart for the study is shown in Figure 5. The flowchart provides a simplified schematic of the randomized trial. It shows study initiation, randomization to two groups (intervention and standard-of-care), and three longitudinal repeated measures on each study participant, before study end.

Revised Language for Figure 6

An illustration of a time-by-intervention interaction is shown in Figure 6. A hypothesis about a time-by-intervention interaction asks the question, "Does the pattern of response in the outcome to treatment differ over time?" The figure shows a difference in patterns for the two randomization groups over time. Rejecting the null hypothesis of no time-by-intervention interaction corresponds to concluding that the same groups do not significantly differ in their pattern of outcomes over time.

8. *Ensure all tables (e.g., Tables 3–8) have clear captions and footnotes explaining assumptions or fictional elements.*

   We removed all non-fictional elements, as described in the next item. We changed the text to explain all assumptions.

9. *For each example in Section 4, explicitly identify which elements differ from the original studies and provide robust justification for input values (e.g., cite specific studies or meta-analyses for correlations and standard deviations).*

   The examples in Section 4 were developed for a short course we taught. The course was funded by an announcement from the National Institutes of Health Office of Behavioral and Social Sciences. From our experience teaching, we recognized that using study designs published in the behavioral and social sciences made the students more engaged in studying power and sample size analyses. The examples thus mirrored in some ways published studies. We simplified the example studies, both in design and inputs for the power analyses, as didactic tools. It was easier to talk about a standard deviation of 0.3 to a large class, rather than provide the true value of 0.03539. This approach resulted in blend of fictional and non-fictional elements.

   We believe that because our submission focuses on example power and sample size calculations, the mix of fictional and non-fictional elements was distracting. Thus, we have removed all references to the real studies, real outcome variables, and real interventions. The studies are now completely fictional in every regard, including input values. The revision required multiple tiny changes in each example. For brevity, we have not described each change here.

10. *Remove or justify fictional elements (e.g., VegF genotypes in Example 5) to strengthen the credibility and realism of the examples*

    Thanks for the suggestion. We have now removed all fictional elements.

11. *Simplify the methodology for identifying NIH-funded studies in Section 6.1 and clearly explain why certain funding mechanisms were excluded to improve transparency.*

    We moved the search criteria to an appendix. In addition, we changed the search criteria to include all NIH funding mechanisms. We have thoroughly revised this section, to include new funding and citation information obtained since the initial submission of the document.

12. *Reframe or remove the comparison to Bioconductor, emphasizing GLIMMPSE 3's unique contributions to clinical trial and study design instead.*

Done. We revised the paragraph as shown.

> Use statistics, citations, and United States National Institutes of Health (NIH) funding of studies citing GLIMMPSE continue to rise, showing robust demand. Power and sample size software will always show fewer citations than sites which aggregate routines for data analysis. Yet each citation of our software represents the design of an entire study, and thus represents the investment of great amounts of time and money. This is perhaps best illustrated by an example. One group citing our software, [4], described a randomized controlled trial of a high-intensity interval training intervention in patients with chronic stroke (NCT03760016). Boyne et al. [4] cited funding from R01HD093694, which came in at over 3.3 million United States dollars.

13. *Emphasize how GLIMMPSE 3 supports researchers in designing robust clinical trials or observational studies in fields like oncology, cardiology, or public health. For example, connect Example 5 (vessel tortuosity) to its potential applications in cancer research to illustrate practical impact.*

    We removed the discussion of tortuosity in Example 5 when we removed all fictional elements, and analogies to published studies.

    To illustrate the practical impact, we included a list of citers in Supplementary Appendix C: Software Citers. The list of citers provides a qualitative record of the impact of the software on disparate fields. We added a paragraph in the Discussion to emphasize this. The paragraph follows.

    > One measure of the utility of the GLIMMPSE software is the wide variety of fields of science in which our citers work. GLIMMPSE has been cited in fields ranging from cardiology to memory, from studies of sheep hematology to randomized trials in elder care. While we counted citations per year, and funding accrued by citers, the actual citations of GLIMMPSE citers give qualitative evidence of the wide impact on scientific research of the software program.

14. *Discuss how GLIMMPSE 3 can be applied to observational studies or, if feasible, non-Gaussian outcomes to appeal to a diverse readership, ensuring the software's utility across various research contexts.*

    We clarified that GLIMMPSE 3 can be applied to observational studies. We added the following lines to the first paragraph in the Discussion.

    > GLIMMPSE provides power and sample size calculations for a wide range of designs. While only randomized controlled trials are given in the examples, the package can also be used to compute power and sample size for observational studies , including those with longitudinal repeated measures, clustering, or both.

    It is a limitation of GLIMMPSE that it cannot be applied to either binary or count data. We added a sentence about this to the limitations paragraph in the discussion.

    > GLIMMPSE has some limitations. GLIMMPSE applies only to data with Gaussian (normal) errors. It does not provide power and sample size computations for binary outcome data, nor for count data. We hope to add these features in the future. The use of the reversible model means that all outcomes, predictors and covariates are assumed to have complete data, and that all clusters are the

same size. In practice, missing data occurs routinely. In the future, the hope is to incorporate into the software several power and sample size methods that can accommodate missing data, including those of Ringham *et al.* [28] and Kreidler *et al.* [17]. Ringham *et al.* [28] provided power calculations accounting for missing data for a modified test statistic for the multivariate model due to Catellier and Muller [5]. Kreidler *et al.* [17] gave power approximations for the Kenward and Roger [13] test for the mixed model, which can also allow for missing data. If the data can be assumed to be missing completely at random, scientists can account for missing data using a simple strategy. If the loss-to-followup rate is known to be $x\%$, an adjusted sample size may be calculated by using the full data sample size, divided by the quantity $(1 - x)$. To examine the effect of unequal cluster sizes, scientists can compute bounds on power or sample size. If the both the minimum and maximum expected cluster sizes are known, one can compute power and sample size under the assumptions of the minimum and maximum cluster sizes. The true power and sample size will be between the power and sample size estimates for the minimum and maximum cluster sizes.

Reviewer #3

1. *The authors describe relevant, practical information concerning the use of GLIMMPSE 3, the latest version of GLIMMPSE, a free, web-based, open-source software tool, which calculates power and sample size for general linear mixed models with Gaussian errors. This tool and supporting information concerning its use provide much-needed support for the research community in helping to navigate the complexities of power estimations and corresponding sample size calculations, which are fundamental not only in grant applications, but also in helping to estimate the predictive value of study results toward confirmation of tested hypotheses. The paper is very well-organized and well-written. I anticipate that this excellent work will be greatly appreciated by the research community.*

   Many thanks for your kind words.

2. *P.6. Equation 16 is actually 3 equations and Equation 17, 4 equations. For the sake of consistency perhaps it would be best to either label each equation with its own number or skip the labeling altogether for those equations.*

   I have renumbered the equations, so that each one has its own number.

3. *P.9. I do not find Figure 1 in the version of the manuscript that I downloaded, only the figure caption "Figure 1: Overview of the GLIMMPSE 3 Architecture." I presume that the authors intend to insert a figure similar to Figure 1 from their 2013 article on GLIMMPSE.*

   The pictures were uploaded to the cite, and appeared in the submission as separate files. We regret that you didn't see it. We have reproduced the figure here for your convenience.

Figure 1: Overview of the GLIMMPSE 3 Architecture.

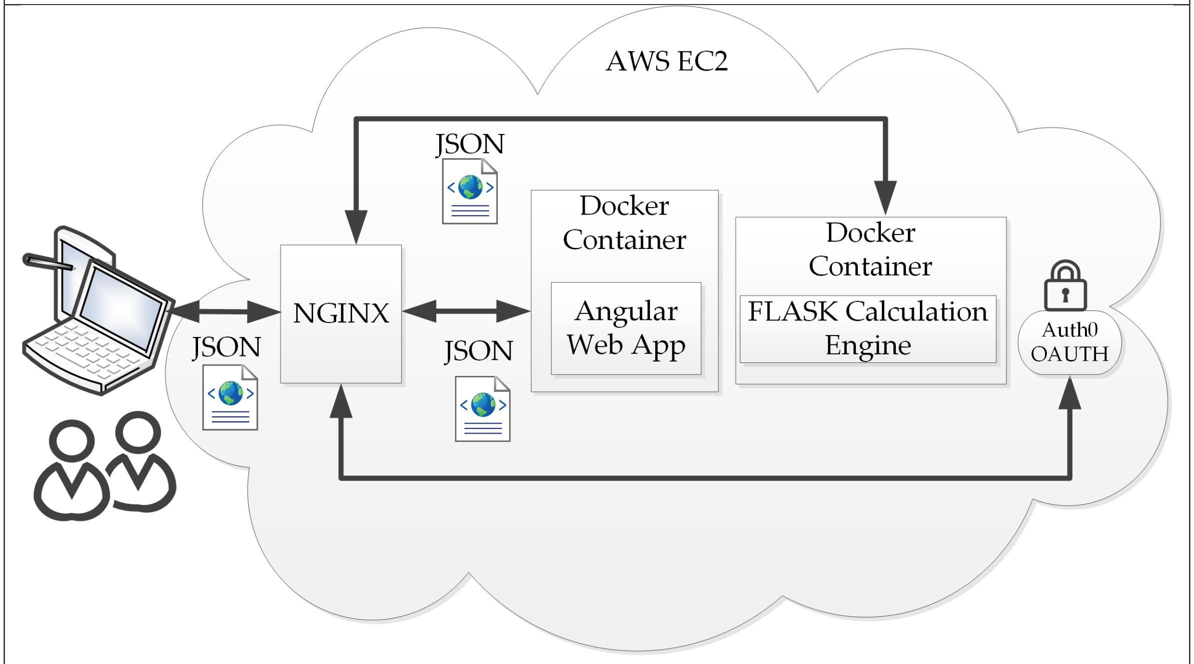

4. *P.16. "a English" → "an English".*

   Done. Thanks.

5. *P.23. Please consider dropping the 3 from the title of section 6 in "GLIMMPSE 3 Use Statistics": That section appears to discuss statistics for all prior versions of GLIMMPSE.*

   Done. Thanks.

References

[1] Basagaña, X., Liao, X., and Spiegelman, D. (2011). Power and sample size calculations for longitudinal studies estimating a main effect of a time-varying exposure. Statistical Methods in Medical Research, 20(5):471–487.

[2] Basagaña, X. and Spiegelman, D. (2010). Power and sample size calculations for longitudinal studies comparing rates of change with a time-varying exposure. Statistics in Medicine, 29(2):181–192.

[3] Borenstein, M., Hedges, L., and Rothstein, H. (2023). CRT-Power.

[4] Boyne, P., Billinger, S. A., Reisman, D. S., Awosika, O. O., Buckley, S., Burson, J., Carl, D., DeLange, M., Doren, S., Earnest, M., Gerson, M., Henry, M., Horning, A., Khoury, J. C., Kissela, B. M., Laughlin, A., McCartney, K., McQuaid, T., Miller, A., Moores, A., Palmer, J. A., Sucharew, H., Thompson, E. D., Wagner, E., Ward, J., Wasik, E. P., Whitaker, A. A., Wright, H., and Dunning, K. (2023). Optimal Intensity and Duration of Walking Rehabilitation in Patients With Chronic Stroke: A Randomized Clinical Trial. JAMA Neurology, 80(4):342–351.

[5] Catellier, D. J. and Muller, K. E. (2000). Tests for Gaussian repeated measures with missing data in small samples. Statistics in Medicine, 19(8):1101–1114.

[6] Dong, N. and Maynard, R. (2013). PowerUp!: A Tool for Calculating Minimum Detectable Effect Sizes and Minimum Required Sample Sizes for Experimental and Quasi-Experimental Design Studies. Journal of Research on Educational Effectiveness, 6(1):24–67. Publisher: Routledge _eprint: https://doi.org/10.1080/19345747.2012.673143.

[7] Faul, F., Erdfelder, E., Buchner, A., and Lang, A.-G. (2009). Statistical power analyses using G*Power 3.1: Tests for correlation and regression analyses. Behavior Research Methods, 41(4):1149–1160. Company: Springer Distributor: Springer Institution: Springer Label: Springer Number: 4 Publisher: Springer-Verlag.

[8] Faul, F., Erdfelder, E., Lang, A.-G., and Buchner, A. (2007). G*Power 3: a flexible statistical power analysis program for the social, behavioral, and biomedical sciences. Behavior Research Methods, 39(2):175–191.

[9] Finch, H. and Bolin, J. (2016). Multilevel Modeling Using Mplus. Chapman and Hall/CRC, Boca Raton, FL, 1st edition edition.

[10] IBM Corporation (2024). IBM SPSS Software Version 31.0. Accessed May 15, 2025.

[11] Iddi, S. and Donohue, M. C. (2022). The R Journal: Power and Sample Size for Longitudinal Models in R – The longpower Package and Shiny App. The R Journal, 14(1):264–282.

[12] Johnson, J. L., Muller, K. E., Slaughter, J. C., Gurka, M. J., Gribbin, M. J., and Simpson, S. L. (2009). POWERLIB: SAS/IML Software for Computing Power in Multivariate Linear Models. Journal of Statistical Software, 30(5):http://www.jstatsoft.org/v30/i05.

[13] Kenward, M. G. and Roger, J. H. (2009). An improved approximation to the precision of fixed effects from restricted maximum likelihood. Computational Statistics & Data Analysis, 53(7):2583–2595.

[14] Kleinman, K. and Huang, S. S. (2016). Calculating Power by Bootstrap, with an Application to Cluster-Randomized Trials. EGEMS (Washington, DC), 4(1):1202.

[15] Kleinman, K., Sakrejda, A., Moyer, J., Nugent, J., Reich, N., and Obeng, D. (2021). clusterPower: Power Calculations for Cluster-Randomized and Cluster-Randomized Crossover Trials.

[16] Kreidler, S. M., Muller, K. E., Grunwald, G. K., Ringham, B. M., Coker-Dukowitz, Z. T., Sakhadeo, U. R., Barón, A. E., and Glueck, D. H. (2013). GLIMMPSE: Online Power Computation for Linear Models with and without a Baseline Covariate. Journal of Statistical Software, 54(10):i10.

[17] Kreidler, S. M., Ringham, B. M., Muller, K. E., and Glueck, D. H. (2021). A power approximation for the Kenward and Roger Wald test in the linear mixed model. PLOS ONE, 16(7):e0254811. Publisher: Public Library of Science.

[18] Lenth, R. V. (2009). Russ Lenth's power and sample size page.

[19] Moerbeek, M. and Teerenstra, S. (2015). Power Analysis of Trials with Multilevel Data. Chapman and Hall/CRC, Boca Raton, 1st edition edition.

[20] Muller, K. E., Lavange, L. M., Ramey, S. L., and Ramey, C. T. (1992). Power Calculations for General Linear Multivariate Models Including Repeated Measures Applications. Journal of the American Statistical Association, 87(420):1209–1226.

[21] Muthén, L. K. and Muthén, B. O. (2023). Mplus Programs.

[22] National Institutes of Health (2022). Research Methods Resources.

[23] National Institutes of Health (2025). Research Methods Resources.

[24] NCSS.com (2024). Statistical Software | Sample Size Software | NCSS.

[25] NQUERY (2024). Optimize clinical trial designs with nQuery.

[26] PowerAndPrecision (2024). Power Analysis Sample Size Calculation Statistical Software.

[27] Raudenbush, S. W., Spybrook, J., Congdon, R., Liu, X., Martinez, A., Bloom, H., and Hill, C. (2011). Optimal Design Software.

[28] Ringham, B. M., Kreidler, S. M., Muller, K. E., and Glueck, D. H. (2016). Multivariate test power approximations for balanced linear mixed models in studies with missing data. Statistics in Medicine, 35(17):2921–2937.

[29] SAS Institute Inc. (2013). SAS software, version 9.4.

[30] Schoenfeld, D. A. (2007). Sample Size Calculators.

[31] StataCorp, L.P. (1985). Stata.

[32] Zhang, Q., Spybrook, J., and Unlu, F. (2020). Examining Design and Statistical Power for Planning Cluster Randomized Trials Aimed at Improving Student Science Achievement and Science Teacher Outcomes. AERA Open, 6(3):233285842093952.
